# Differentiating Catalysis in the Dearomative [4 + 2]-Cycloaddition Involving Enals and Heteroaromatic Aldehydes

**DOI:** 10.1021/acs.orglett.1c04328

**Published:** 2022-01-18

**Authors:** Aleksandra Topolska, Sebastian Frankowski, Łukasz Albrecht

**Affiliations:** Institute of Organic Chemistry, Faculty of Chemistry, Lodz University of Technology, Żeromskiego 116, 90-924 Łódź, Poland

## Abstract

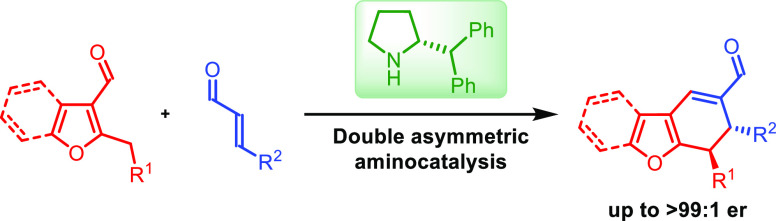

In this paper, the application of differentiating catalysis in the [4 + 2]-cycloaddition between 2-alkyl-3-formylheteroarenes and α,β-unsaturated aldehydes is described. Within the developed approach, the same aminocatalyst is employed for the independent activation of both starting materials, differentiating their properties via LUMO-lowering and HOMO-rising principles. By the combination of dearomative dienamine activation with iminium ion chemistry high enantio- and diastereoselectivity of the doubly asymmetric process was accomplished. Selected transformations of products were also demonstrated.

Stereocontrolled synthesis of specific structural motifs is one of the most important tasks in contemporary organic chemistry.^1^ Within this area of research, double-asymmetric synthesis constitutes an interesting approach.^2^ It leads to the formation of a new stereogenic center by the utilization of two enantiopure substrates bearing a chiral auxiliary unit. When appropriate enantiomers of substrates are employed (“match” case), the chirality of both reagents synergistically enhances the stereochemical reaction outcome (Scheme 1). The main drawback of the approach concerns its atom- and step-economy as chiral auxiliaries are used in stoichiometric amounts and must be introduced and removed in additional, time-consuming procedures. A much easier strategy is based on the catalytic generation of chiral intermediates that participate in the reaction as no additional synthetic protocols are required. The main tool used for this type of asymmetric synthesis is synergistic catalysis where two different catalysts independently activate two substrates, thus providing chiral intermediates capable of participating in a given transformation.^3^ On the contrary, the pathway relying on the activation of both substrates by two molecules of the same catalyst is much less explored.^4^ In this type of approach, electronic properties of substrates are enhanced and at the same time differentiated via the formation of both LUMO-lowered and HOMO-raised reactive intermediates. Therefore, such catalytic activation can be referred to as differentiating catalysis. This strategy offers many benefits including operational simplicity, yet it is more challenging as substrates employed must possess functional groups of similar properties, thus resulting in competitive reaction pathways.

**Scheme 1:**
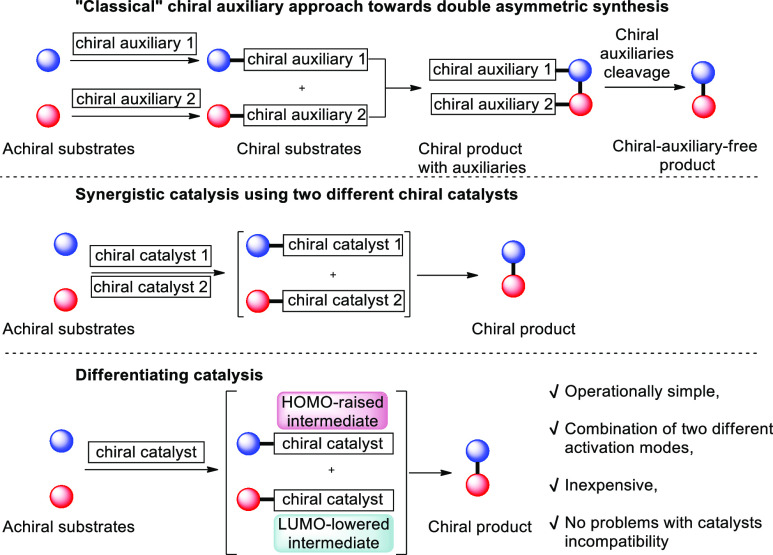
Double Asymmetric Synthesis: Classical and Catalytic Approaches

Since the turn of the millennium, aminocatalysis has proven extremely useful in synthetic methodology, allowing for the stereoselective functionalization of numerous types of carbonyl compounds.^5^ Among different strategies provided by this methodology, HOMO-raising enamine activation of aldehydes and ketones occupies a prominent position. Identification of polyenamine-mediated processes gave access to novel stereoselective methods of functionalization of unsaturated carbonyl compounds with dienamine and trienamine chemistry providing significant synthetic opportunities.^6^ Over the past few years, novel approaches toward generation of polyenamines from (hetero)aromatic compounds have been identified with aminocatalytic dearomative strategies paving new directions in the development of the field. Interestingly, diverse heteroaromatic carbonyl compounds can be transformed with dearomatization into polyenamines under aminocatalytic conditions (Scheme 2, top).^7^ Notably, dienamines derived from 2,3-disubstituted heteroaromatic compounds are prone to the reaction with electrophiles providing either alkylation products^7f,7i,7j^ or more rarely undergoing cycloaddition^7e,7g^ (Scheme 2, middle).

**Scheme 2:**
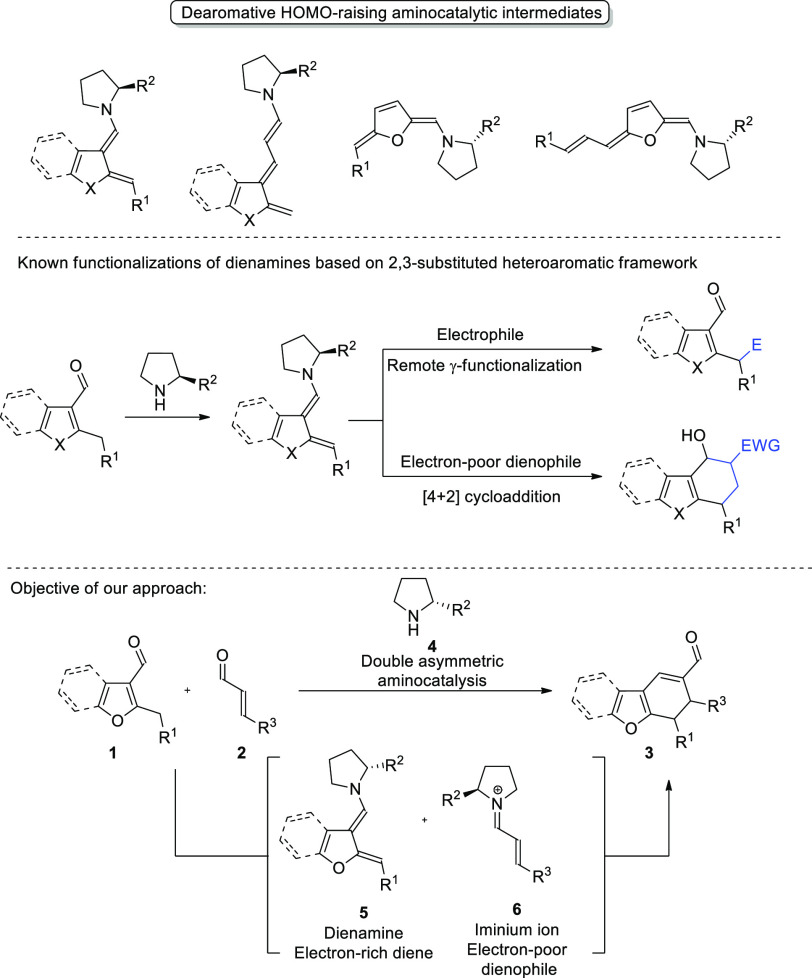
Polyenamine Species Generated from Heteroaromatic Aldehydes and Their Functionalization

Given the interesting possibilities provided by differentiating catalysis, the task of development of dearomative dienamine-mediated [4 + 2]-cycloaddition was undertaken (Scheme 2, bottom). It was envisioned that catalytically generated iminium ions **6** derived from α,β-unsaturated aldehydes **2** might serve as an appropriate electron-poor dienophile for this reaction. It was anticipated that the utilization of selected aminocatalyst should be beneficial for two independent processes: formation of dearomatized dienamine **5** and the previously mentioned iminium ion **6**. Chiral intermediates thus obtained should be prone to react in the Diels–Alder cycloaddition, and double-asymmetric catalysis concept should result in enhancement of stereoselectivity of the process.^8^

Herein, we present our studies on the aminocatalytic [4 + 2]-cycloaddition between dienamines **5** (generated from heteroaromatic aldehydes **1**) and iminium ions **6** (derived from enals **2**) realized according to differentiating catalysis principles. Dearomative Diels–Alder reaction proceeded efficiently, providing enantioenriched heteroaromatic derivatives **3**. Utilization of the obtained product in selected transformations was also demonstrated.

Optimization studies were performed using 2-benzylfuran-3-carbaldehyde **1a** and *trans*-cinnamaldehyde **2a** as model reactants (Table 1). The initial experiment, performed with Hayashi–Jørgensen catalyst **4a** in dichloromethane, showed that the desired enantioenriched product **3a** was formed; nevertheless, the conversion and diastereoselectivity of the reaction were unsatisfactory (Table 1, entry 1). Utilization of amine **4b** provided only traces of cycloadduct **3a**; therefore, further organocatalyst screening proved necessary (Table 1, entry 2). Inspired by our previous work,^4f^ (*R*)-2-benzhydrylpyrrolidine **4c** was used as catalyst, which gave similar results as **4a**, but with enhancement of diastereoselectivity (Table 1, entry 3). Therefore, **4c** was chosen as the most suited catalyst, and subsequent optimization studies were focused on the influence of the relative ratio of substrates on the cycloaddition outcome (Table 1, entries 3–5). The best results were obtained when 1.2-fold excess of **2a** was used, providing 43% conversion within 24 h (Table 1, entry 5). Therefore, the additive screening covering the effect of basic, acidic, or amphiprotic additives was carried out (Table 1, entries 6–8). The highest reaction enhancement was observed when benzoic acid was used as cocatalyst, providing the desired product in 80% yield after 24 h (Table 1, entry 6). Further optimization studies were devoted to the choice of the most suited solvent for the studied cycloaddition (Table 1, entry 9–11). Et_2_O proved the best as it afforded the product **3a** in high chemical yield and with excellent stereoselectivity, thus determining the final reaction conditions (Table 1, entry 11). It is worth mentioning that the developed reaction was readily realized in a 20-fold higher scale, providing cycloadduct **3a** with similar results (Table 1, entry 12).

**Table 1:**
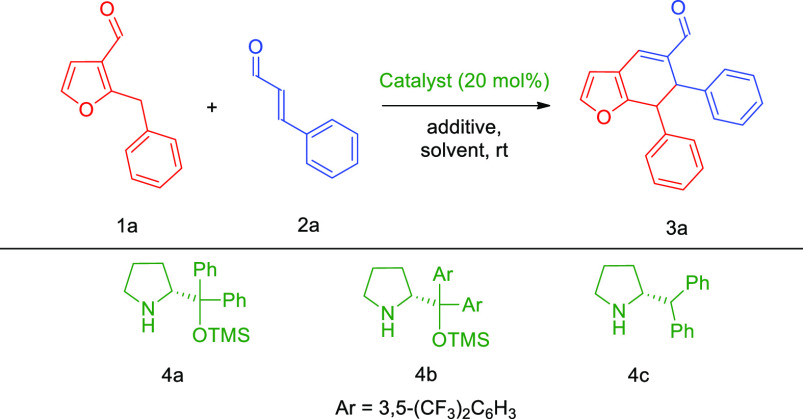
Differentiating Catalysis in the [4 + 2]-Cycloaddition Optimization Studies^a^

entry	cat.	solvent	conv^b^ (%)	dr^c^	er^d^
1	**4a**	CH_2_Cl_2_	34	12:1	>99:1
2	**4b**	CH_2_Cl_2_	trace		
3	**4c**	CH_2_Cl_2_	32	>20:1	>99:1
4^e^	**4c**	CH_2_Cl_2_	25	>20:1	
5^f^	**4c**	CH_2_Cl_2_	43	>20:1	
6^f^^,^^g^	**4c**	CH_2_Cl_2_	92 (80)	>20:1	>99:1
7^f^^,^^h^	**4c**	CH_2_Cl_2_	32	>20:1	
8^f^^,^^i^	**4c**	CH_2_Cl_2_	85	>20:1	
9^f^^,^^g^	**4c**	CHCl_3_	39	>20:1	
10^f^^,^^g^	**4c**	toluene	30	>20:1	
11^f^^,^^g^	**4c**	Et_2_O	94 (95)	>20:1	>99:1
12^f^^,^^g^^,^^j^	**4c**	Et_2_O	96 (91)	>20:1	>99:1

a The reactions were performed in 0.05 mmol scale using equimolar amounts of **1a** and **2a** in 0.2 mL of the solvent.

b Determined by ^1^H NMR spectroscopy of a crude reaction mixture after 24 h. The isolated yield is shown in parentheses.

c Determined by ^1^H NMR spectroscopy of a crude reaction mixture.

d Determined by chiral stationary phase UPC.^2^

e Reaction performed using **1a** (1.2 equiv) and **2a** (1.0 equiv).

f Reaction performed using **1a** (1.0 equiv) and **2a** (1.2 equiv).

g Reaction performed with PhCO_2_H (40 mol %) as additive.

h Reaction performed with triethylamine (40 mol %) as additive.

i Reaction performed with 4-dimethylaminobenzoic acid (40 mol %) as additive.

j Reaction performed on a 1 mmol scale.

With the optimized reaction conditions in hand, studies on the scope and limitations of the method were undertaken. In the first part, the influence of the substitution pattern of α,β-unsaturated aldehydes **2** on the developed cycloaddition was investigated (Scheme 3). Transformation was unbiased toward the presence of both electron-withdrawing and electron-donating substituents on the aromatic ring in **2** (Scheme 3, compounds **3b**–**d**). Interestingly, aldehyde **2b** bearing a nitro group in the *para* position gave access to cycloadduct **3b** in good yield and with excellent enantioselectivity, but with diminished diastereoselection. Substitution of the aromatic ring in **2c** with the chlorine atom and in **2d** with methyl group was also well-tolerated in the transformation affording product **3c** and **3d** with satisfactory results. Further examples showed that the stereoselectivity of the method is not influenced by the position of substituents on the aromatic ring in enals **2** (Scheme 3, products **3e**–**g**) as various methoxy-functionalized aldehydes **2e**–**g** reacted smoothly, providing cycloadducts **3e**–**g** with excellent enantio- and diastereocontrol. It is worth noting that introduction of the double substitution pattern on the aryl ring in **2h** was also possible, but product **3h** was obtained with lower enantioselectivity. Furthermore, the *trans*-3-(2-furyl)acrolein **2i** was also utilized in this transformation, yielding product **3i** decorated with a heteroaryl substituent. Unfortunately, hex-2-enal did not participate in the reaction, indicating that aliphatic α,β-unsaturated aldehydes were difficult substrates for the process.

**Scheme 3:**
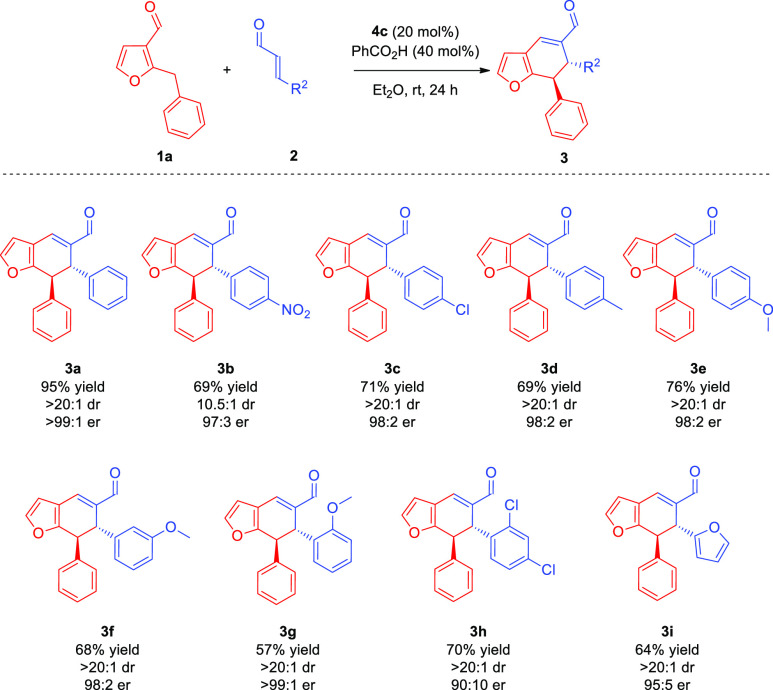
Differentiating Catalysis in the [4 + 2]-Cycloaddition: α,β-Unsaturated Aldehyde **2** Scope

The next step of scope studies was focused on the utilization of structurally diversified heteroaryl aldehydes **1** (Scheme 4). Aldehyde **1b**, containing an electron-acceptor substituent at the *para* position of the phenyl ring, led to the product **3j** with satisfactory yield, diastereoselectivity, and very good enantioselectivity. In the case of aldehydes **3k** and **3m** bearing methyl groups on the phenyl ring, the substituent position had a slight effect on the reaction results - for both 2- and 4-substituted substrates **1c** and **1e**, a significant decrease in the diastereoselectivity and yield was observed, while the enantioselectivity was not affected. Nevertheless, substrate **1d** with a strongly electron-donating methoxy group at the *meta*-position of the phenyl ring gave access to product **3l** with decreased enantioselectivity. It is worth noting that allyl-substituted aldehyde **1f** was also employed in the cycloaddition providing **3n** as a single diastereoisomer in high yield. To our delight, further scope expansion was possible by the utilization of benzofuran-based aldehyde **1g**, which reacted smoothly to give cycloadduct **3o** with good enantioselectivity. Unfortunately 2-butyl-3-furfural and 2-methyl-3-formylbenzofuran were not reactive under the optimal reaction conditions. Moreover, attempts toward utilization of Boc-protected 2-methylindole-3-carbaldehyde and 2-benzylindole-3-carbaldehyde as dienamine precursors were undertaken, but no reactivity was observed in these cases.

**Scheme 4:**
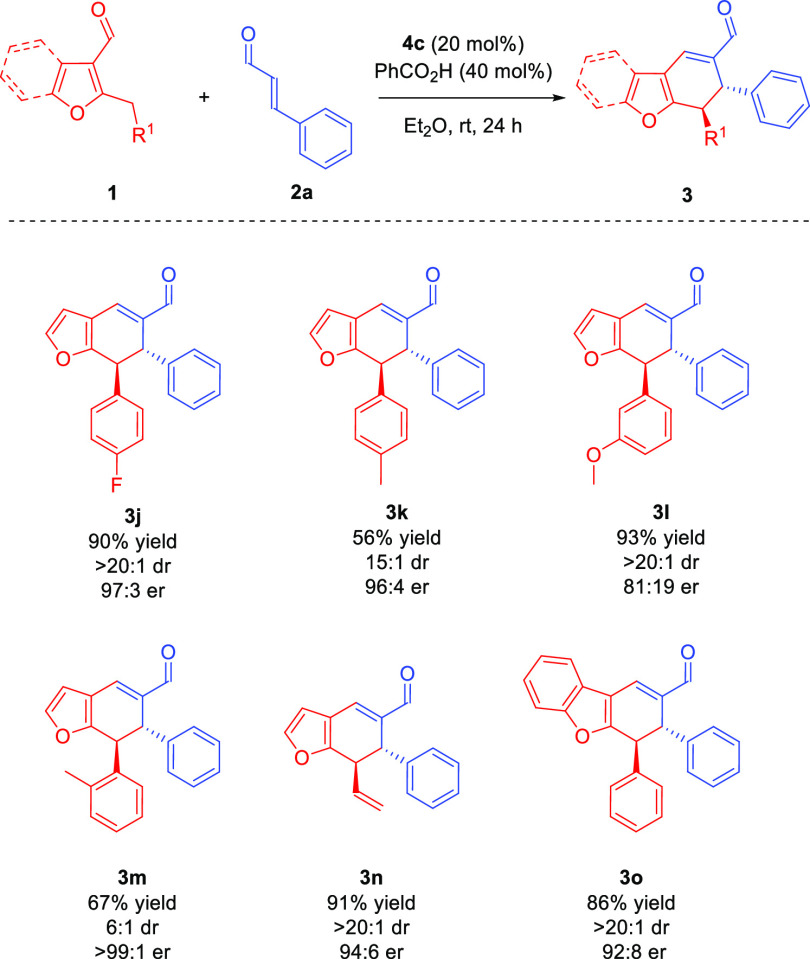
Differentiating Catalysis in the [4 + 2]-Cycloaddition: Heteroaromatic Aldehyde **1** Scope

Subsequent investigations were focused on the synthetic applications of the obtained product **3a** (Scheme 5). Reduction using NaBH_4_ provided alcohol **7** in 78% yield. Moreover, cerium chloride promoted the reaction with *o*-phenylenediamine, affording chiral diazepine **8**. In these two cases, reactions proceeded with full preserevation of the stereochemical composition of **3a**, as products **7** and **8** were obtained as single diastereomers. It was also demonstrated that cycloadduct **3a** can be transformed using DDQ as an oxidant into a highly functionalized benzofuran-5-carbaldehyde **9** with loss of all stereogenic centers.

**Scheme 5:**
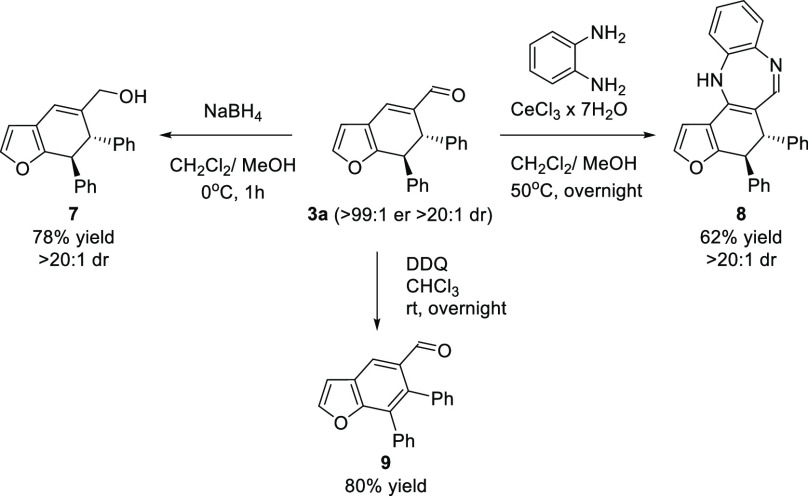
Selected Transformations of the Product **3a**

The absolute configuration of **3a** was determined by the X-ray analysis of a single crystal as (6*S*,7*S*) (see the Supporting Information for further details). The stereochemistry of products **3b**–**o** was assigned by analogy given the assumption that changes in the substitution pattern of products do not have any impact on the mechanism of the cycloaddition. With the knowledge of the stereochemical reaction outcome, the possible reaction mechanism was proposed (Scheme 6).

**Scheme 6:**
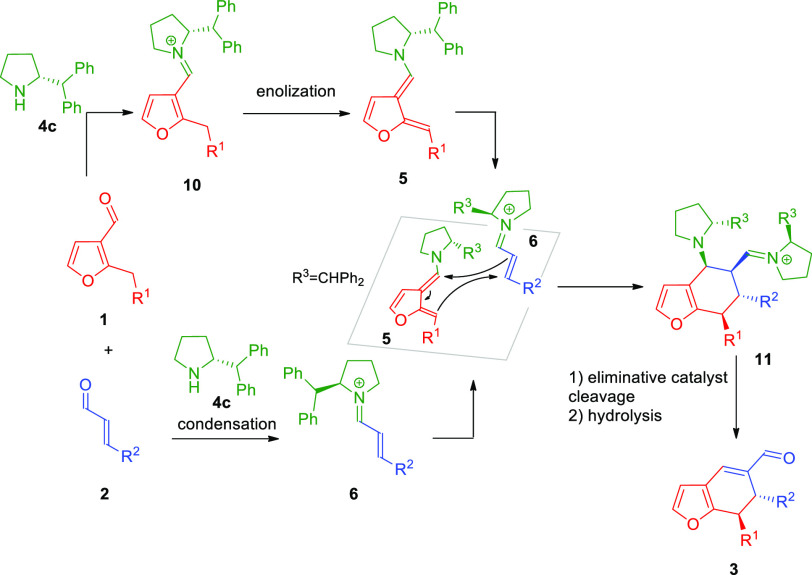
Differentiating Catalysis in the [4 + 2]-Cycloaddition: Mechanistic Considerations

The main role is played by the aminocatalyst **4c** which is responsible for the activation and differentiation of both substrates: (1) it forms the dienamine **5** by dearomatization of heteroaryl aldehyde **1** (HOMO-rising differention) and (2) it independently activates α,β-unsaturated aldehyde **2** through LUMO-lowering iminium ion differentiation. Intermediates **5** and **6** subsequently participate in the *endo*-selective Diels–Alder cycloaddition with the steric interactions between bulky groups in the 2-position of the pyrrolidine units present in both diene **5** and dienophile **6** governing the approach. In the next step, after aromative cycloaddition, iminium ion **11** undergoes eliminative cleavage of catalyst and hydrolysis to obtain target product **3**. Moreover, the postulated mechanism involving two molecules of the catalyst was confirmed by the nonlinear effects studies, which indicated the positive nonlinear effect (for more information, see the Supporting Information).

In conclusion, we demonstrated that differentiating catalysis constitutes a powerful tool for the stereoselective synthesis functionalization of carbonyl compounds. The cycloaddition reaction between **1** and α,β-unsaturated aldehydes **2** was realized through the combination of dearomative dienamine **5** and iminium ion activations, thus providing high enantio- and diastereoselectivity of the transformation. The scope and limitations of the process were carefully studied and the stereochemical model of the reaction was proposed. Moreover, useful synthetic transformations of product **3a** were elaborated.

## Supporting Information Available

Complete experimental procedures, characterization of the products, X-ray data for **3a**, NMR data, and UPC^2^ traces (PDF)

## Accession Codes

CCDC 2103952 contains the supplementary crystallographic data for this paper. These data can be obtained free of charge via www.ccdc.cam.ac.uk/data_request/cif, or by emailing data_request@ccdc.cam.ac.uk, or by contacting The Cambridge Crystallographic Data Centre, 12 Union Road, Cambridge CB2 1EZ, UK; fax: +44 1223 336033.
