# Supplementary material for: Differentiating Catalysis in the Dearomative [4 + 2]-Cycloaddition Involving Enals and Heteroaromatic Aldehydes
Source: Org Lett. 2022 Jan 18;24(3):955–9. doi: 10.1021/acs.orglett.1c04328 (PMC8805123; doi:10.1021/acs.orglett.1c04328)
Supplement: Supplementary file 1 — ol1c04328_si_001.pdf [file ol1c04328_si_001.pdf]

Differentiating Catalysis in the Dearomative [4+2]-Cycloaddition Involving Enals and Heteroaromatic Aldehydes

Aleksandra Topolska,[a] Sebastian Frankowski,[a] and Łukasz Albrecht[a*]

[a] Institute of Organic Chemistry

Faculty of Chemistry

Lodz University of Technology

Żeromskiego 116, 90-924 Łódź, Poland

E-mail: lukasz.albrecht@p.lodz.pl

Contents

1. General methods S2
2. Differentiating catalysis in the [4+2]-cycloaddition – general procedure S3
3. Selective transformations of the product **3a** S10
4. Crystal and X-ray data for **3a** S13
5. Non-linear effects study S16
6. NMR data S17
7. UPC^2 data S35

1. General methods

NMR spectra were acquired on a Bruker Ultra Shield 700 instrument, running at 700 MHz for ^{1}H and 176 MHz for ^{13}C, respectively. Chemical shifts (δ) are reported in ppm relative to residual solvent signals ($CDCl_3$: 7.26 ppm for ^{1}H NMR, 77.16 ppm for ^{13}C NMR). Mass spectra were recorded on a Bruker Maxis Impact quadrupole-time-of-flight spectrometer using electrospray (ES+) ionization (referenced to the mass of the charged species). Analytical thin layer chromatography (TLC) was performed using pre-coated aluminum-backed plates (Merck Kieselgel 60 F254) and visualized by ultraviolet irradiation or Hanessian's stain. Unless otherwise noted, analytical grade solvents and commercially available reagents were used without further purification. For flash chromatography (FC) silica gel (Silica gel 60, 230-400 mesh, Fluka). The enantiomeric ratio (er) of the products were determined by Ultra Performance Convergence Chromatography (UPC^2) using Daicel Chiralpak IA column as chiral stationary phases. Aldehydes **2** were synthesized according to the literature procedure.[1] Heteroaromatic aldehydes **1** were prepared from the corresponding starting materials following the literature procedure.[2]

(1) Daubresse, N.; Francesch, C.; Rolando, C. Phase transfer Wittig reaction with 1,3-dioxolan-2-yl-methyltriphenyl phosphonium salts: An efficient method for vinylogation of aromatic aldehydes. *Tetrahedron* **1998**, *54*, 10761-10770.
(2) Bojanowski, J.; Skrzyńska, A.; Albrecht, A. Dearomatizative and Decarboxylative Reaction Cascade in the Aminocatalytic Synthesis of 3,4-Dihydrocoumarins. *Asian J. Org. Chem.* **2019**, *8*, 844-848.

2. Differentiating catalysis in the [4+2]-cycloaddition – general procedure

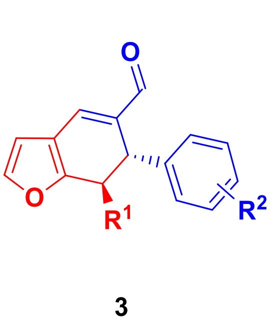

In an ordinary 4 mL glass vial equipped with a magnetic stirring bar α,β-unsaturated aldehyde **2** (0.12 mmol, 1.2 equiv.) and heteroaromatic aldehyde **1** (0.1 mmol, 1 equiv.) were dissolved in Et_2O (0.4 mL) and catalyst **4c** (4.7 mg, 0.02 mmol, 0.2 equiv.) and benzoic acid (4.9 mg, 0.04 mmol, 0.4 equiv.) were added and the reaction mixture was stirred in room temperature for indicated time. The progress of the reaction was controlled by ^{1}H NMR spectroscopy. After full conversion of the starting material **1**, the reaction mixture was directly subjected to column chromatography on silica gel (hexanes : diethyl ether 85:15) to afford pure products **3a-o**.

(6*S*,7*S*)-6,7-Diphenyl-6,7-dihydrobenzofuran-5-carbaldehyde 3a

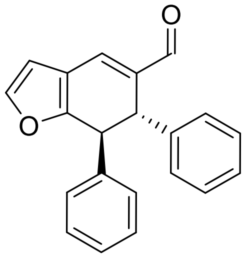

Following the general procedure, using **1a** (18.6 mg), product **3a** (>20:1 dr in a crude reaction mixture) was isolated in 95% yield (28.5 mg) as light-yellow solid (m.p. = 148 – 150 ˚C after recrystallization from hexane/diethyl ether mixture). ^{1}H NMR (700 MHz, $CDCl_3$) δ 9.54 (s, 1H), 7.46 (s, 1H), 7.42 (d, *J* = 2.0 Hz, 1H), 7.31 – 7.28 (m, 3H), 7.28 – 7.26 (m, 3H), 7.25 – 7.21 (m, 2H), 7.05 – 7.04 (m, 2H), 6.60 (d, *J* = 2.0 Hz, 1H), 4.44 (s, 1H), 4.34 (s, 1H).^{13}C NMR (176 MHz, $CDCl_3$) δ 191.3, 157.6, 144.3, 142.6, 141.6, 139.7, 136.5, 129.2 (2C), 129.0 (2C), 127.6, 127.4, 127.0 (2C), 126.8 (2C), 117.6, 108.6, 47.4, 47.1. The er was determined by UPC^2 using a chiral Chiralpack IA column gradient from 100% CO_2 up to 40%; *i*-PrOH, flow rate = 2.2 mL/min, l = 295 nm) tR = 3.1 min (major), 2.9 min (minor), (>99:1 er). $[\alpha]_D^{23}$ = + 625.9 (c = 1.0, $CHCl_3$). HRMS (ESI) m/z $[M+H]^+$ Calcd. for $C_{21}H_{17}O_2^+$: 301.1224; found: 301.1230.

(6*S*,7*S*)-6-(4-Nitrophenyl)-7-phenyl-6,7-dihydrobenzofuran-5-carbaldehyde 3b

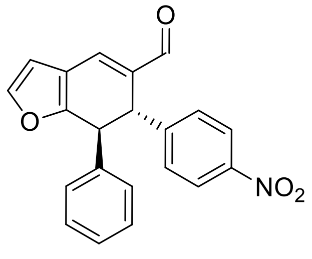

Following the general procedure, using **1a** (18.6 mg), product **3b** (10.5:1 dr in a crude reaction mixture) was isolated in 69% yield (23.8 mg) as light-yellow oil. ^{1}H NMR (700 MHz, $CDCl_3$) δ 9.56 (s, 1H), 8.15 – 8.13 (m, 2H), 7.55 (s, 1H), 7.47 (d, *J* = 2.0 Hz, 1H), 7.43 (d, *J* = 8.6 Hz, 2H), 7.34 – 7.31 (m, 2H), 7.30 – 7.27 (m, 1H), 7.06 – 7.04 (m, 2H), 6.65 (d, *J* = 2.0 Hz, 1H), 4.53 (s, 1H), 4.31 (s, 1H). ^{13}C NMR (176 MHz, $CDCl_3$) δ 190.9, 157.0, 149.7, 147.4, 144.9, 140.7, 140.4, 135.3, 129.3 (2C), 128.0 (2C), 127.9, 126.7 (2C), 124.3 (2C), 117.7, 108.7, 47.0, 46.9. The er was determined by UPC^2 using a chiral Chiralpack IA column gradient from 100% CO_2 up to 40%; *i*-PrOH, flow rate = 2.2 mL/min, l = 358 nm) tR = 4.0 min (major), 3.7 min (minor), (97:3 er). $[\alpha]_D^{22}$ = + 776.8 (c = 1.0, $CHCl_3$). HRMS (ESI) m/z $[M+H]^+$ Calcd. for $C_{21}H_{16}NO_4^+$: 346.1074; found: 346.1085.

(6*S*,7*S*)-6-(4-Chlorophenyl)-7-phenyl-6,7-dihydrobenzofuran-5-carbaldehyde 3c

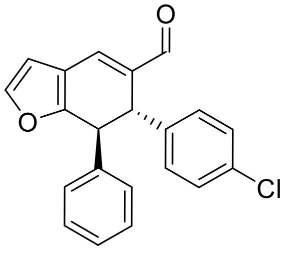

Following the general procedure, using **1a** (18.6 mg), product **3c** (>20:1 dr in a crude reaction mixture) was isolated in 71% yield (23.7 mg) as light-yellow oil. ^{1}H NMR (700 MHz, $CDCl_3$) δ 9.52 (s, 1H), 7.46 (s, 1H), 7.42 (d, *J* = 2.0 Hz, 1H), 7.30 – 7.27 (m, 2H), 7.25 – 7.21 (m, 3H), 7.19 – 7.17 (m, 2H), 7.04 – 7.01 (m, 2H), 6.60 (d, *J* = 2.0 Hz, 1H), 4.39 (s, 1H), 4.27 (s, 1H). ^{13}C NMR (176 MHz, $CDCl_3$) δ 191.1, 157.3, 144.5, 141.2, 141.0, 139.8, 136.1, 133.2, 129.2 (2C), 129.1 (2C), 128.4 (2C), 127.7, 126.7 (2C), 117.6, 108.6, 47.3, 46.4. The er was determined by UPC^2 using a chiral Chiralpack IA column gradient from 100% CO_2 up to 40%; *i*-PrOH, flow rate = 2.2 mL/min, l = 330 nm) tR = 3.6 min (major), 3.2 min (minor), (98:2 er). $[\alpha]_D^{23}$ = + 753.3 (c = 1.0, $CHCl_3$). HRMS (ESI) m/z $[M+H]^+$ Calcd. for $C_{21}H_{16}ClO_2^+$: 335.0761; found: 335.0769.

(6*S*,7*S*)-7-Phenyl-6-(p-tolyl)-6,7-dihydrobenzofuran-5-carbaldehyde 3d

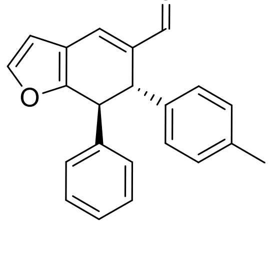

Following the general procedure, using **1a** (18.6 mg), product **3d** (>20:1 dr in a crude reaction mixture) was isolated in 69% yield (21.7 mg) as light-yellow oil. ^{1}H NMR (700 MHz, $CDCl_3$) δ 9.53 (s, 1H), 7.43 (s, 1H), 7.41 (d, *J* = 2.0 Hz, 1H), 7.28 – 7.27 (m, 2H), 7.25 – 7.21 (m, 1H), 7.16 –

7.13 (m, 2H), 7.07 – 7.06 (m, 2H), 7.05 – 7.04 (m, 2H), 6.59 (d, *J* = 2.0 Hz, 1H), 4.40 (d, *J* = 1.4 Hz, 1H), 4.32 (s, 1H), 2.30 (s, 3H). ^{13}C NMR (176 MHz, $CDCl_3$) δ 191.3, 157.6, 144.3, 141.6, 139.7, 139.5, 137.0, 136.6, 129.6 (2C), 129.1 (2C), 127.5, 126.9 (2C), 126.8 (2C), 117.6, 108.6, 47.5, 46.7, 21.2. The er was determined by UPC^2 using a chiral Chiralpack IA column gradient from 100% CO_2 up to 40%; *i*-PrOH, flow rate = 2.2 mL/min, l = 230 nm) tR = 3.3 min (major), 3.0 min (minor), (98:2 er). $[\alpha]_D^{23}$ = + 329.3 (c = 1.0, $CHCl_3$). HRMS (ESI) m/z $[M+H]^+$ Calcd. for $C_{22}H_{19}O_2^+$: 315.1380; found: 315.1388.

(6*S*,7*S*)-6-(4-Methoxyphenyl)-7-phenyl-6,7-dihydrobenzofuran-5-carbaldehyde 3e

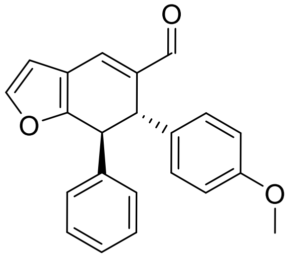

Following the general procedure, using **1a** (18.6 mg), product **3e** (>20:1 dr in a crude reaction mixture) was isolated in 76% yield (25.1 mg) as light-yellow oil. ^{1}H NMR (700 MHz, $CDCl_3$) δ 9.52 (s, 1H), 7.41 – 7.40 (m, 2H), 7.28 – 7.27 (m, 2H), 7.24 – 7.21 (m, 1H), 7.18 – 7.16 (m, 2H), 7.05 – 7.01 (m, 2H), 6.79 – 6.77 (m, 2H), 6.59 (d, *J* = 2.1 Hz, 1H), 4.37 (s, 1H), 4.30 (s, 1H), 3.76 (s, 3H). ^{13}C NMR (176 MHz, $CDCl_3$) δ 191.4, 158.9, 157.6, 144.3, 141.6, 139.3, 136.8, 134.8, 129.1 (2C), 128.1 (2C), 127.5, 126.8 (2C), 117.6, 114.3 (2C), 108.6, 55.4, 47.5, 46.3. The er was determined by UPC^2 using a chiral Chiralpack IA column gradient from 100% CO_2 up to 40%; *i*-PrOH, flow rate = 2.2 mL/min, l = 215 nm) tR = 3.6 min (major), 3.3 min (minor), (98:2 er). $[\alpha]_D^{23}$ = + 593.4 (c = 1.0, $CHCl_3$). HRMS (ESI) m/z $[M+H]^+$ Calcd. for $C_{22}H_{19}O_3^+$: 331.1329; found: 331.1337.

(6*S*,7*S*)-6-(3-Methoxyphenyl)-7-phenyl-6,7-dihydrobenzofuran-5-carbaldehyde 3f

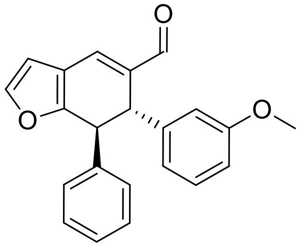

Following the general procedure, using **1a** (18.6 mg), product **3f** (>20:1 dr in a crude reaction mixture) was isolated in 68% yield (22.4 mg) as light-yellow oil. ^{1}H NMR (700 MHz, $CDCl_3$) δ 9.53 (s, 1H), 7.45 (s, 1H), 7.40 (d, *J* = 2.0 Hz, 1H), 7.30 – 7.26 (m, 2H), 7.25 – 7.22 (m, 1H), 7.19 – 7.17 (m, 1H), 7.06 – 7.03 (m, 2H), 6.88 – 6.86 (m, 1H), 6.81 (d, *J* = 2.0 Hz, 1H), 6.78 – 6.76 (m, 1H), 6.58 (d, *J* = 2.2 Hz, 1H), 4.41 (s, 1H), 4.35 (s, 1H), 3.76 (s, 3H). ^{13}C NMR (176 MHz, $CDCl_3$) δ 191.3, 160.0, 157.6, 144.3, 144.1, 141.6, 139.8, 136.3, 130.0, 129.2 (2C), 127.5, 126.8 (2C), 119.4, 117.5, 113.2, 112.3, 108.6, 55.3, 47.3, 47.0. The er was determined by UPC^2 using a chiral Chiralpack IA column gradient from 100% CO_2 up to

40%; *i*-PrOH, flow rate = 2.2 mL/min, l = 251 nm) tR = 3.2 min (major), 3.1 min (minor), (98:2 er). $[\alpha]_D^{23}$ = + 468.0 (c = 1.0, $CHCl_3$). HRMS (ESI) m/z $[M+H]^+$ Calcd. for $C_{22}H_{19}O_3^+$: 331.1329; found: 331.1338.

(6*S*,7*S*)-6-(2-methoxyphenyl)-7-phenyl-6,7-dihydrobenzofuran-5-carbaldehyde 3g

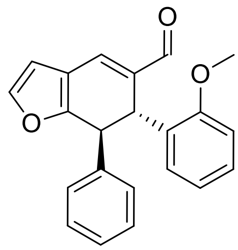

Following the general procedure, using **1a** (18.6 mg), product **3g** (>20:1 dr in a crude reaction mixture) was isolated in 57% yield (18.8 mg) as light-yellow oil. ^1H NMR (700 MHz, $CDCl_3$) δ 9.54 (s, 1H), 7.60 (s, 1H), 7.36 – 7.35 (d, *J* = 1.8 Hz, 1H), 7.29 – 7.26 (m, 2H), 7.23 – 7.19 (m, 1H), 7.20 – 7.19 (m, 1H), 7.10 – 7.06 (m, 2H), 6.95 – 6.94 (m, 1H), 6.88 (d, *J* = 1.8 Hz, 1H), 6.78 – 6.74 (m, 1H), 6.59 (s, 1H), 4.86 (s, 1H), 4.28 (s, 1H), 4.00 (s, 3H). ^{13}C NMR (176 MHz, $CDCl_3$) δ 191.3, 158.2, 157.0, 144.1, 141.6, 141.2, 135.4, 128.8 (2C), 128.4, 128.3, 127.2, 127.0 (2C), 126.9, 120.4, 117.7, 111.2, 108.5, 55.6, 45.9, 40.7. The er was determined by UPC^2 using a chiral Chiralpack IA column gradient from 100% CO_2 up to 40%; *i*-PrOH, flow rate = 2.2 mL/min, l = 339 nm) tR = 3.2 min (major), 3.0 min (minor), (>99:1 er). $[\alpha]_D^{23}$ = + 402.2 (c = 1.0, $CHCl_3$). HRMS (ESI) m/z $[M+H]^+$ Calcd. for $C_{22}H_{19}O_3^+$: 331.1329; found: 331.1340.

(6*R*,7*S*)-6-(2,4-Dichlorophenyl)-7-phenyl-6,7-dihydrobenzofuran-5-carbaldehyde 3h

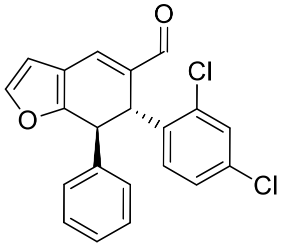

Following the general procedure, using **1a** (18.6 mg), product **3h** (>20:1 dr in a crude reaction mixture) was isolated in 70% yield (25.8 mg) as light-yellow oil. ^1H NMR (700 MHz, $CDCl_3$) δ 9.57 (s, 1H), 7.43 (s, 1H), 7.40 (d, *J* = 2.0 Hz, 1H), 7.34 – 7.31 (m, 1H), 7.28 – 7.26 (m, 2H), 7.25 – 7.20 (m, 1H), 7.05 – 7.01 (m, 2H), 6.56 (d, *J* = 1.9 Hz, 1H), 6.22 (dd, *J* = 3.2, 1.9 Hz, 1H), 5.96 (d, *J* = 3.2 Hz, 1H), 4.61 (s, 1H), 4.60 (s, 1H). ^{13}C NMR (176 MHz, $CDCl_3$) δ 190.8, 157.8, 154.1, 144.2 (2C), 142.0 (2C), 140.5, 140.0, 133.5, 129.1, 127.6 (2C), 126.9 (2C), 117.2, 110.3, 108.7, 105.8, 43.9, 40.5. The er was determined by UPC^2 using a chiral Chiralpack IA column gradient from 100% CO_2 up to 40%; ACN, flow rate = 2.2 mL/min, l = 235 nm) tR = 3.0 min (major), 3.4 min (minor), (90:10 er). $[\alpha]_D^{23}$ = + 591.1 (c = 1.0, $CHCl_3$). HRMS (ESI) m/z $[M+H]^+$ Calcd. for $C_{21}H_{15}Cl_2O_2^+$: + 369.0444; found: 369.0455.

(6*R*,7*S*)-6-(Furan-2-yl)-7-phenyl-6,7-dihydrobenzofuran-5-carbaldehyde 3i

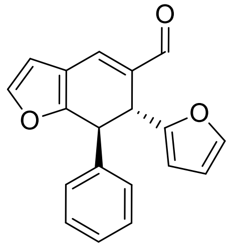

Following the general procedure, using **1a** (18.6 mg), product **3i** (>20:1 dr in a crude reaction mixture) was isolated in 64% yield (18.6 mg) as light-yellow oil. ^{1}H NMR (700 MHz, $CDCl_3$) δ 9.55 (s, 1H), 7.64 (s, 1H), 7.48 (d, *J* = 2.0 Hz, 1H), 7.39 (d, *J* = 2.1 Hz, 1H), 7.29 – 7.27 (m, 2H), 7.25 – 7.22 (m, 1H), 7.14 – 7.12 (m, 2H), 7.05 (dd, *J* = 8.4, 2.0 Hz, 1H), 6.91 (d, *J* = 8.4 Hz, 1H), 6.60 (d, *J* = 2.1 Hz, 1H), 4.88 (s, 1H), 4.20 (s, 1H). ^{13}C NMR (176 MHz, $CDCl_3$) δ 190.7, 157.4, 144.6, 141.2, 140.4, 136.5, 135.3, 134.5, 133.7, 130.3, 129.2, 128.8, 127.7, 127.3, 127.2, 117.6, 108.5, 46.0, 42.8. The er was determined by UPC^2 using a chiral Chiralpack IA column gradient from 100% CO_2 up to 40%; ACN, flow rate = 2.2 mL/min, l = 237 nm) tR = 2.5 min (major), 2.7 min (minor), (95:5 er). $[\alpha]_D^{23}$ = + 614.1 (c = 1.0, $CHCl_3$). HRMS (ESI) m/z $[M+H]^+$ Calcd. for $C_{19}H_{15}O_3^+$: 291.1016; found: 291.1022.

(6*S*,7*S*)-7-(4-Fluorophenyl)-6-phenyl-6,7-dihydrobenzofuran-5-carbaldehyde 3j

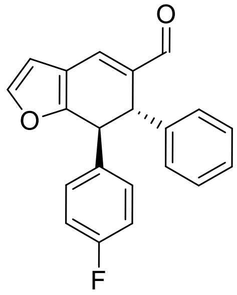

Following the general procedure, using **1b** (20.4 mg), product **3j** (>20:1 dr in a crude reaction mixture) was isolated in 90% yield (28.6 mg) as light-yellow oil. ^{1}H NMR (700 MHz, $CDCl_3$) δ 9.56 (s, 1H), 7.48 (s, 1H), 7.45 (d, *J* = 2.1 Hz, 1H), 7.31 – 7.22 (m, 5H), 7.06 – 6.97 (m, 4H), 6.63 (d, *J* = 2.1 Hz, 1H), 4.41 (s, 1H), 4.35 (s, 1H). ^{13}C NMR (176 MHz, $CDCl_3$) δ 191.3, 162.8 (d, *J* = 246.1 Hz, 2C), 161.5, 157.3, 144.5, 142.3, 139.6, 137.3, 136.3, 129.0, 128.4 (d, *J* = 8.2 Hz, 2C), 127.5, 127.0, 117.6, 116.0 (d, *J* = 21.2 Hz, 2C), 108.6, 47.2, 46.5. The er was determined by UPC^2 using a chiral Chiralpack IA column gradient from 100% CO_2 up to 40%; *i*-PrOH, flow rate = 2.2 mL/min, l = 244 nm) tR= 3.0 min (major), 2.7 min (minor), (97:3 er). $[\alpha]_D^{23}$ = + 734.4 (c = 1.0, $CHCl_3$). HRMS (ESI) m/z $[M+H]^+$ Calcd. for $C_{21}H_{16}FO_2^+$: 319.1129; found: 319.1141.

(6*S*,7*S*)-6-Phenyl-7-(p-tolyl)-6,7-dihydrobenzofuran-5-carbaldehyde 3k

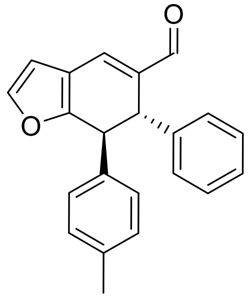

Following the general procedure, using **1c** (20.0 mg), product **3k** (15:1 dr in a crude reaction mixture) was isolated in 56% yield (17.6 mg) as light-yellow oil. ^{1}H NMR (700 MHz, $CDCl_3$) δ 9.54 (s, 1H), 7.45 (s, 1H), 7.41 (dd, *J* = 2.0, 0.6 Hz, 1H), 7.27 (s, 4H), 7.25 – 7.21 (m, 1H), 7.11 – 7.08 (m,

2H), 6.96 – 6.93 (m, 2H), 6.59 (dd, *J* = 2.0, 0.6 Hz, 1H), 4.43 (d, *J* = 1.4 Hz, 1H), 4.33 – 4.30 (s, 1H), 2.32 (s, 3H). ^{13}C NMR (176 MHz, $CDCl_3$) δ 191.3, 157.9, 144.3, 142.6, 139.8, 138.6, 137.2, 136.4, 129.8 (2C), 129.0 (2C), 127.3, 127.0 (2C), 126.6 (2C), 117.5, 108.5, 47.1, 47.0, 21.2. The er was determined by UPC^2 using a chiral Chiralpack IA column gradient from 100% CO_2 up to 40%; *i*-PrOH, flow rate = 2.2 mL/min, l = 230 nm) tR= 3.1 min (major), 2.9 min (minor), (96:4 er). $[\alpha]_D^{23}$ = + 849.4 (c = 1.0, $CHCl_3$). HRMS (ESI) m/z $[M+H]^+$ Calcd. for $C_{22}H_{19}O_2^+$: 315.1380; found: 315.1387.

(6*S*,7*S*)-7-(3-Methoxyphenyl)-6-phenyl-6,7-dihydrobenzofuran-5-carbaldehyde 3l

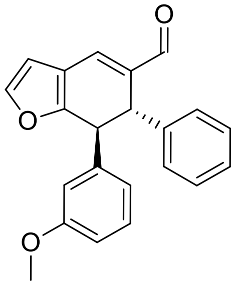

Following the general procedure, using **1d** (21.6 mg), product **3l** (>20:1 dr in a crude reaction mixture) was isolated in 93% yield (30.7 mg) as light-yellow oil. ^{1}H NMR (700 MHz, $CDCl_3$) δ 9.54 (s, 1H), 7.45 (s, 1H), 7.42 (d, *J* = 2.1 Hz, 1H), 7.29 – 7.26 (m, 4H), 7.24 – 7.18 (m, 2H), 6.79 – 6.77 (m, 1H), 6.68 – 6.67 (m, 1H), 6.59 (d, *J* = 2.1 Hz, 1H), 6.58 – 6.57 (m, 1H), 4.44 (s, 1H), 4.32 (s, 1H), 3.77 (s, 3H). ^{13}C NMR (176 MHz, $CDCl_3$) δ 191.3, 160.1, 157.4, 144.4, 143.0, 142.5, 139.7, 136.5, 130.2, 129.0 (2C), 127.4, 127.0 (2C), 119.1, 117.6, 113.0, 112.3, 108.6, 55.3, 47.3, 47.0. The er was determined by UPC^2 using a chiral Chiralpack IA column gradient from 100% CO_2 up to 40%; *i*-PrOH, flow rate = 2.2 mL/min, l = 227 nm) tR = 3.2 min (major), 3.1 min (minor), (81:19 er). $[\alpha]_D^{23}$ = + 692.2 (c = 1.0, $CHCl_3$). HRMS (ESI) m/z $[M+H]^+$ Calcd. for $C_{22}H_{19}O_3^+$: 331.1329; found: 331.1343.

(6*S*,7*S*)-6-Phenyl-7-(o-tolyl)-6,7-dihydrobenzofuran-5-carbaldehyde 3m

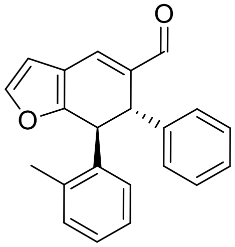

Following the general procedure, using **1e** (20.0 mg), product **3m** (6:1 dr in a crude reaction mixture) was isolated in 67% yield (21.0 mg) as light-yellow oil. ^{1}H NMR (700 MHz, $CDCl_3$) δ 9.51 (s, 1H), 7.44 (d, *J* = 3.6 Hz, 2H), 7.32 – 7.30 (m, 2H), 7.29 – 7.26 (m, 2H), 7.26 – 7.23 (m, 2H), 7.14 (td, *J* = 7.6, 1.3 Hz, 1H), 7.05 (td, *J* = 7.6, 1.3 Hz, 1H), 6.63 (d, *J* = 2.0 Hz, 1H), 6.60 (dd, *J* = 7.6, 1.3 Hz, 1H), 4.59 (d, *J* = 1.3 Hz, 1H), 4.28 (d, *J* = 1.3 Hz, 1H), 2.51 (s, 3H). ^{13}C NMR (176 MHz, $CDCl_3$) δ 191.3, 157.9, 146.9, 144.3, 142.9, 139.5, 139.3, 136.7, 134.9, 131.3, 129.0, 127.4, 127.3, 127.0, 126.6, 126.5, 118.4, 108.5, 85.3, 46.4, 43.1, 27.6. The er was determined by UPC^2 using a chiral Chiralpack IA column gradient from 100% CO_2 up to 40%; *i*-

PrOH, flow rate = 2.2 mL/min, l = 248 nm) tR = 2.7 min (major), 2.9 min (minor), (>99:1 er). $[\alpha]_D^{23}$ = + 496.5 (c = 1.0, $CHCl_3$). HRMS (ESI) m/z $[M+H]^+$ Calcd. for $C_{22}H_{19}O_2^+$: 315.1380; found: 315.1388.

(6*S*,7*R*)-6-Phenyl-7-vinyl-6,7-dihydrobenzofuran-5-carbaldehyde 3n

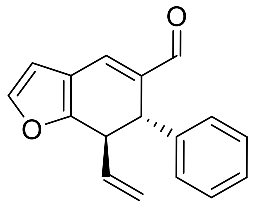

Following the general procedure, using **1f** (13.6 mg), product **3n** (>20:1 dr in a crude reaction mixture) was isolated in 91% yield (22.8 mg) as light-yellow oil. ^{1}H NMR (700 MHz, $CDCl_3$) δ 9.55 (s, 1H), 7.42 (d, *J* = 2.0 Hz, 1H), 7.36 (s, 1H), 7.23 – 7.19 (m, 2H), 7.19 – 7.16 (m, 3H), 6.53 (dd, *J* = 2.0, 0.6 Hz, 1H), 5.85 (ddd, *J* = 17.0, 10.1, 6.7 Hz, 1H), 5.05 (dt, *J* = 10.1, 1.1 Hz, 1H), 4.88 (ddd, *J* = 17.0, 1.4, 1.1 Hz, 1H), 4.28 (d, *J* = 1.1 Hz, 1H), 3.76 (dd, *J* = 6.8, 1.4 Hz, 1H). ^{13}C NMR (176 MHz, $CDCl_3$) δ 191.5, 157.2, 144.1, 141.5, 139.9, 136.9, 136.2, 128.8 (2C), 127.3, 127.1 (2C), 117.0, 115.7, 108.6, 45.4, 43.7. The er was determined by UPC^2 using a chiral Chiralpack IA column gradient from 100% CO_2 up to 40%; ACN, flow rate = 2.2 mL/min, l = 228 nm) tR = 2.5 min (major), 2.1 min (minor), (94:6 er). $[\alpha]_D^{23}$ = + 85.6 (c = 1.0, $CHCl_3$). HRMS (ESI) m/z $[M+H]^+$ Calcd. for $C_{17}H_{15}O_2^+$: 251.1067; found: 251.1074.

(3*S*,4*S*)-3,4-Diphenyl-3,4-dihydrodibenzo[b,d]furan-2-carbaldehyde 3o

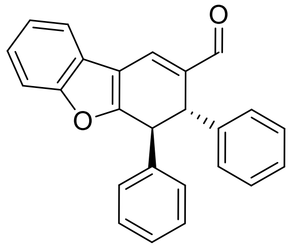

Following the general procedure, using **1g** (23.6 mg), product **3o** (>20:1 dr in a crude reaction mixture) was isolated in 86% yield (30.1 mg) as light-yellow oil. ^{1}H NMR (700 MHz, $CDCl_3$) ^{1}H NMR (700 MHz, $CDCl_3$) δ 9.66 (s, 1H), 7.79 – 7.76 (m, 2H), 7.47 (dt, *J* = 8.1, 0.9 Hz, 1H), 7.39 (td, *J* = 7.5, 1.1 Hz, 1H), 7.36 – 7.34 (m, 1H), 7.32 – 7.28 (m, 4H), 7.28 – 7.26 (m, 1H), 7.26 – 7.21 (m, 3H), 7.17 – 7.14 (m, 2H), 4.56 (d, *J* = 1.3 Hz, 1H), 4.49 (d, *J* = 1.3 Hz, 1H). ^{13}C NMR (176 MHz, $CDCl_3$) δ 191.1, 161.0, 156.3, 142.4, 141.0, 137.4, 136.3, 129.3 (2C), 129.1 (2C), 127.8, 127.5, 127.0 (2C), 126.9 (2C), 125.0, 124.7, 124.1, 119.1, 113.8, 112.2, 47.8, 46.9. The er was determined by UPC^2 using a chiral Chiralpack IA column gradient from 100% CO_2 up to 40%; *i*-PrOH, flow rate = 2.2 mL/min, l = 216 nm) tR = 3.5 min (major), 3.3 min (minor), (92:8 er). $[\alpha]_D^{21}$ = + 395.5 (c = 1.0, MeOH). HRMS (ESI) m/z $[M+H]^+$ Calcd. for $C_{25}H_{19}O_2^+$: 351.1380; found: 351.1389.

3. Selective transformations of the product 3a

3.1. Oxidation of the product 3a to benzofuran-5-carbaldehyde 9

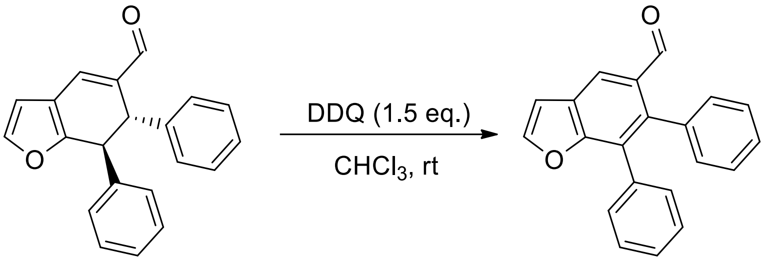

In an ordinary 4 mL glass vial, equipped with a Teflon-coated magnetic stirring bar the aldehyde **3a** (30.1 mg, 0.1 mmol, 1 equiv.) was dissolved in $CHCl_3$ (0.2 mL). Then DDQ (34 mg, 0.15 mmol, 1.5 equiv.) was added and the reaction mixture was stirred at room temperature overnight. After full conversion of the starting material **3a** (as confirmed by ^1H NMR spectroscopy), the reaction mixture was directly subjected to column chromatography on silica gel (eluent: hexanes/diethyl ether 4:1) to afford pure product **9** in 80% yield (23.9 mg) as light-yellow oil.

6,7-Diphenylbenzofuran-5-carbaldehyde 9. ^1H NMR (700 MHz, $CDCl_3$) δ 9.83 (s, 1H), 8.35 (s, 1H), 7.70 (d, *J* = 2.2 Hz, 1H), 7.29 – 7.26 (m, 2H), 7.26 – 7.22 (m, 4H), 7.21 – 7.19 (m, 2H), 7.16 – 7.13 (m, 2H), 6.96 (d, *J* = 2.2 Hz, 1H). ^{13}C NMR (176 MHz, $CDCl_3$) δ 192.7, 156.0, 147.7, 141.2, 136.0, 134.1, 131.8 (2C), 131.1, 130.8 (2C), 128.0 (2C), 127.8 (2C), 127.6, 127.5, 127.3, 126.1, 120.6, 107.7. HRMS (ESI) m/z $[M+H]^+$ Calcd. for $C_{21}H_{15}O_2^+$: 299.1067; found: 299.1074.

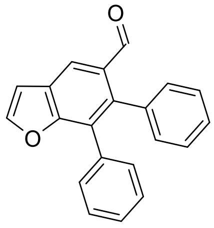

3.2. Synthesis of diazepine derivative 8

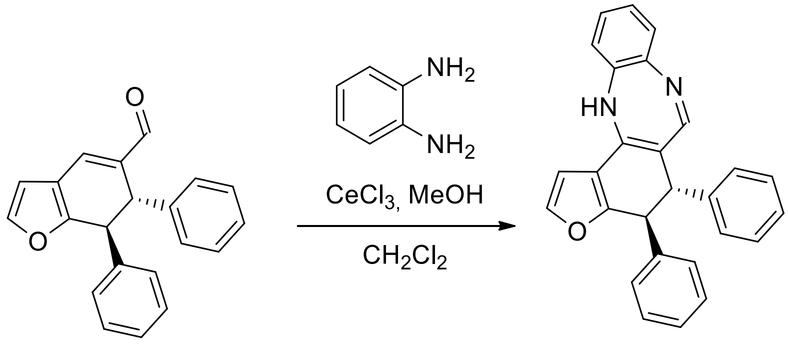

3a **8**

In an ordinary 8 mL glass vial, equipped with a Teflon-coated magnetic stirring bar the aldehyde **3a** (30.1 mg, 0.1 mmol, 1 equiv.) was dissolved in MeOH/CH_2Cl_2 3:1 *v*/*v* (1 mL). Then *o*-phenylenediamine (10.8 mg, 0.1 mmol, 1 equiv.) and $CeCl_3·7H_2O$ (37.2 mg, 0.1 mmol, 1 equiv.) were added and the reaction mixture was stirred at 50 °C for 20 hours. After full conversion of the starting material **3a** (as confirmed by TLC analysis), the reaction mixture was diluted with CH_2Cl_2 (10 mL) and washed with water (2×5 mL), dried over anhydrous sodium sulfate, filtered and concentrated under reduced pressure. The resulting solid was subjected to column chromatography on silica gel (eluent: CH_2Cl_2) to afford pure product **8** (>20:1 dr) in 62% yield (24.0 mg) as light-yellow oil.

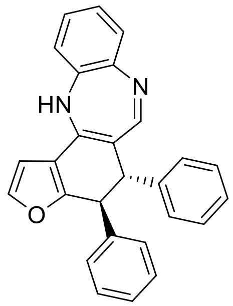

(4*S*,5*S*)-4,5-Diphenyl-5,12-dihydro-4*H*-benzo[*b*]benzofuro[4,5-*e*][1,4]diazepine 8. ^{1}H NMR (700 MHz, DMSO-*d*6) δ 12.57 (s, 1H), 7.72 (s, 1H), 7.65 (d, *J* = 1.9 Hz, 1H), 7.45 (d, *J* = 8.0 Hz, 1H), 7.43 – 7.39 (m, 2H), 7.37 (d, *J* = 8.0 Hz, 1H), 7.30 (t, *J* = 7.7 Hz, 2H), 7.25 (t, *J* = 7.7 Hz, 2H), 7.24 – 7.20 (m, 1H), 7.20 – 7.15 (m, 3H), 7.14 – 7.03 (m, 2H), 6.79 – 6.75 (m, 1H), 4.93 (s, 1H), 4.33 (s, 1H). ^{13}C NMR (176 MHz, DMSO-*d*6) δ 152.6, 151.5, 143.9, 143.4, 142.1, 141.6, 134.7, 128.8 (2C), 128.5 (2C), 127.1 (2C), 127.0, 126.8, 126.7 (2C), 124.7, 122.5, 121.6, 121.3, 118.6, 118.0, 110.7, 108.6, 49.8, 47.1. $[\alpha]_D^{23}$ = + 249.9 (c = 1.0, MeOH). HRMS (ESI) m/z $[M+H]^+$ Calcd. for $C_{27}H_{21}N_2O^+$: 389.1649; found: 389.1644.

3.3. Selective reduction of aldehyde 3a

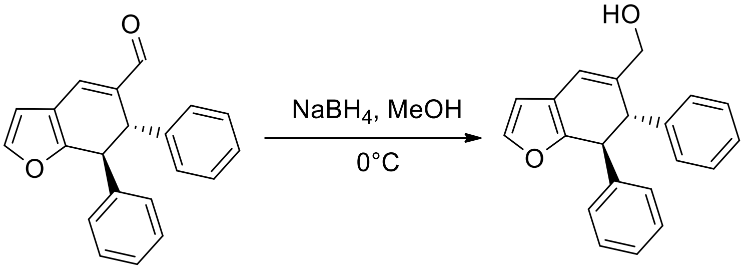

3a **7**

In an ordinary 4 mL glass vial equipped with a magnetic stirring bar the aldehyde **3a** (30.1 mg, 0.1 mmol, 1 equiv.) was dissolved in CH_2Cl_2 (0.2 mL). Then MeOH (0.1 mL) and $NaBH_4$ (15.2 mg, 0.4 mmol, 4 equiv.) were added and the reaction mixture was stirred in room temperature for 30 min. Then the reaction mixture was directly subjected to column chromatography on silica gel (eluent: hexanes/ethyl acetate 4:1) to afford pure product **7** in 78% yield (23.6 mg) as light-yellow oil.

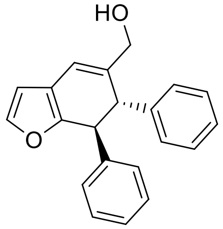

((6*S*,7*S*)-6,7-Diphenyl-6,7-dihydrobenzofuran-5-yl)methanol 7. ^{1}H NMR (700 MHz, $CDCl_3$) δ 7.30 – 7.26 (m, 5H), 7.26 – 7.21 (m, 4H), 7.14 – 7.10 (m, 2H), 6.60 (dt, *J* = 1.5, 0.8 Hz, 1H), 6.43 (dd, *J* = 1.9, 0.6 Hz, 1H), 4.18 (d, *J* = 2.3 Hz, 1H), 4.05 – 4.01 (m, 1H), 3.97 (d, *J* = 13.9 Hz, 1H), 3.80 (d, *J* = 2.4 Hz, 1H). ^{13}C NMR (176 MHz, $CDCl_3$) δ 151.5, 142.8, 142.7, 142.6, 135.7, 129.1 (2C), 129.0 (2C), 127.5 (2C), 127.3, 127.2, 127.1 (2C), 117.7, 117.2, 108.2, 65.3, 52.8, 48.2. HRMS (ESI) m/z $[M+H]^+$ Calcd. for $C_{21}H_{19}O_2^+$: 303.1380; found: 303.1384.

3.3. Enantioselective synthesis of (6*S*,7*S*)-6,7-diphenyl-6,7-dihydrobenzofuran-5-carbaldehyde 3a on a 1 mmol scale

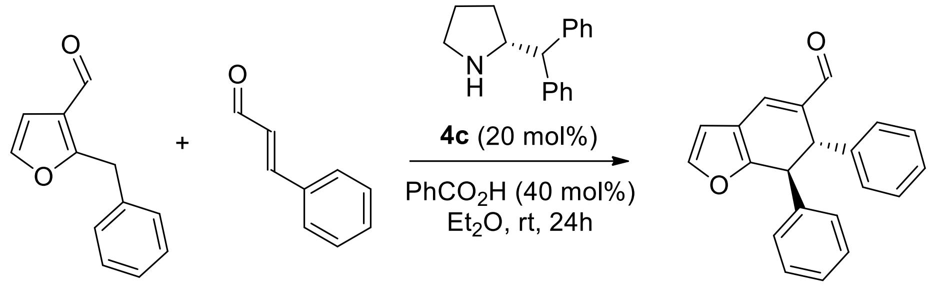

1a **2a** **3a**

In an ordinary 8 mL glass vial equipped with a magnetic stirring bar α,β-unsaturated aldehyde **2a** (158.0 mg, 1.2 mmol, 1.2 equiv.) and heteroaromatic aldehyde **1a** (186.0 mg, 1.0 mmol, 1.0 equiv.) were dissolved in Et_2O (4 mL) and catalyst **4c** (47.0 mg, 0.2 mmol, 0.2 equiv.) and benzoic acid (49.0 mg, 0.4 mmol, 0.4 equiv.) were added and the reaction mixture was stirred in room temperature for indicated time. The progress of the reaction was controlled by ^{1}H NMR spectroscopy. After full conversion of the starting material **1a**, the reaction mixture was directly subjected to column chromatography on silica gel (hexanes : diethyl ether 85:15) to afford pure product **3a** as single diastereoisomer in 91% yield (273.0 mg) as light-yellow solid. Spectral data were in accordance with the previously reported on page S3.

4. Crystal and X-ray data for 3a

The crystal structure of the compound (6*S*,7*S*)-6,7-diphenyl-6,7-dihydrobenzofuran-5-carbaldehyde **3a**, $C_{21}H_{16}O_2$, was established by single-crystal X-ray diffraction at 100 K. The compound crystallizes in the non-centrosymmetric orthorhombic space group $P2_12_12_1$ (Z = 4) and the crystal structure consists of one crystallographically independent formula unit in the unit cell (Figure 1).

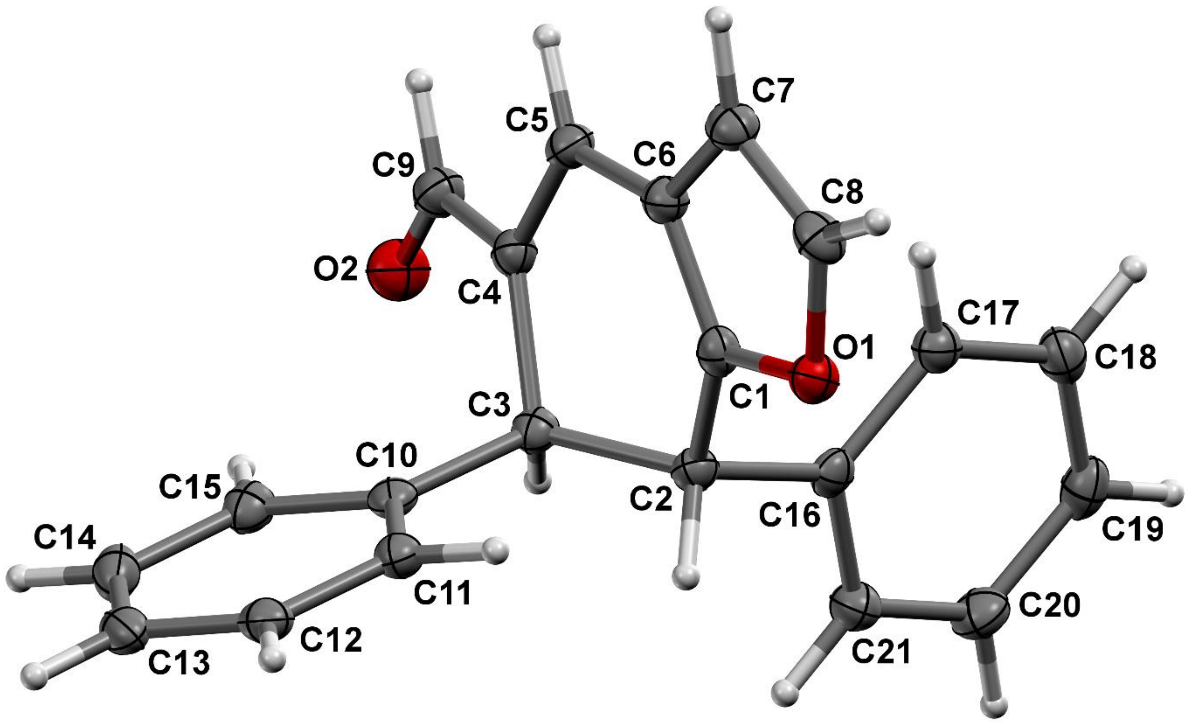

Figure 1. The molecular structure of the compound **3a** at 100 K, with the atom labeling scheme, showing 50% probability displacement ellipsoids. Hydrogen atoms are drawn with an arbitrary radius.

Single crystal X-ray diffraction data were collected at 100 K by the ω-scan technique using a RIGAKU XtaLAB Synergy, Dualflex, Pilatus 300K diffractometer[3] with PhotonJet micro-focus X-ray Source Cu-Kα (λ = 1.54184 Å). Data collection, cell refinement, data reduction and absorption correction were performed using CrysAlis PRO software.[3] The crystal structure was solved by using direct methods with the SHELXT 2018/2 program.[4] Atomic scattering factors were taken from the International Tables for X-ray Crystallography. Positional parameters of non-H-atoms were refined by a full-matrix least-squares method on F^2 with anisotropic thermal

parameters by using the SHELXL 2018/3 program.[5] All hydrogen atoms were found from the difference Fourier maps and for further calculations they were positioned geometrically in calculated positions (C–H = 0.95–1.00 Å) and constrained to ride on their parent atoms with isotropic displacement parameters set to 1.2 times the U_{eq} of the parent atom.

(6*S*,7*S*)-6,7-Diphenyl-6,7-dihydrobenzofuran-5-carbaldehyde **3a:** Formula $C_{21}H_{16}O_2$, orthorhombic, space group $P2_12_12_1$, $Z = 4$, unit cell constants $a = 8.12398(4)$, $b = 10.30996(6)$, $c = 17.96578(9)$ Å, $V = 1504.776(14)$ Å^3. The integration of the data yielded a total of 41425 reflections with θ angles in the range of 4.92 to 66.59°, of which 2662 were independent (R_{int} = 2.92%), and 2642 were greater than $2\sigma(F^2)$. The final anisotropic full-matrix least-squares refinement on F^2 with 209 parameters converged at R_1 = 2.34% and wR_2 = 5.92% for all data. The largest peak in the final difference electron density synthesis was 0.162 e Å^{-3} and the largest hole was -0.144 e Å^{-3}. The goodness-of-fit was 1.055. The absolute configuration was unambiguously established from anomalous scattering, by calculating the *x* Flack parameter[6] of 0.00(3) using 1099 quotients.

CCDC 2103952 contains the supplementary crystallographic data for this paper. These data can be obtained free of charge from The Cambridge Crystallographic Data Centre via www.ccdc.cam.ac.uk/structures

References

(3) Rigaku OD. CrysAlis PRO. Rigaku Oxford Diffraction Ltd, Yarnton, Oxfordshire, England, 2019.

(4) Sheldrick, G. M. SHELXT - integrated space-group and crystal-structure determination. *Acta Cryst.* **2015**, *A71*, 3-8.

(5) Sheldrick, G. M. Crystal structure refinement with SHELXL. *Acta Cryst.* **2015**, *C71*, 3-8.

(6) Parsons, S.; Flack, H. D.; Wagner, T. Use of intensity quotients and differences in absolute structure refinement. *Acta Cryst.* **2013**, *B69*, 249-259.

5. Non-linear effect study

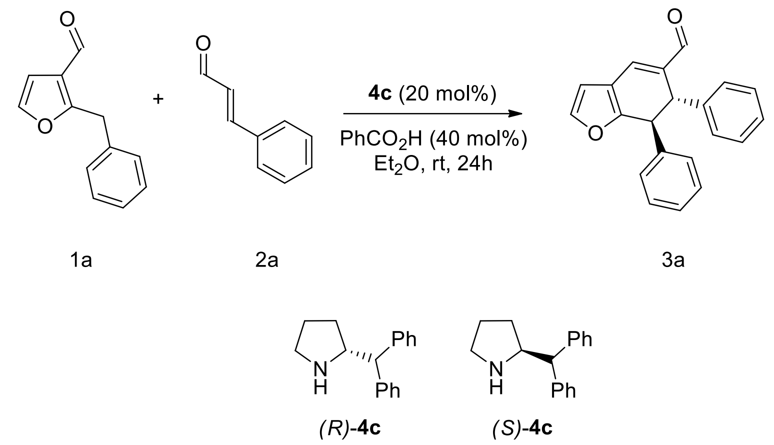

The experiments for non-linear effect study were carried out according to the procedure of the synthesis of **3a**. The catalyst **4c** mixtures with different ee values were prepared by mixing (*S*)-**4c** and (*R*)-**4c** in appropriate ratios (with the (*R*)-**4c** being the major). The ee value of product **3a** was determined by UPC^2, which indicated a non-linear relationship between ee values of products **3a** and amine catalyst **4c**, as shown in the figure below.

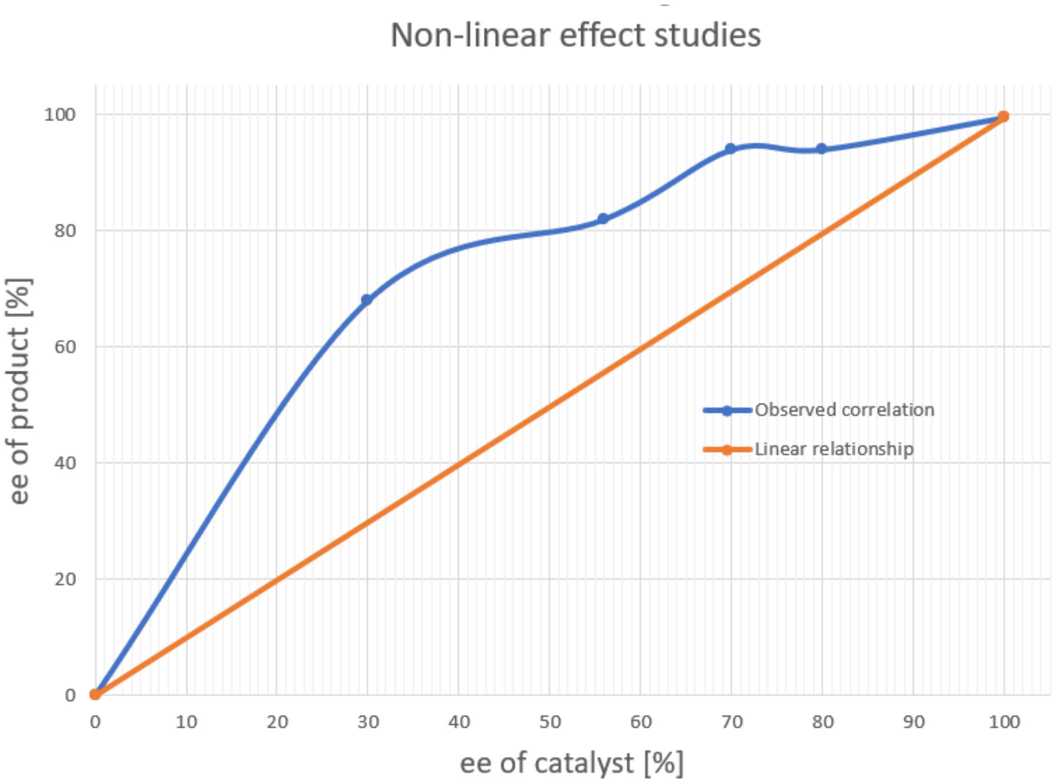

6. NMR Data

(6*S*,7*S*)-6,7-Diphenyl-6,7-dihydrobenzofuran-5-carbaldehyde 3a

^{1}H NMR (700 MHz, $CDCl_3$)

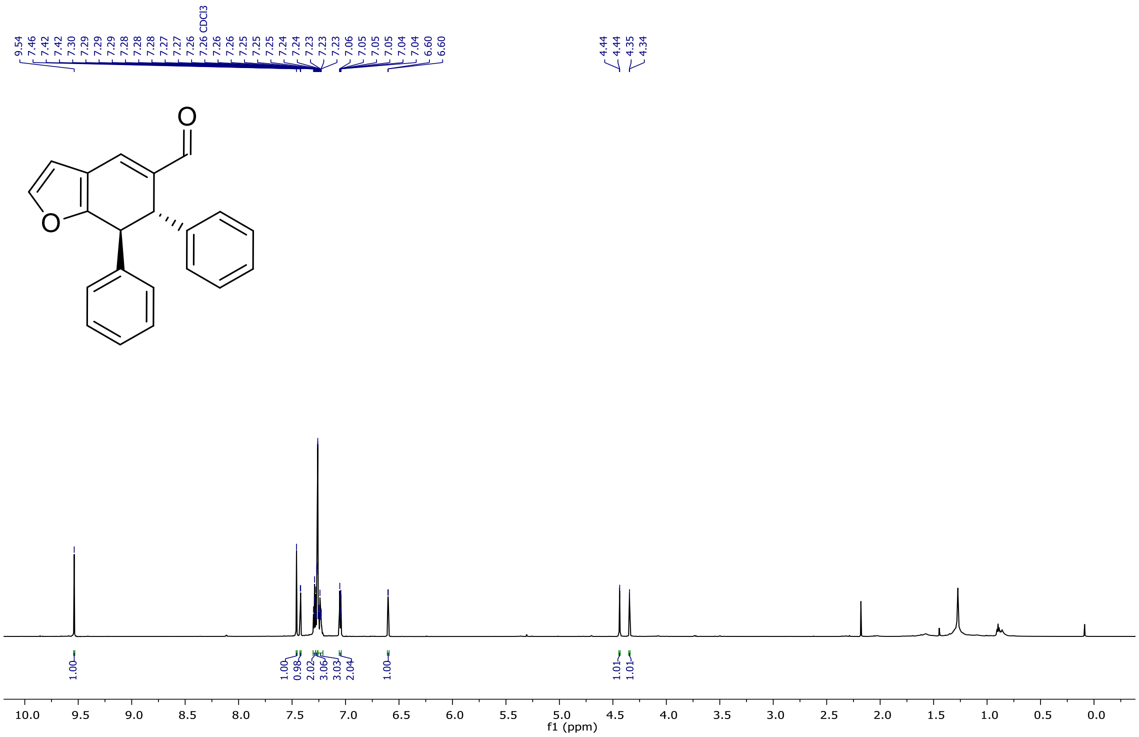

^{13}C NMR (176 MHz, $CDCl_3$)

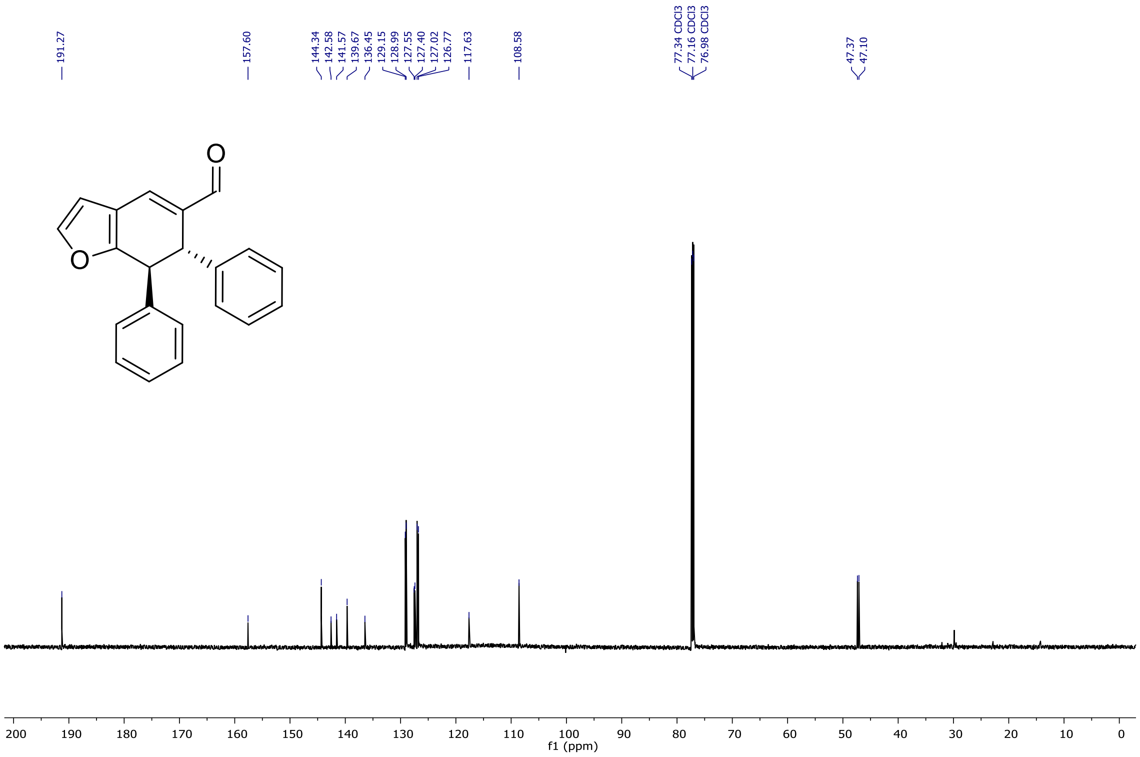

(6*S*,7*S*)-6-(4-Nitrophenyl)-7-phenyl-6,7-dihydrobenzofuran-5-carbaldehyde 3b

^{1}H NMR (700 MHz, $CDCl_3$)

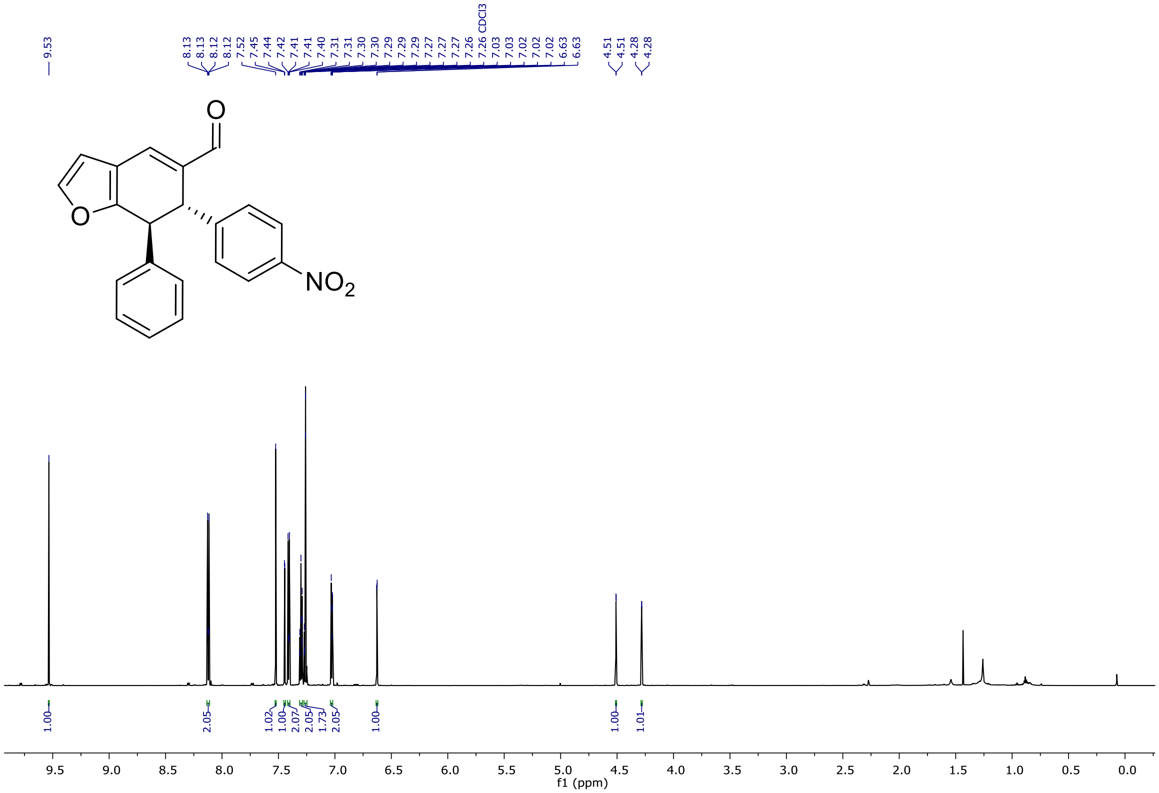

^{13}C NMR (176 MHz, $CDCl_3$)

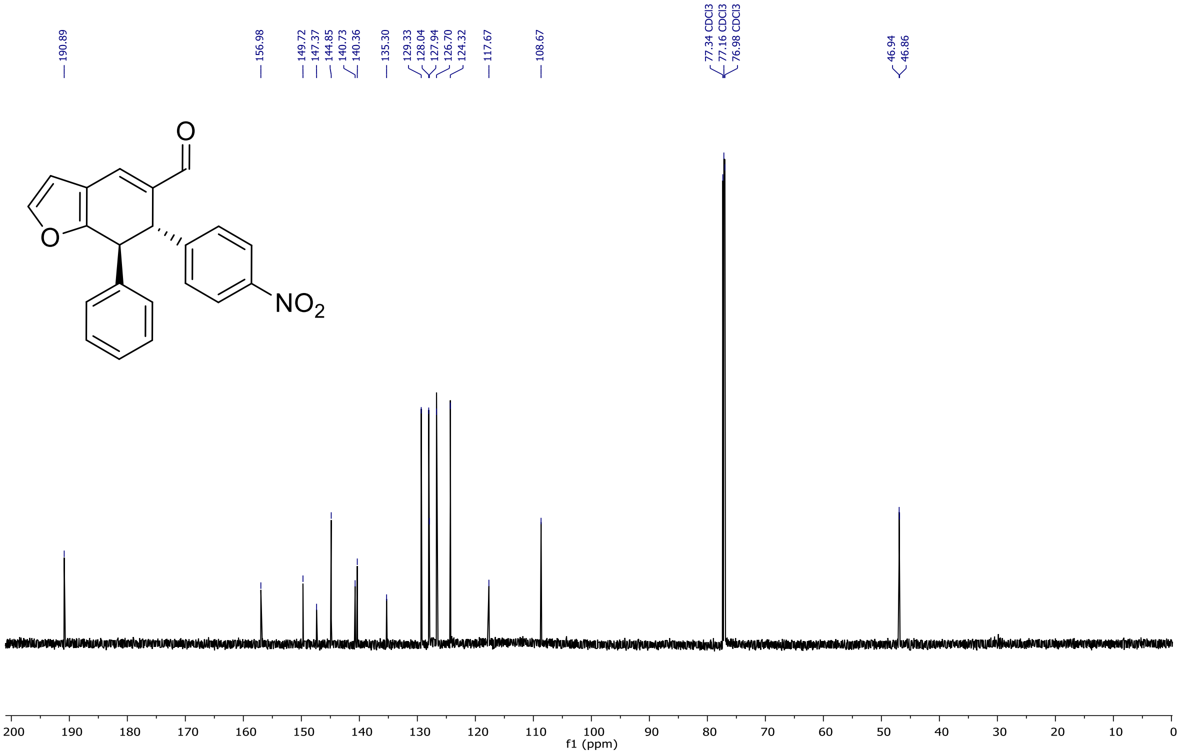

(6*S*,7*S*)-6-(4-Chlorophenyl)-7-phenyl-6,7-dihydrobenzofuran-5-carbaldehyde 3c

^{1}H NMR (700 MHz, $CDCl_3$)

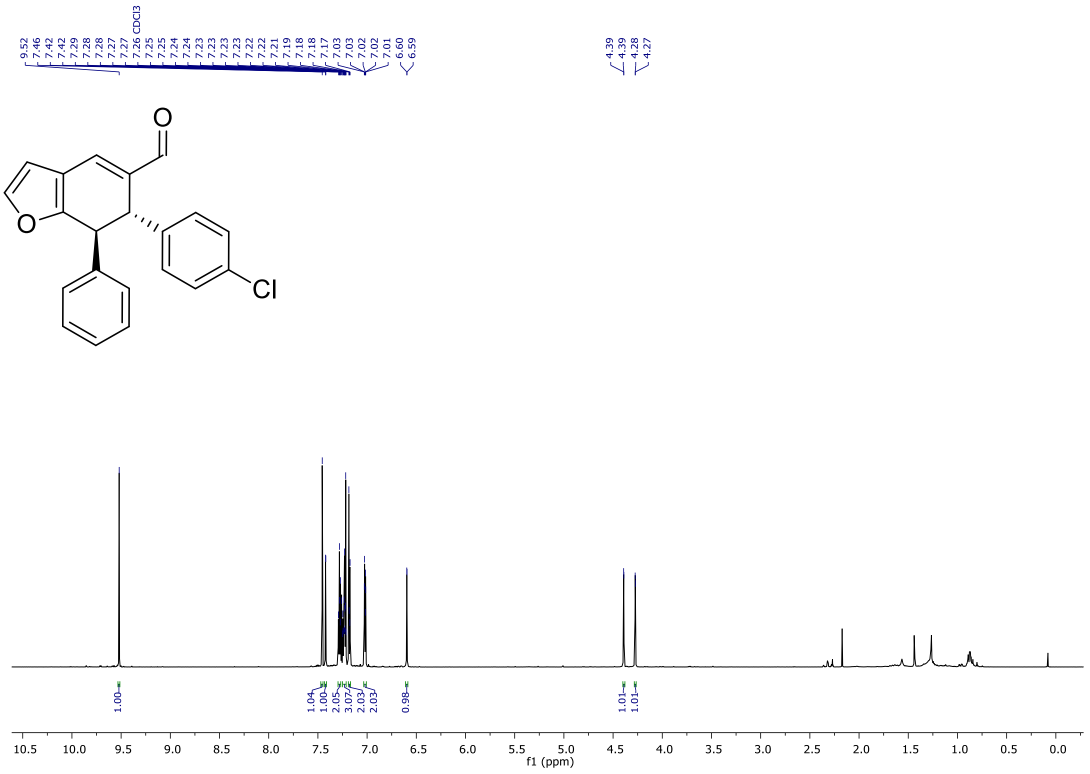

^{13}C NMR (176 MHz, $CDCl_3$)

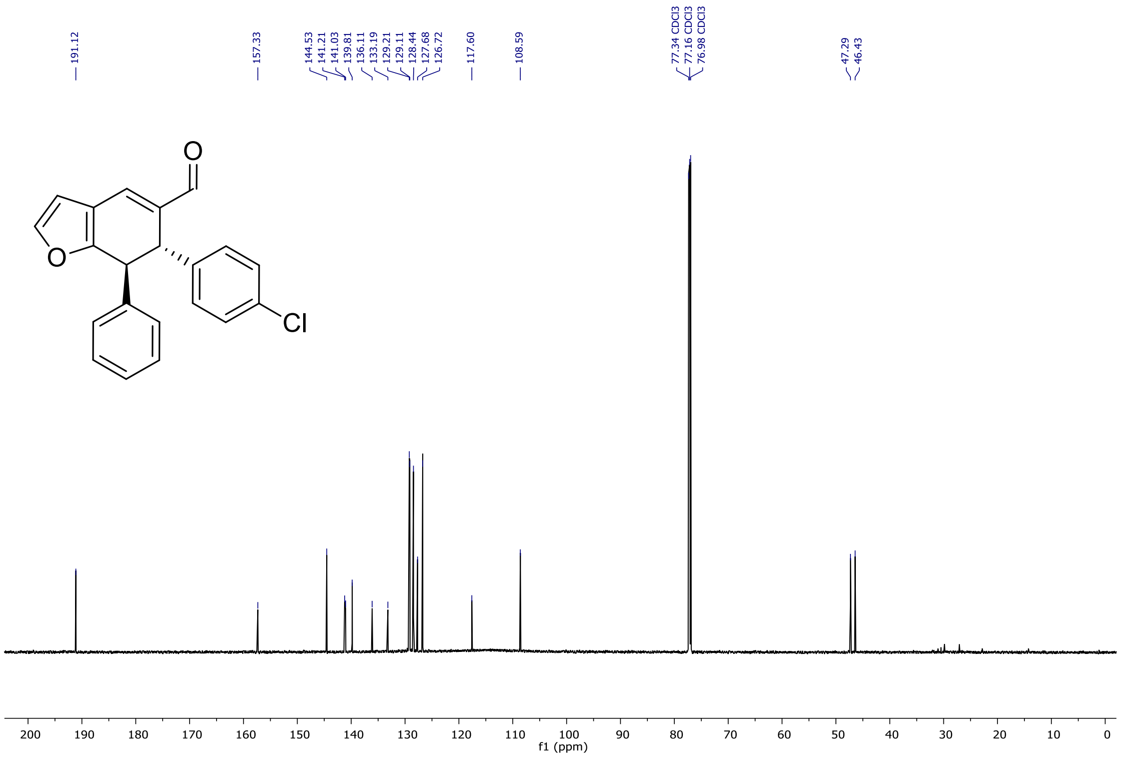

(6*S*,7*S*)-7-Phenyl-6-(p-tolyl)-6,7-dihydrobenzofuran-5-carbaldehyde 3d

^{1}H NMR (700 MHz, $CDCl_3$)

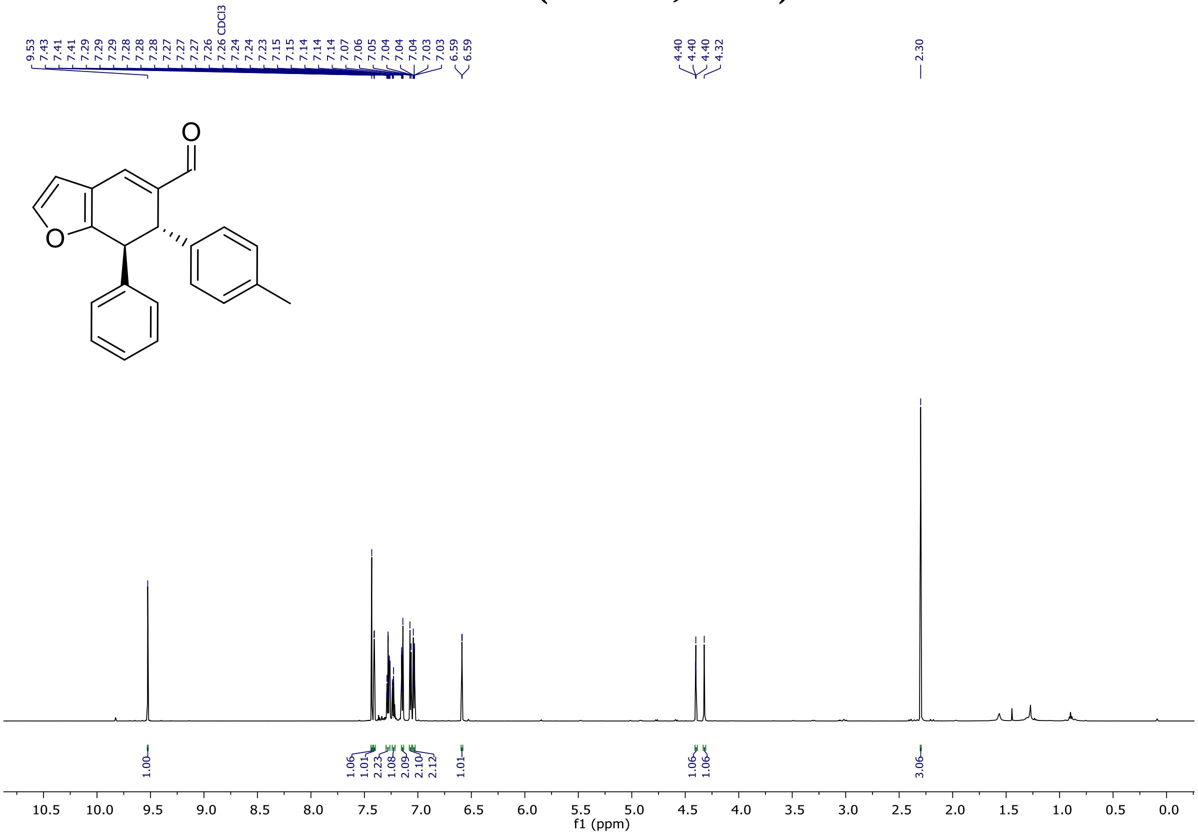

^{13}C NMR (176 MHz, $CDCl_3$)

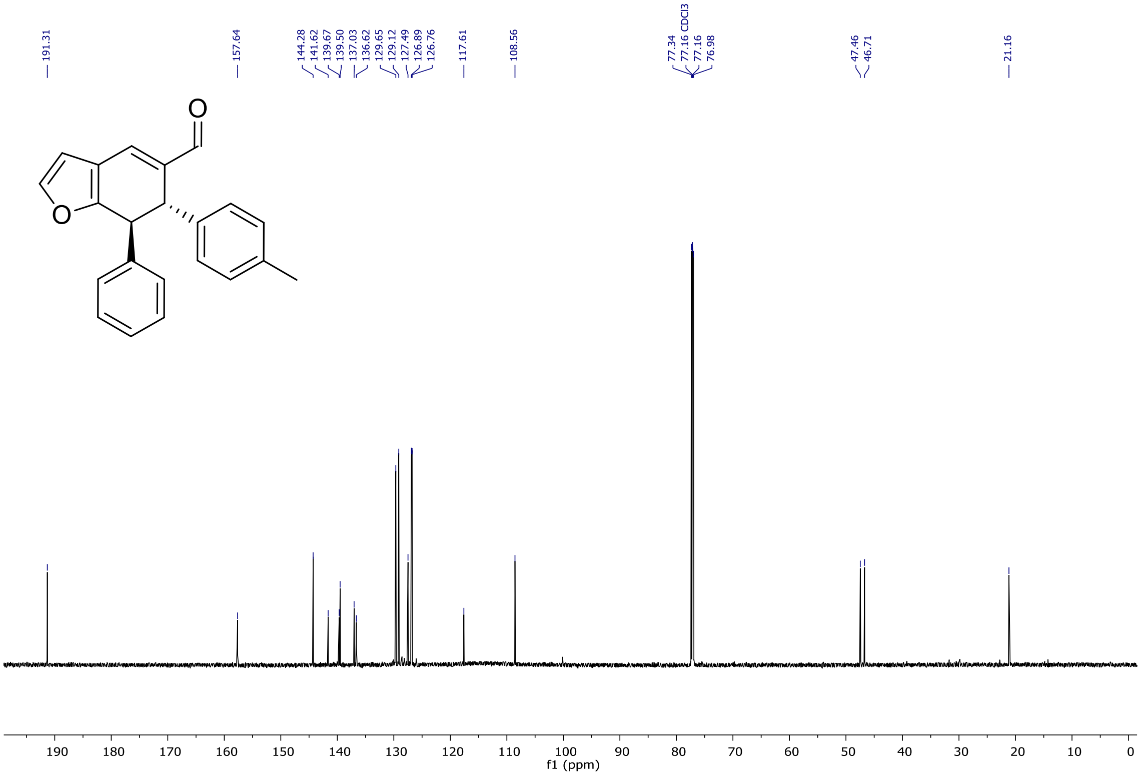

(6*S*,7*S*)-6-(4-Methoxyphenyl)-7-phenyl-6,7-dihydrobenzofuran-5-carbaldehyde 3e

^{1}H NMR (700 MHz, CDCl$_3$)

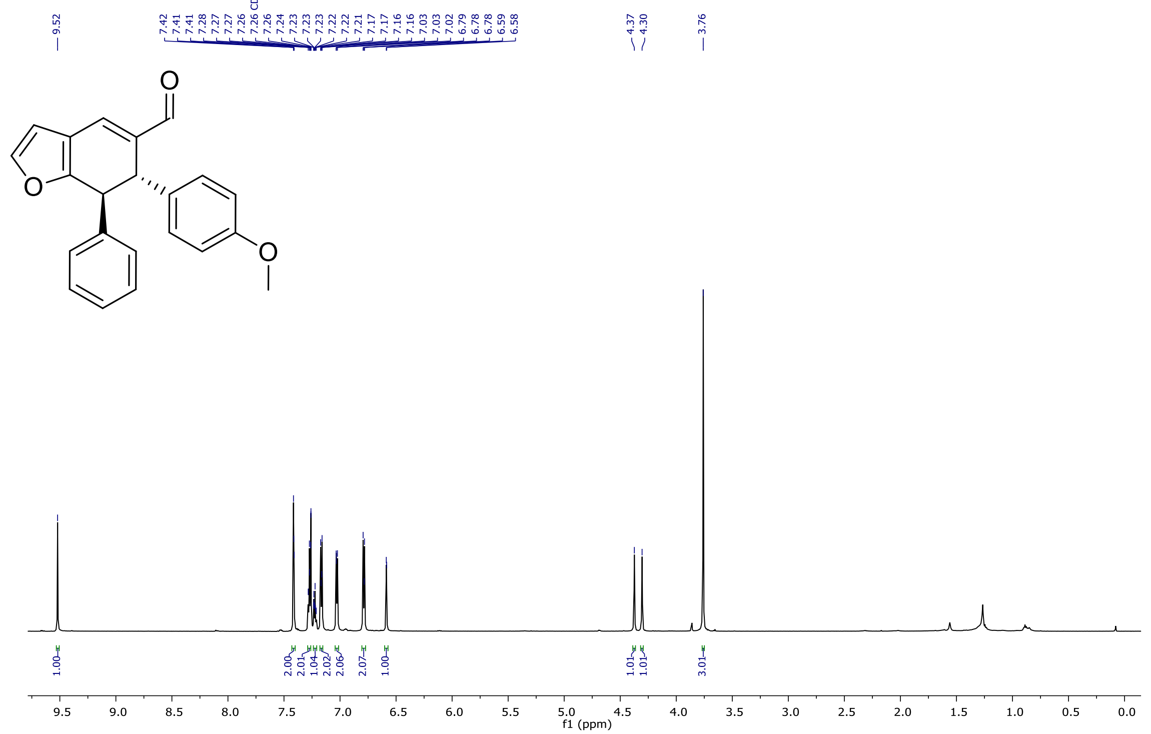

^{13}C NMR (176 MHz, CDCl$_3$)

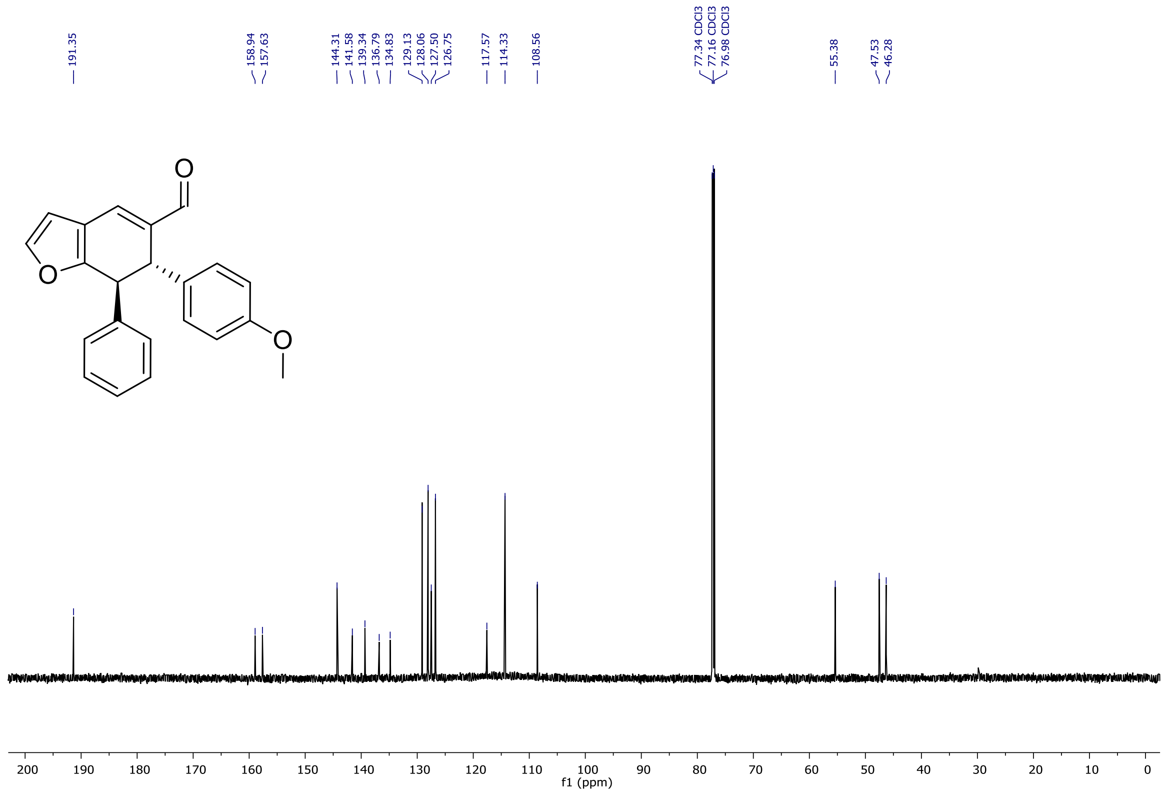

(6*S*,7*S*)-6-(3-Methoxyphenyl)-7-phenyl-6,7-dihydrobenzofuran-5-carbaldehyde 3f

^{1}H NMR (700 MHz, $CDCl_3$)

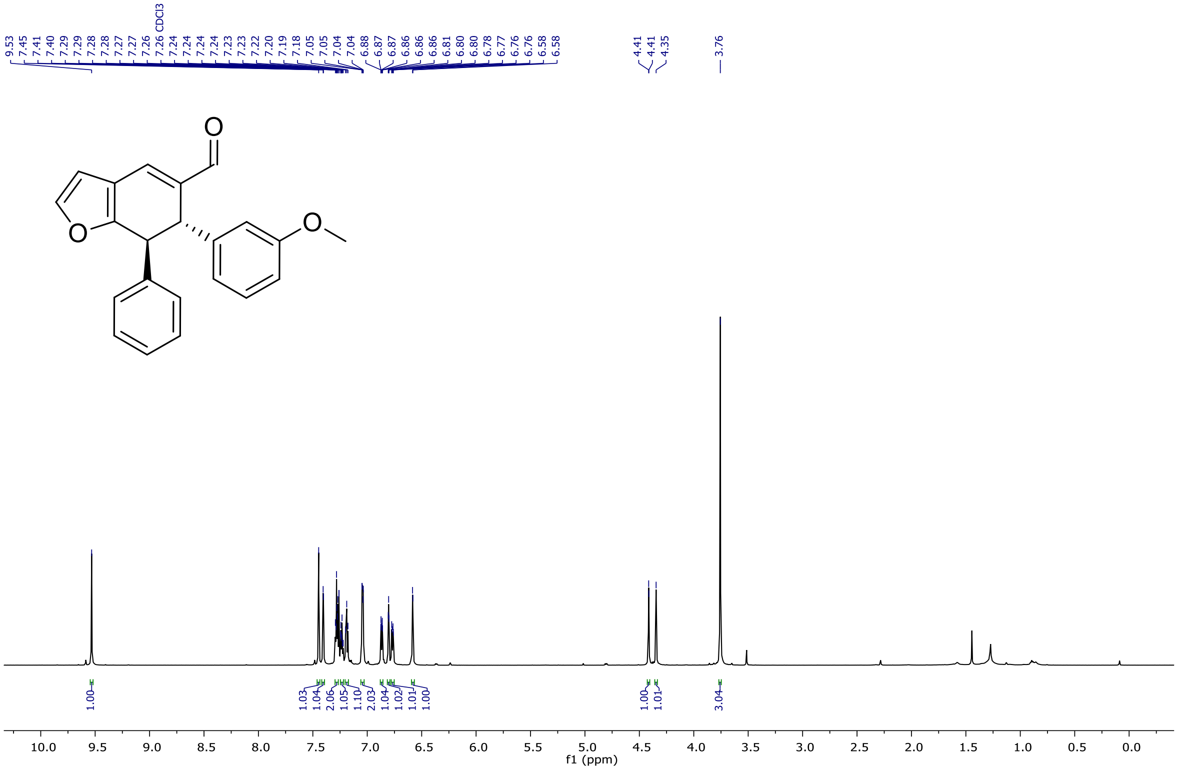

^{13}C NMR (176 MHz, $CDCl_3$)

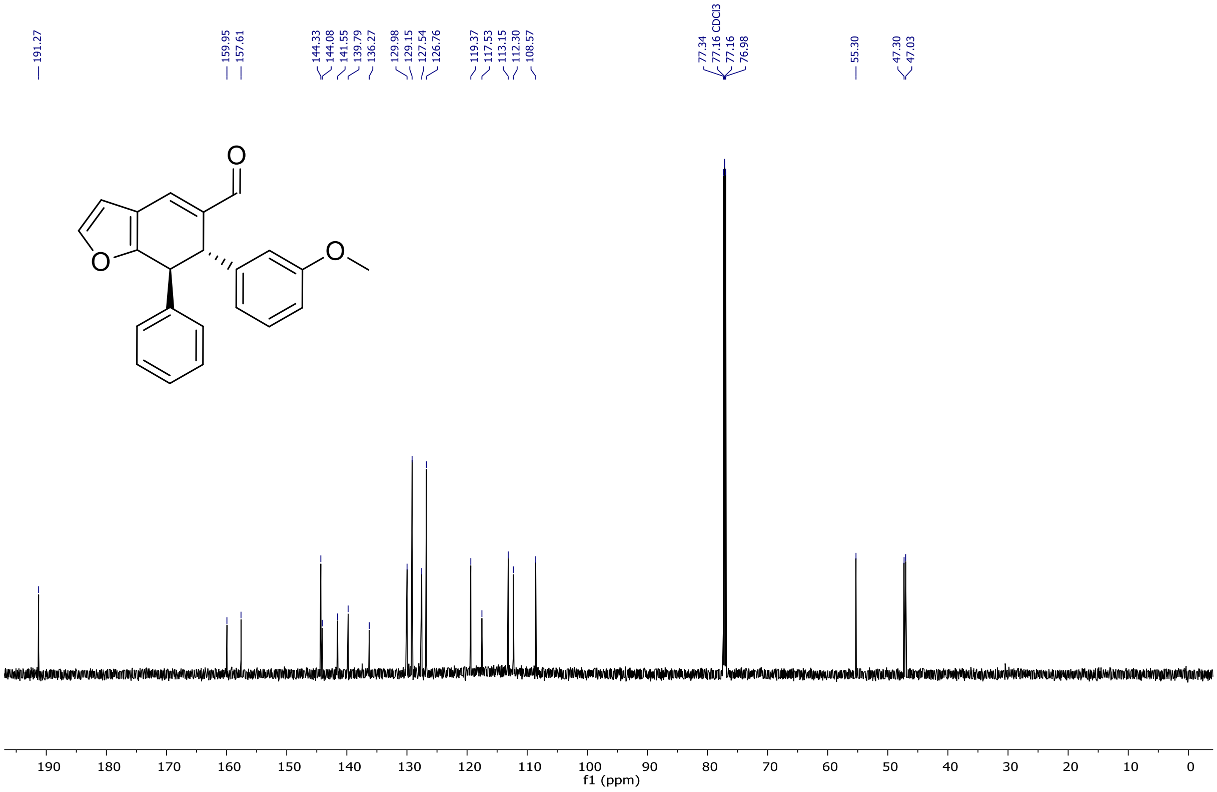

(6*S*,7*S*)-6-(2-Methoxyphenyl)-7-phenyl-6,7-dihydrobenzofuran-5-carbaldehyde 3g

^{1}H NMR (700 MHz, $CDCl_3$)

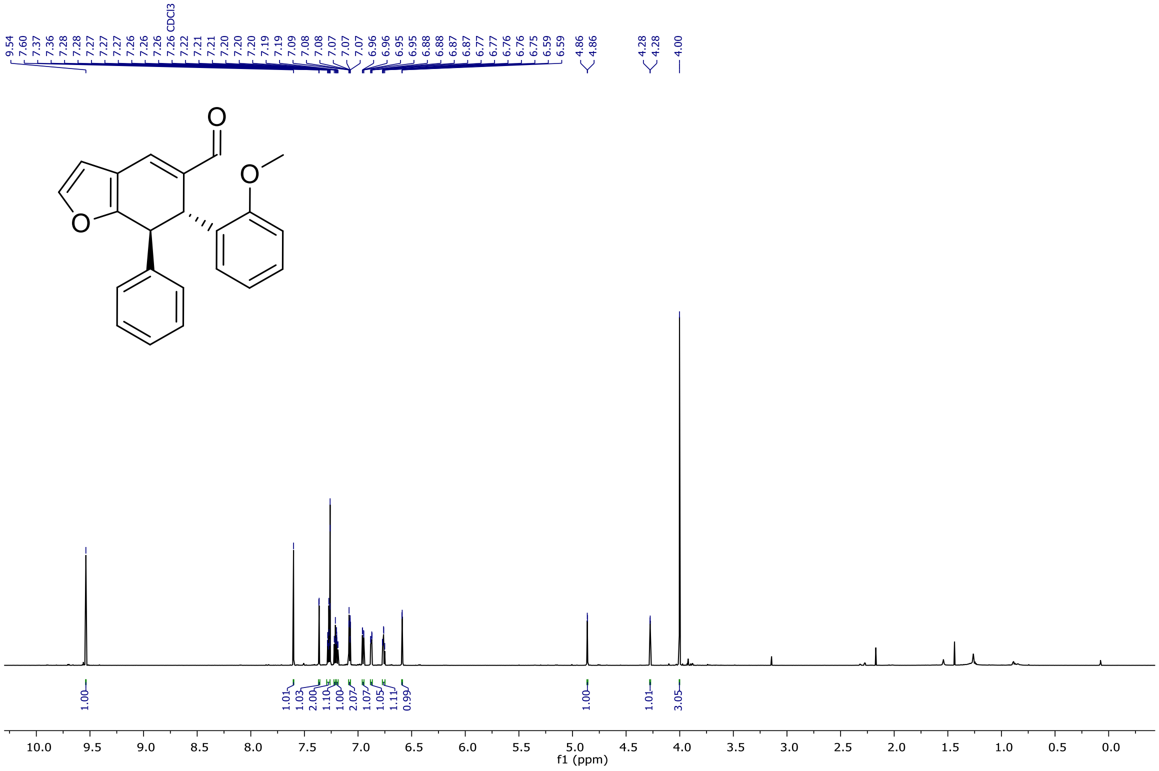

^{13}C NMR (176 MHz, $CDCl_3$)

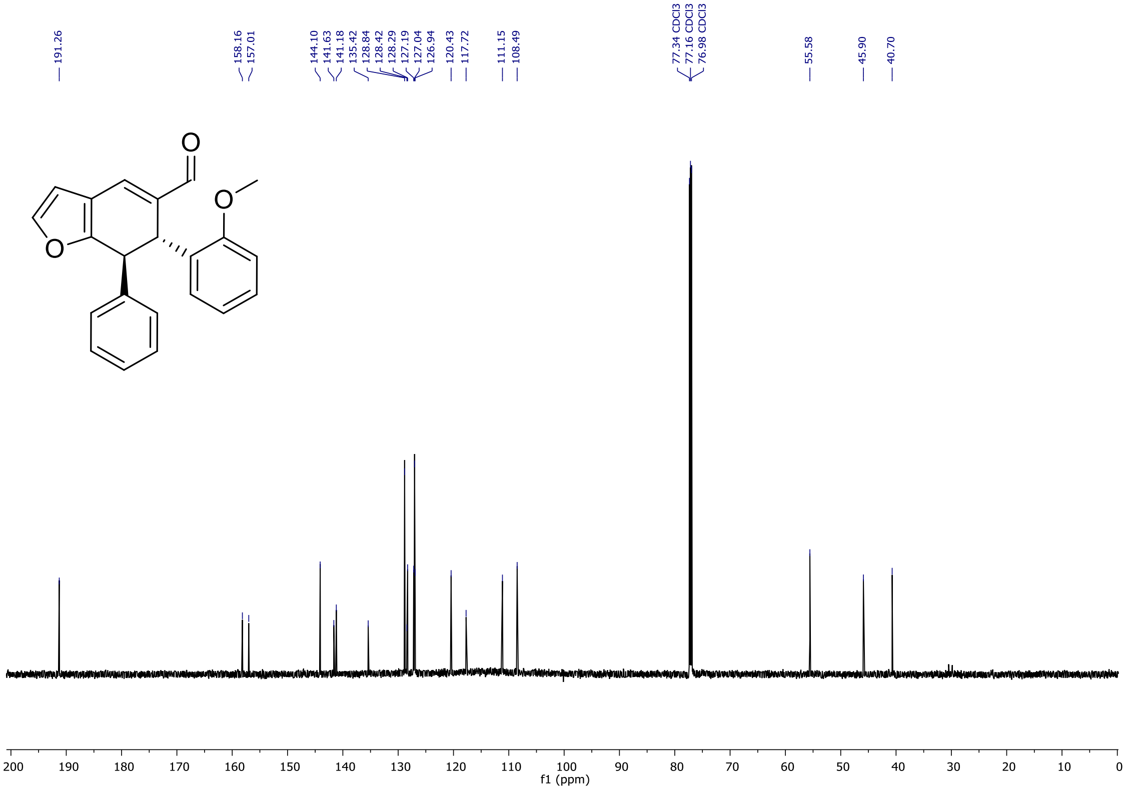

(6*R*,7*S*)-6-(2,4-Dichlorophenyl)-7-phenyl-6,7-dihydrobenzofuran-5-carbaldehyde 3h

^{1}H NMR (700 MHz, $CDCl_3$)

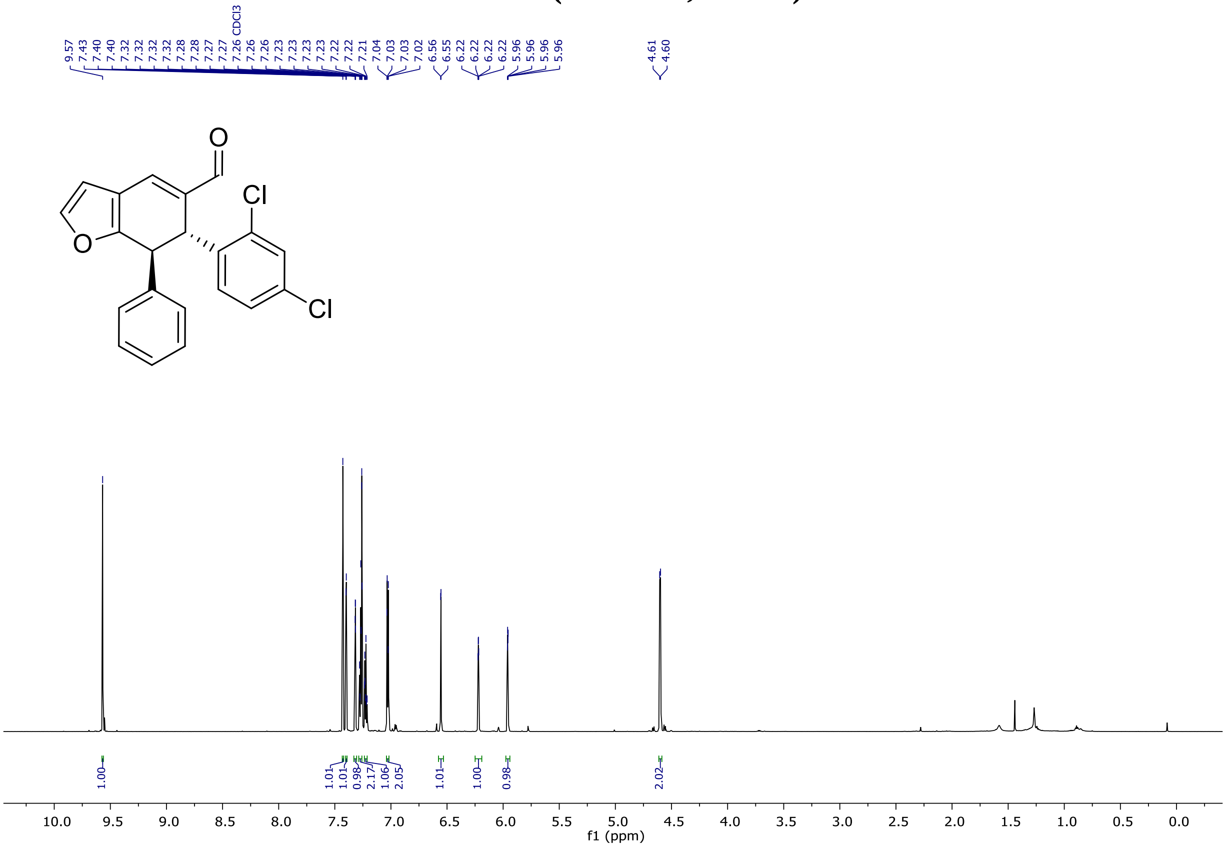

^{13}C NMR (176 MHz, $CDCl_3$)

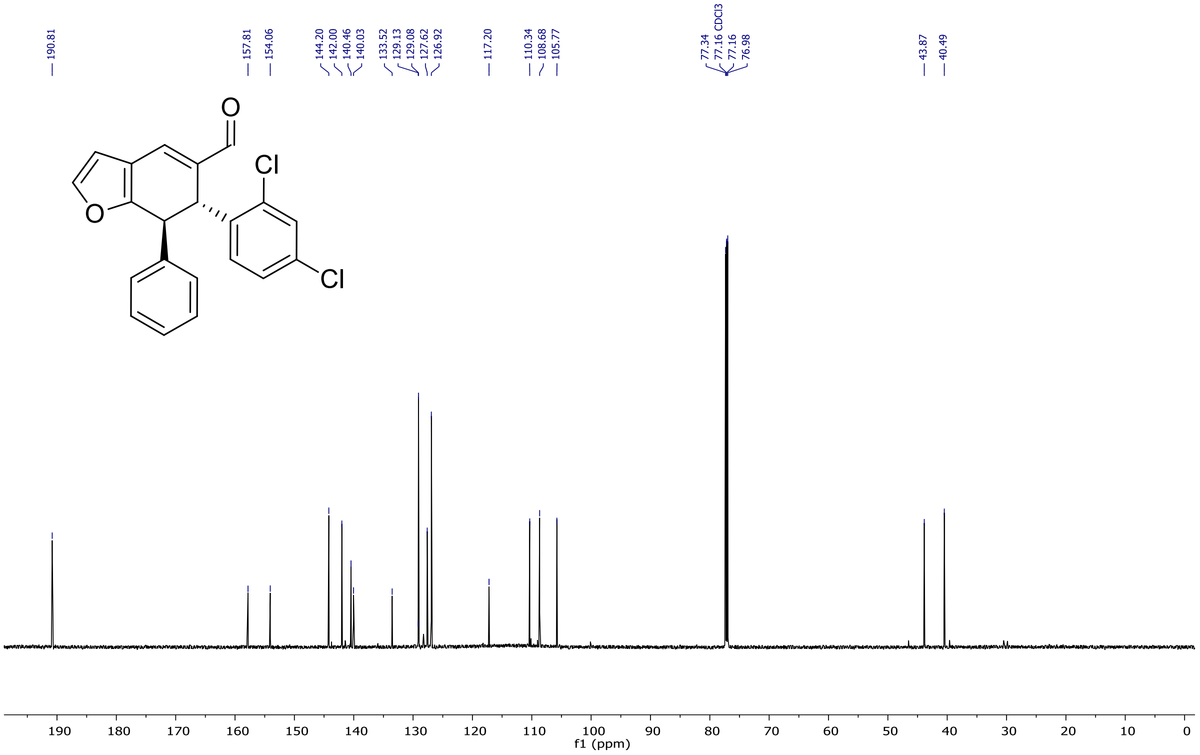

(6*R*,7*S*)-6-(Furan-2-yl)-7-phenyl-6,7-dihydrobenzofuran-5-carbaldehyde 3i

^{1}H NMR (700 MHz, CDCl$_3$)

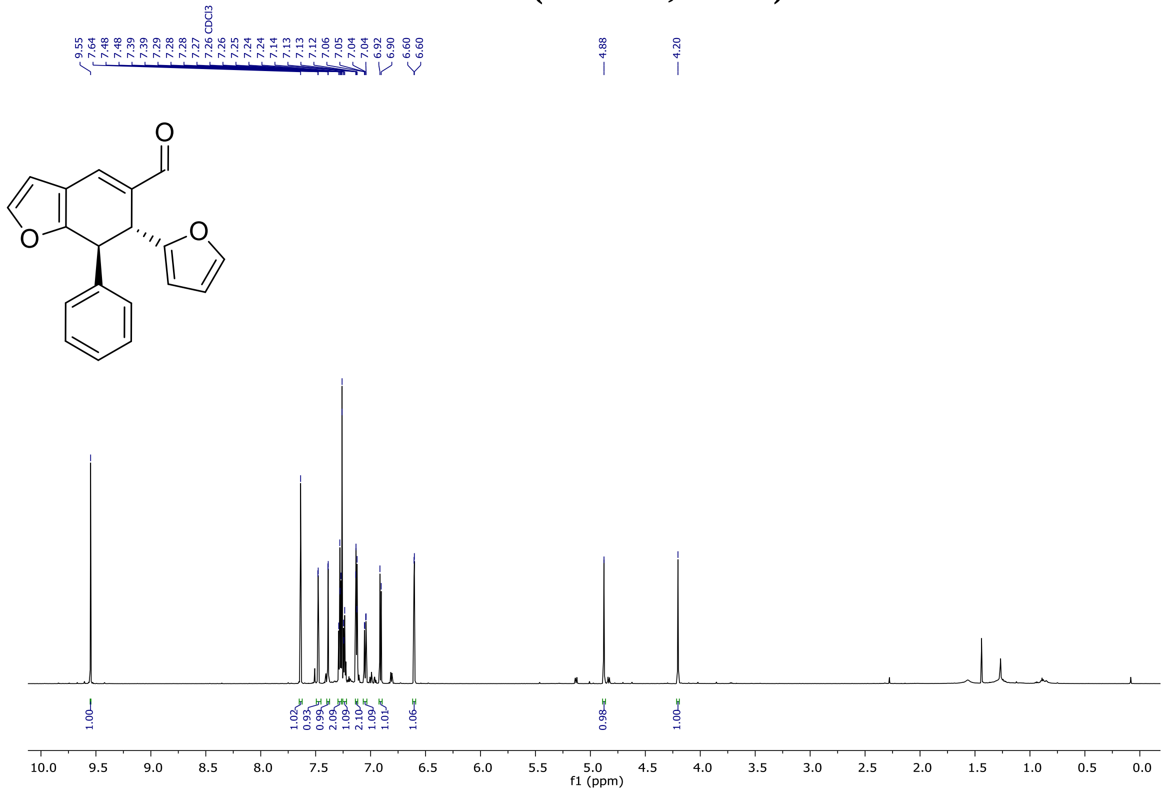

^{13}C NMR (176 MHz, CDCl$_3$)

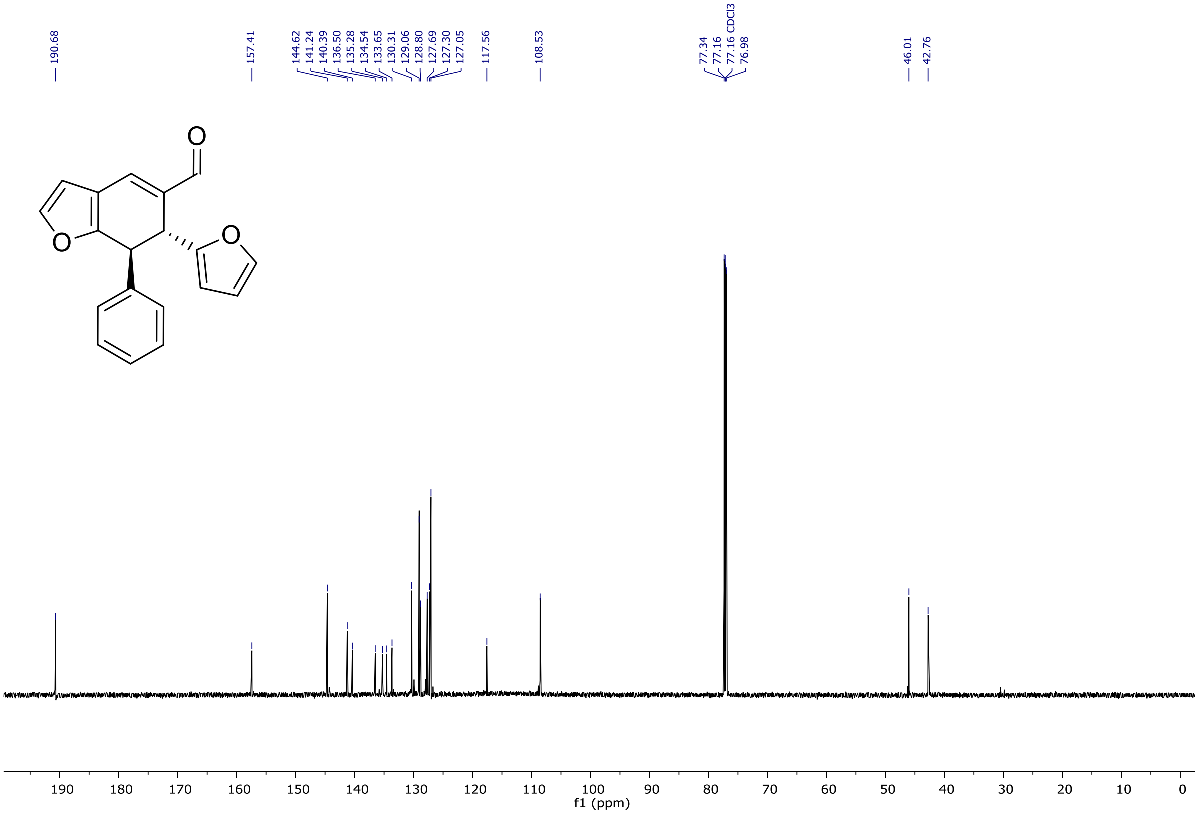

(6*S*,7*S*)-7-(4-Fluorophenyl)-6-phenyl-6,7-dihydrobenzofuran-5-carbaldehyde 3j

^{1}H NMR (700 MHz, $CDCl_3$)

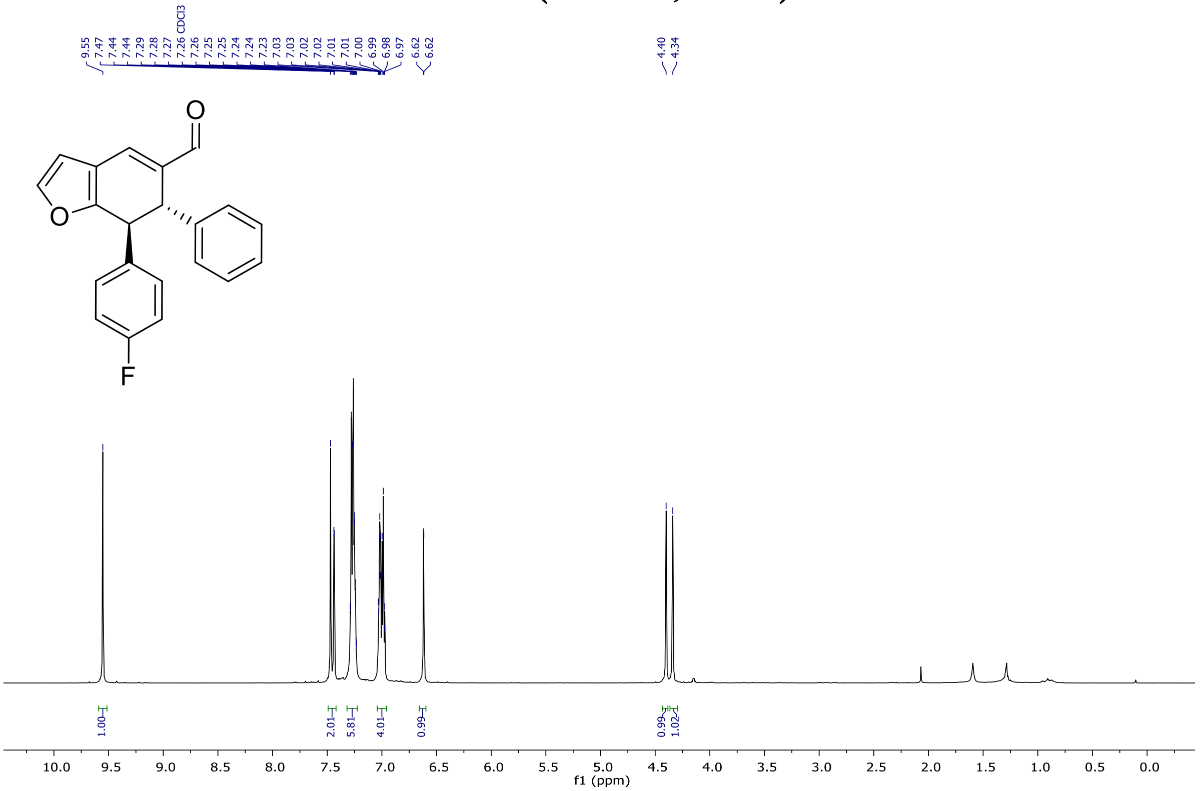

^{13}C NMR (176 MHz, $CDCl_3$)

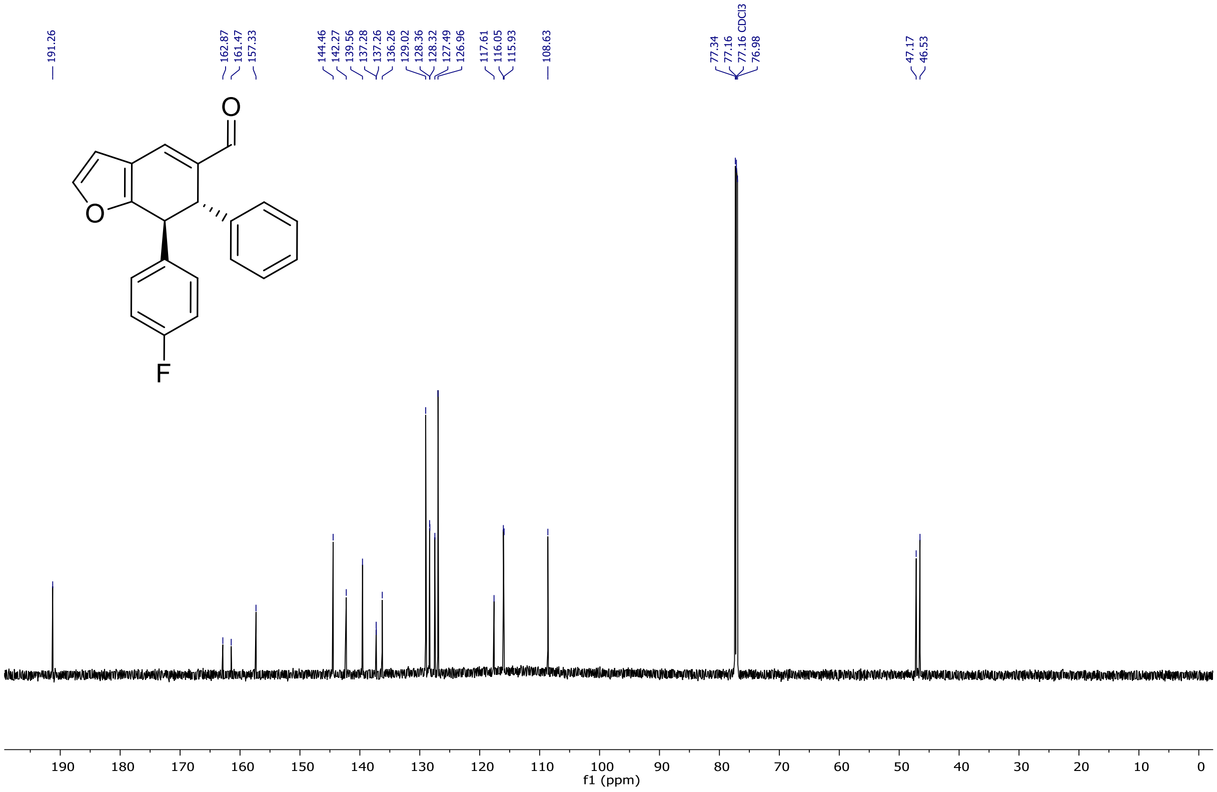

(6*S*,7*S*)-6-Phenyl-7-(p-tolyl)-6,7-dihydrobenzofuran-5-carbaldehyde 3k

^{1}H NMR (700 MHz, CDCl$_3$)

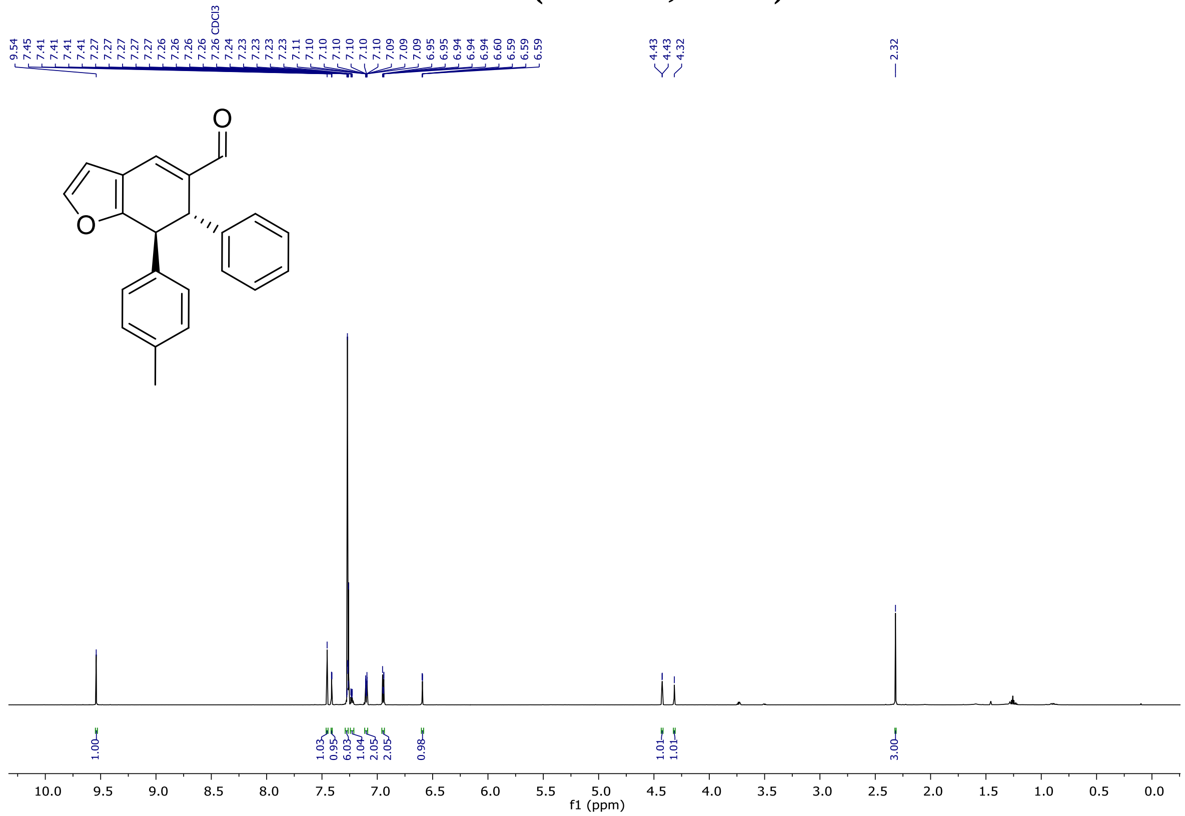

^{13}C NMR (176 MHz, CDCl$_3$)

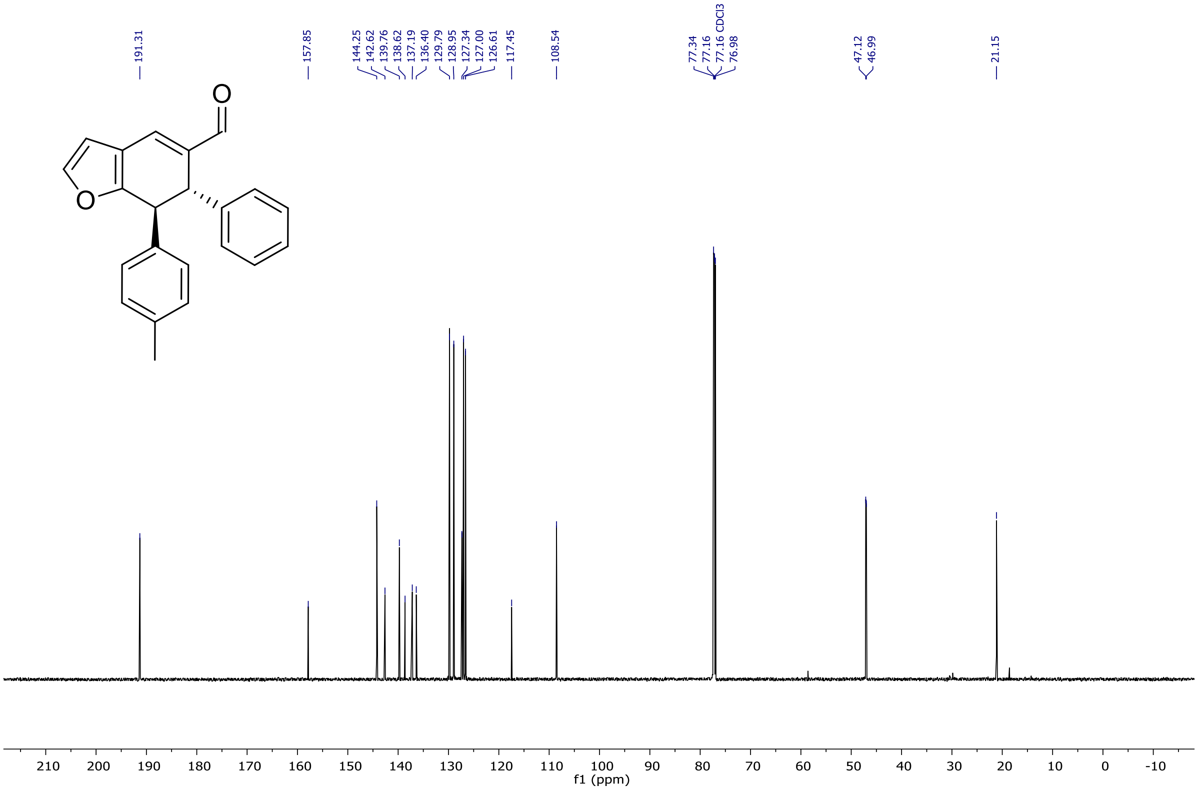

(6*S*,7*S*)-7-(3-Methoxyphenyl)-6-phenyl-6,7-dihydrobenzofuran-5-carbaldehyde 3l

^{1}H NMR (700 MHz, CDCl$_3$)

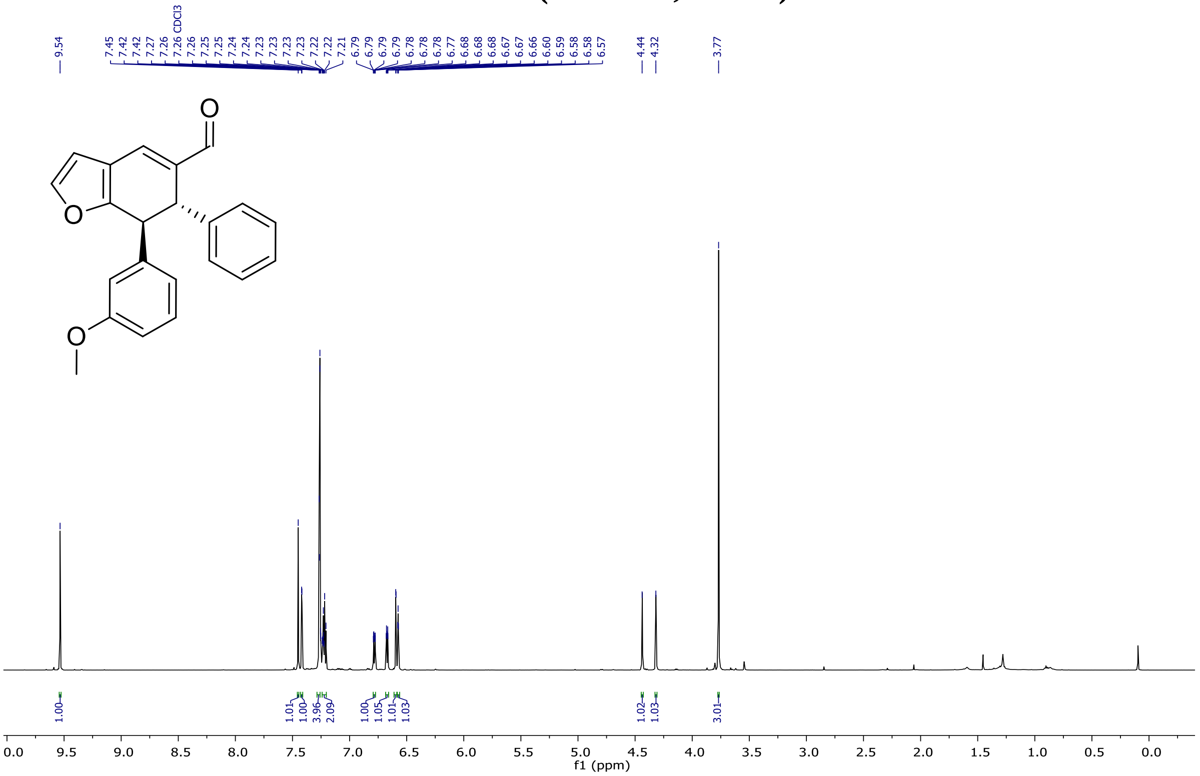

^{13}C NMR (176 MHz, CDCl$_3$)

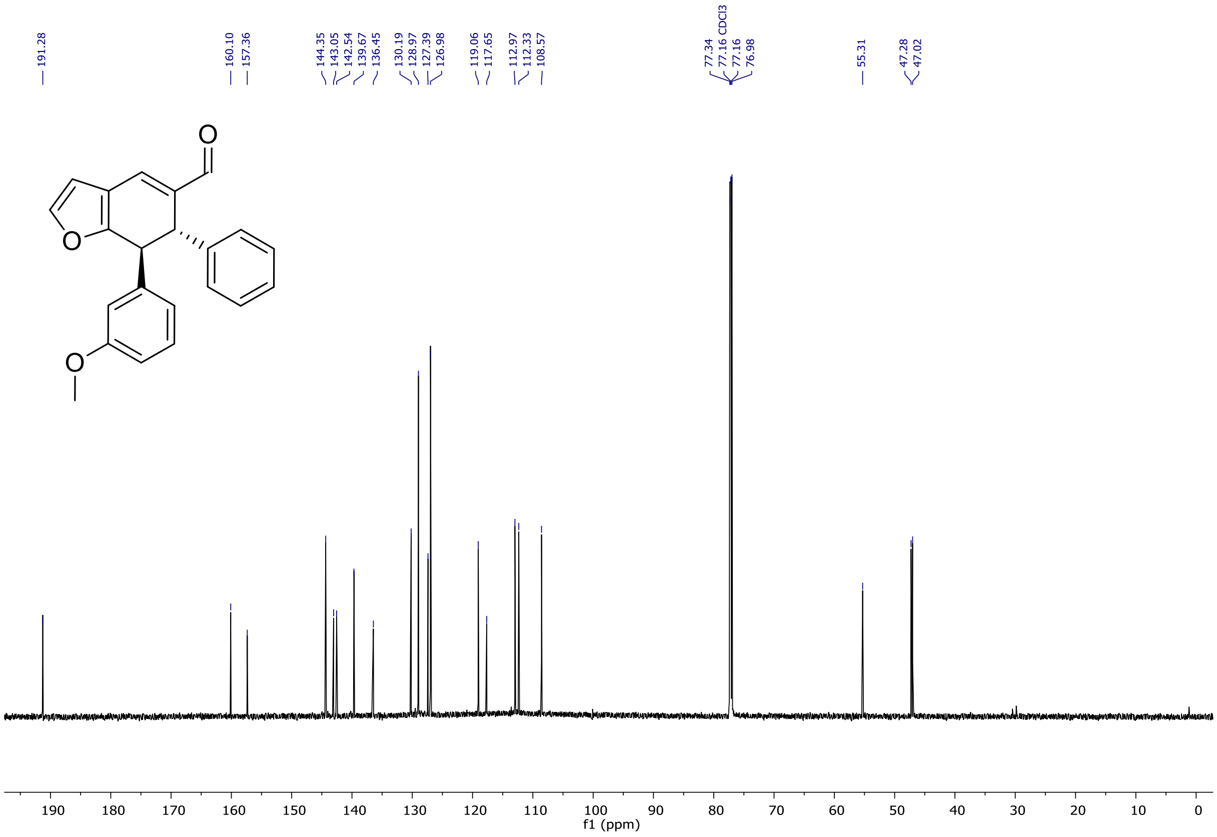

(6*S*,7*S*)-6-Phenyl-7-(o-tolyl)-6,7-dihydrobenzofuran-5-carbaldehyde 3m

^{1}H NMR (700 MHz, CDCl$_3$)

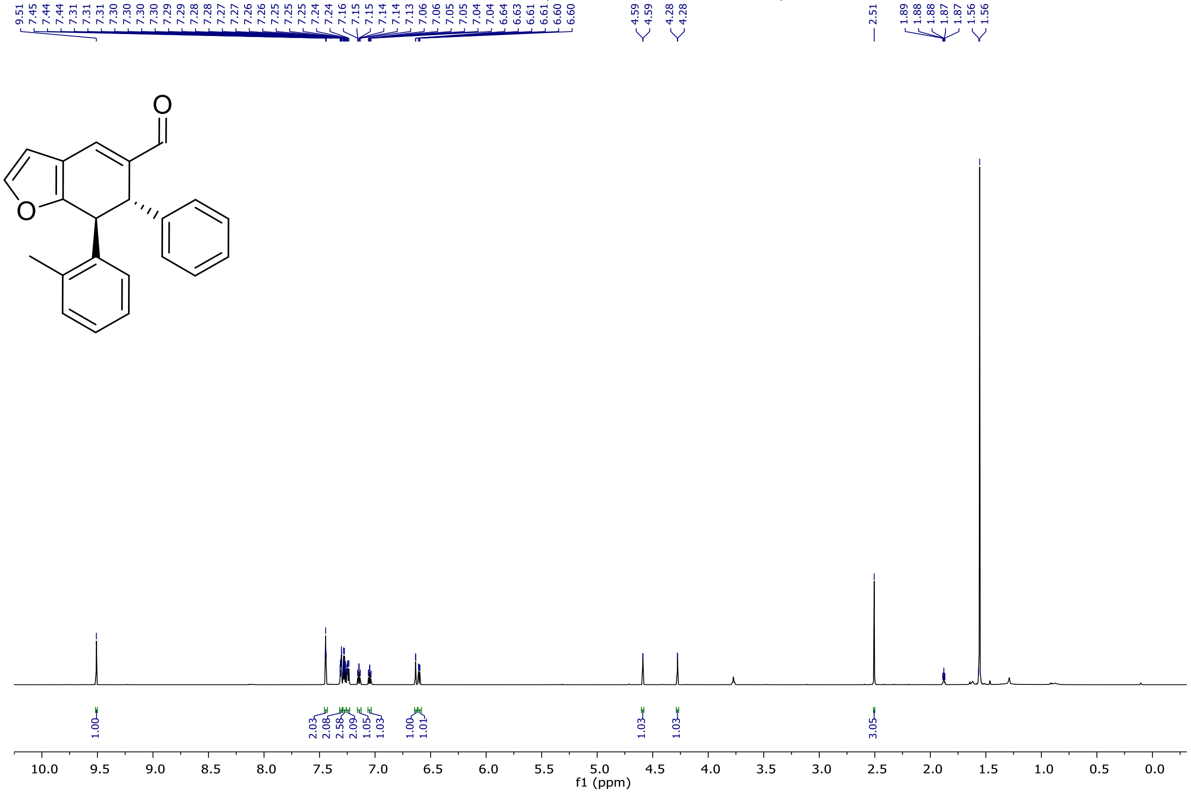

^{13}C NMR (176 MHz, CDCl$_3$)

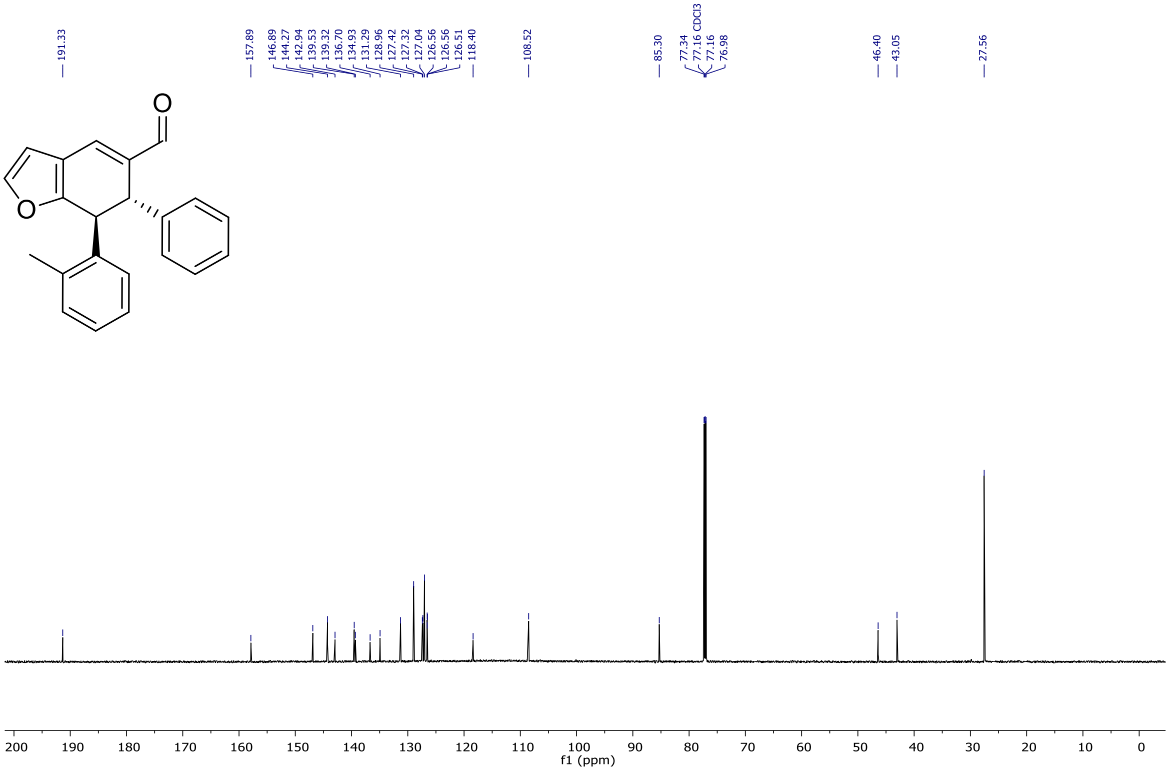

(6*S*,7*R*)-6-Phenyl-7-vinyl-6,7-dihydrobenzofuran-5-carbaldehyde 3n

^{1}H NMR (700 MHz, $CDCl_3$)

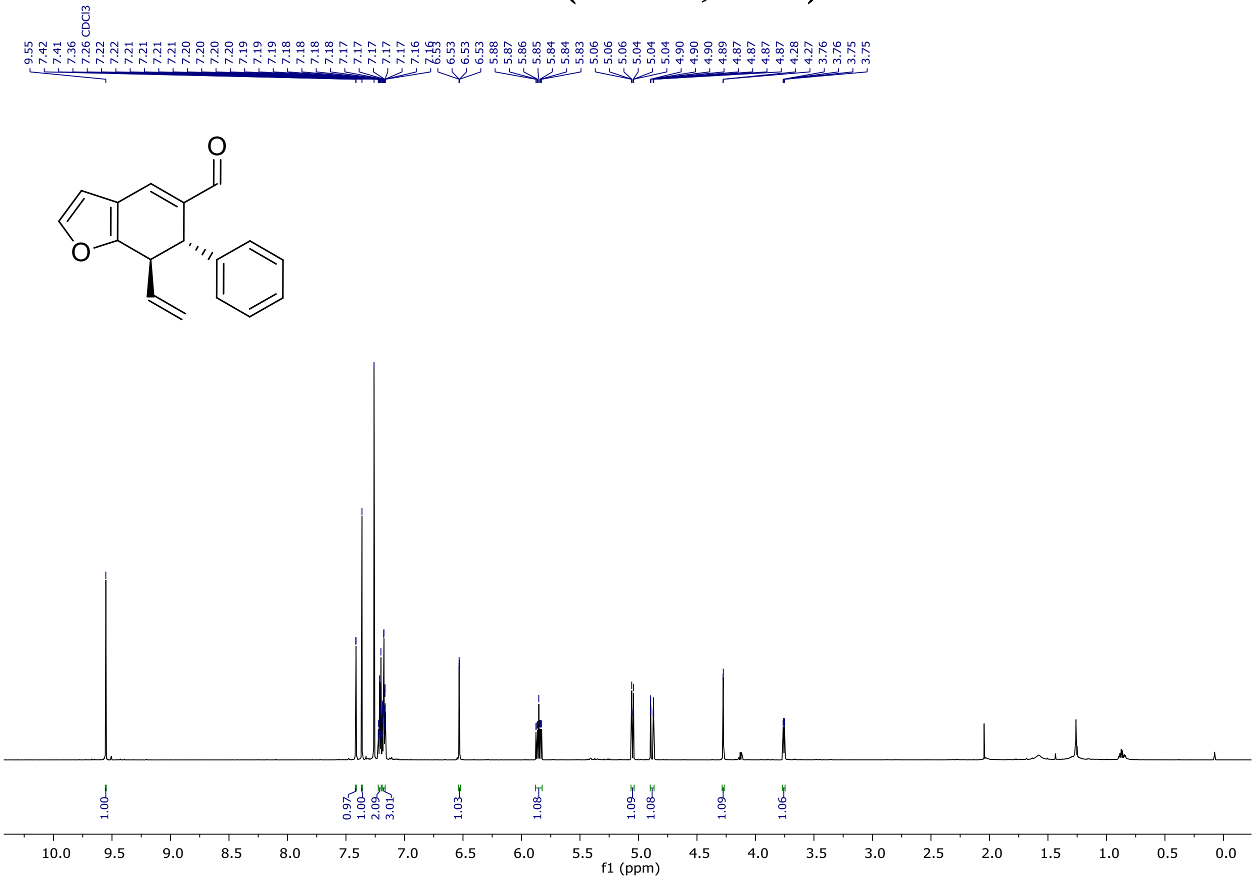

^{13}C NMR (176 MHz, $CDCl_3$)

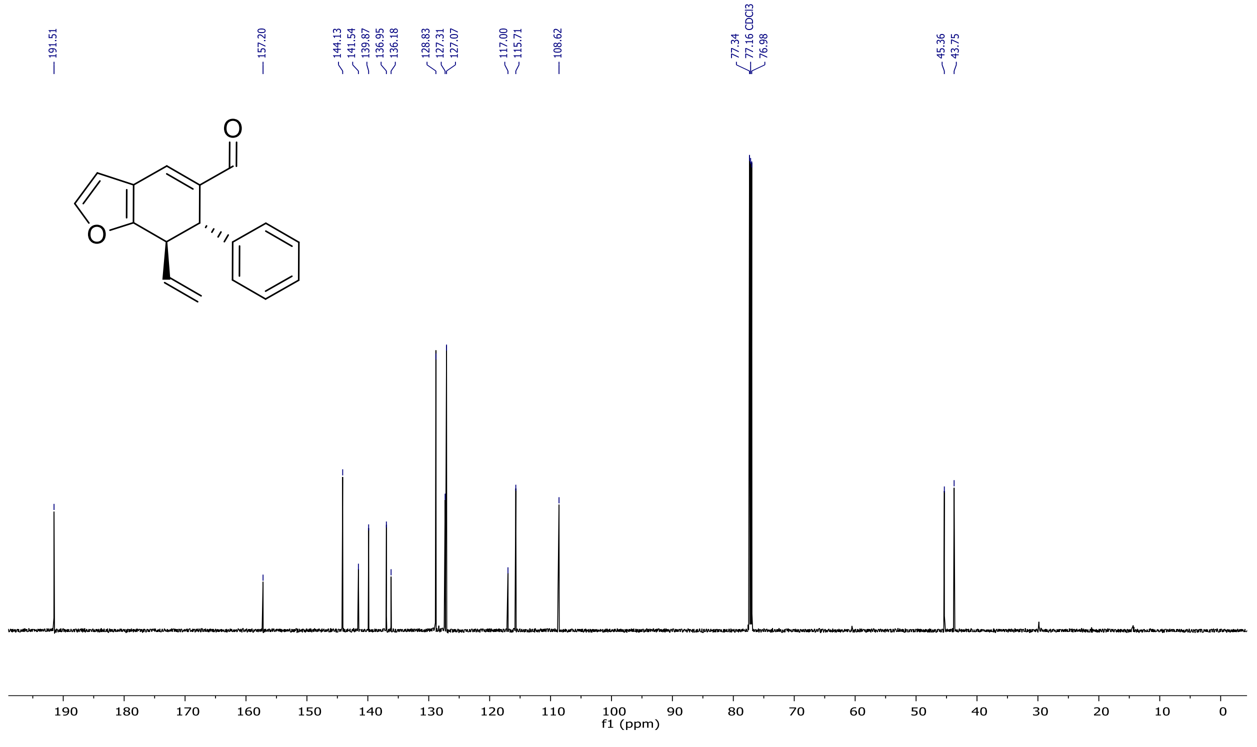

(3*S*,4*S*)-3,4-Diphenyl-3,4-dihydrodibenzo[b,d]furan-2-carbaldehyde 3o

^{1}H NMR (700 MHz, $CDCl_3$)

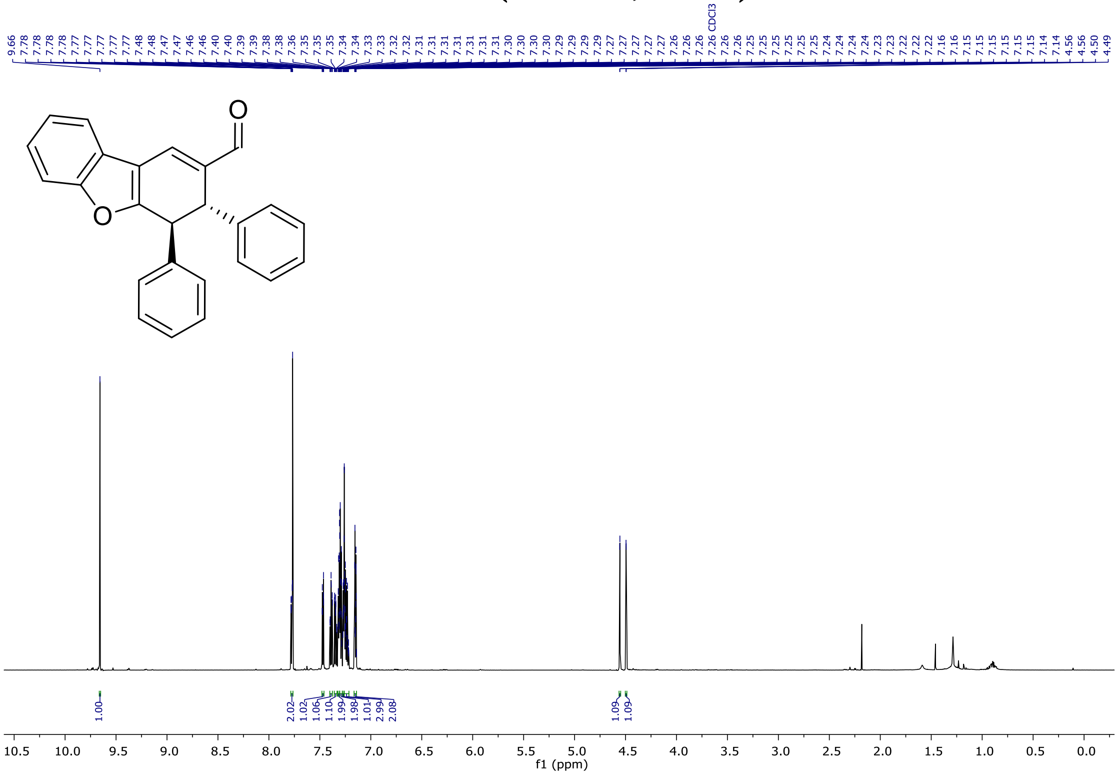

^{13}C NMR (176 MHz, $CDCl_3$)

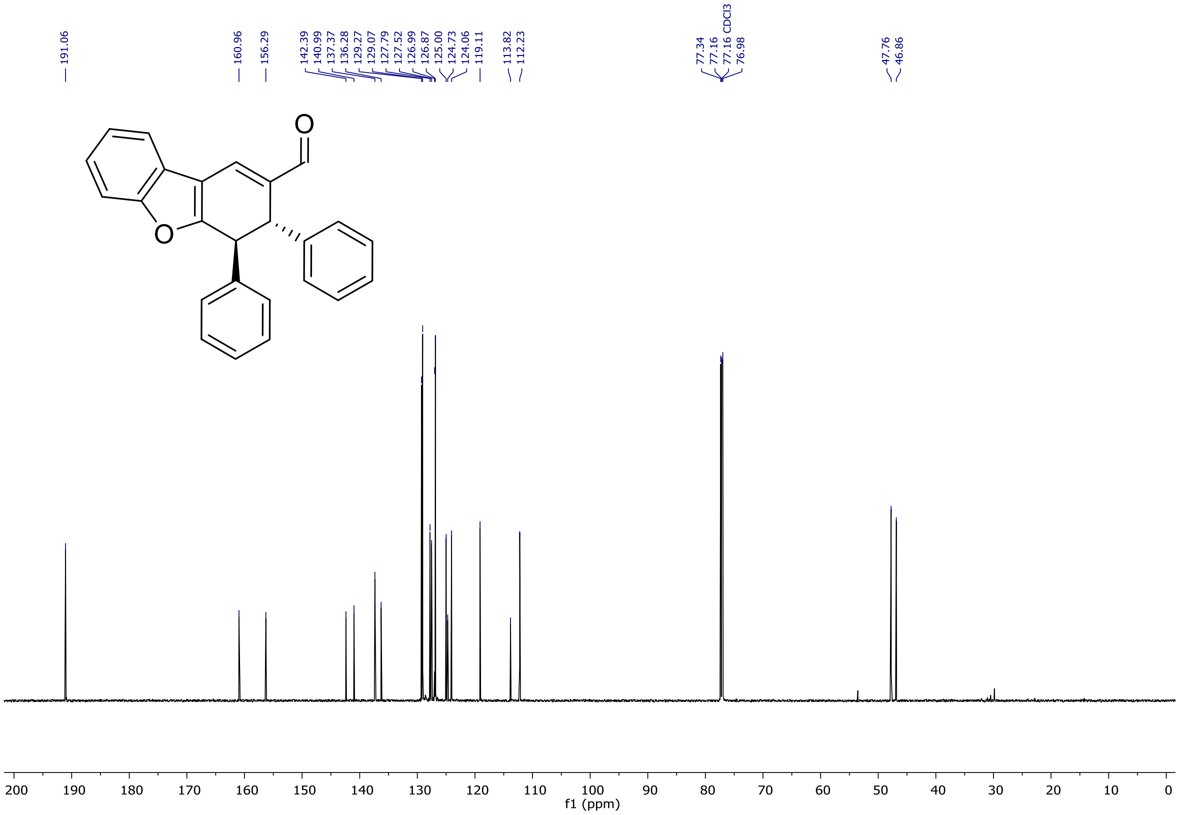

6,7-Diphenylbenzofuran-5-carbaldehyde 9

^{1}H NMR (700 MHz, $CDCl_3$)

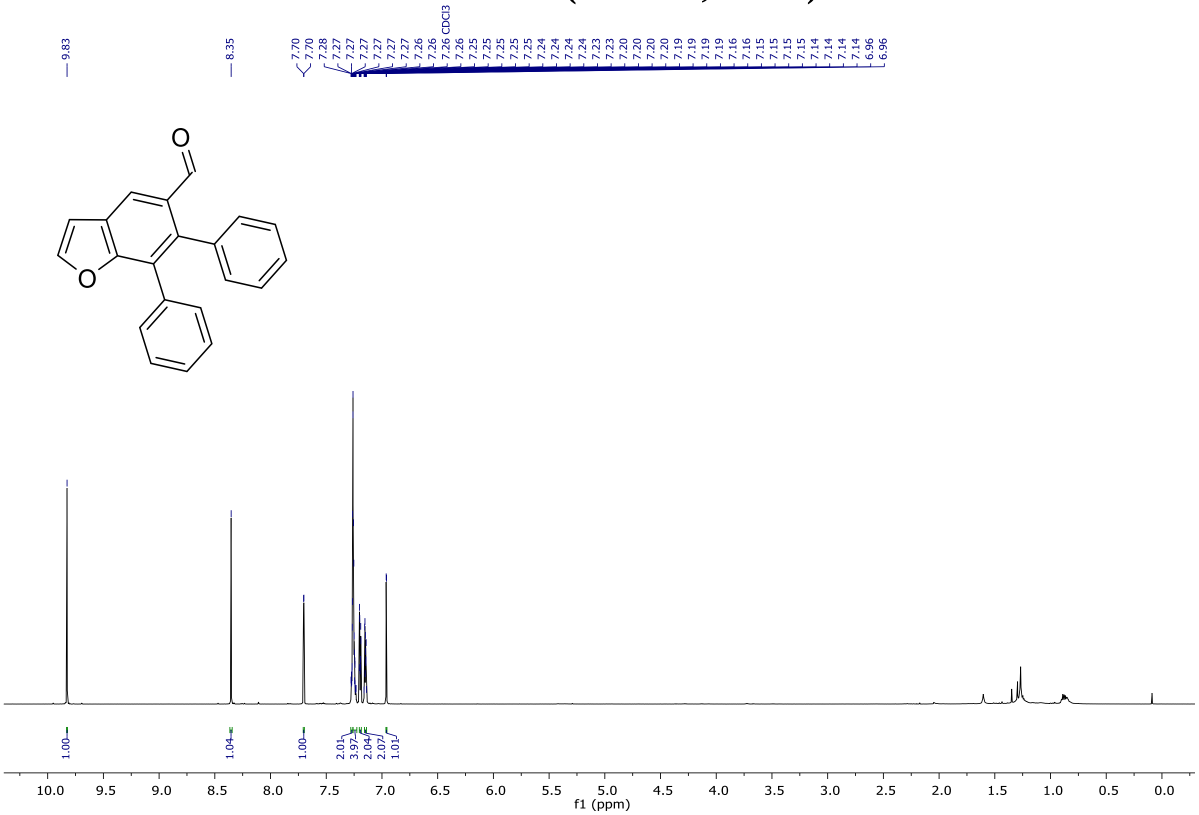

^{13}C NMR (176 MHz, $CDCl_3$)

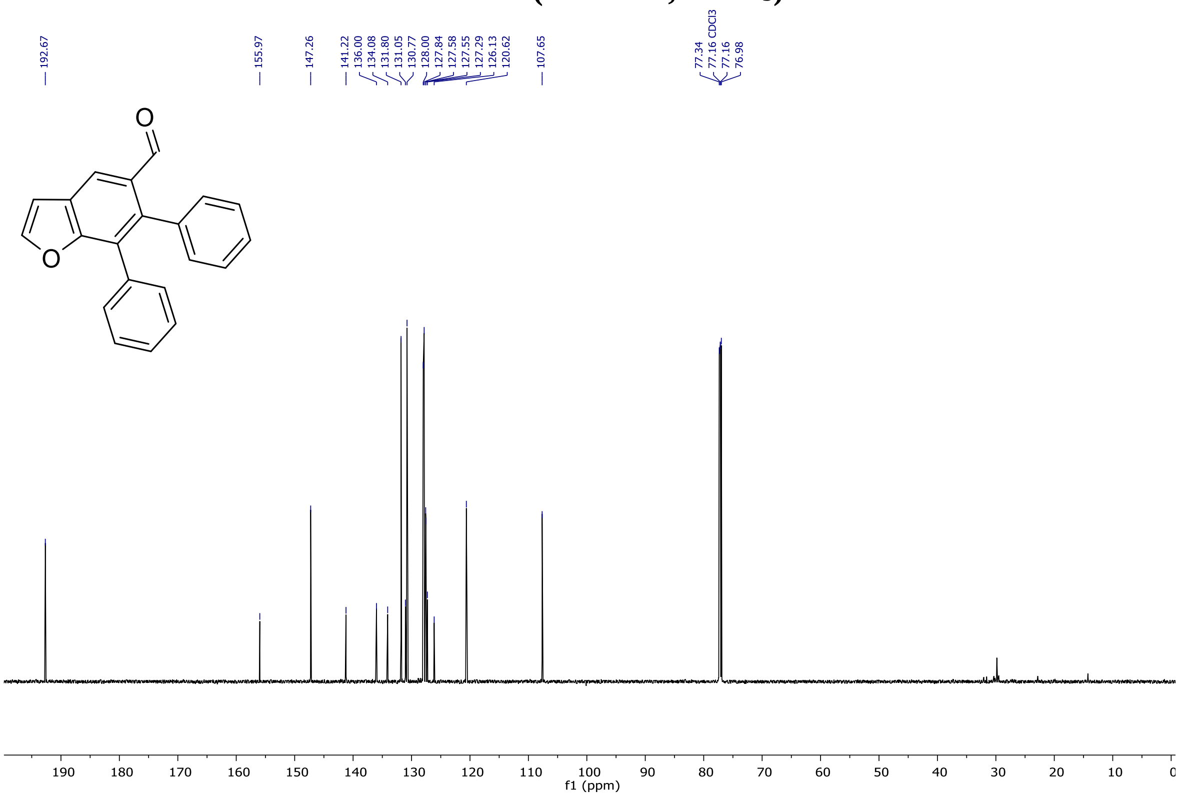

((6*S*,7*S*)-6,7-Diphenyl-6,7-dihydrobenzofuran-5-yl)methanol 7

^{1}H NMR (700 MHz, $CDCl_3$)

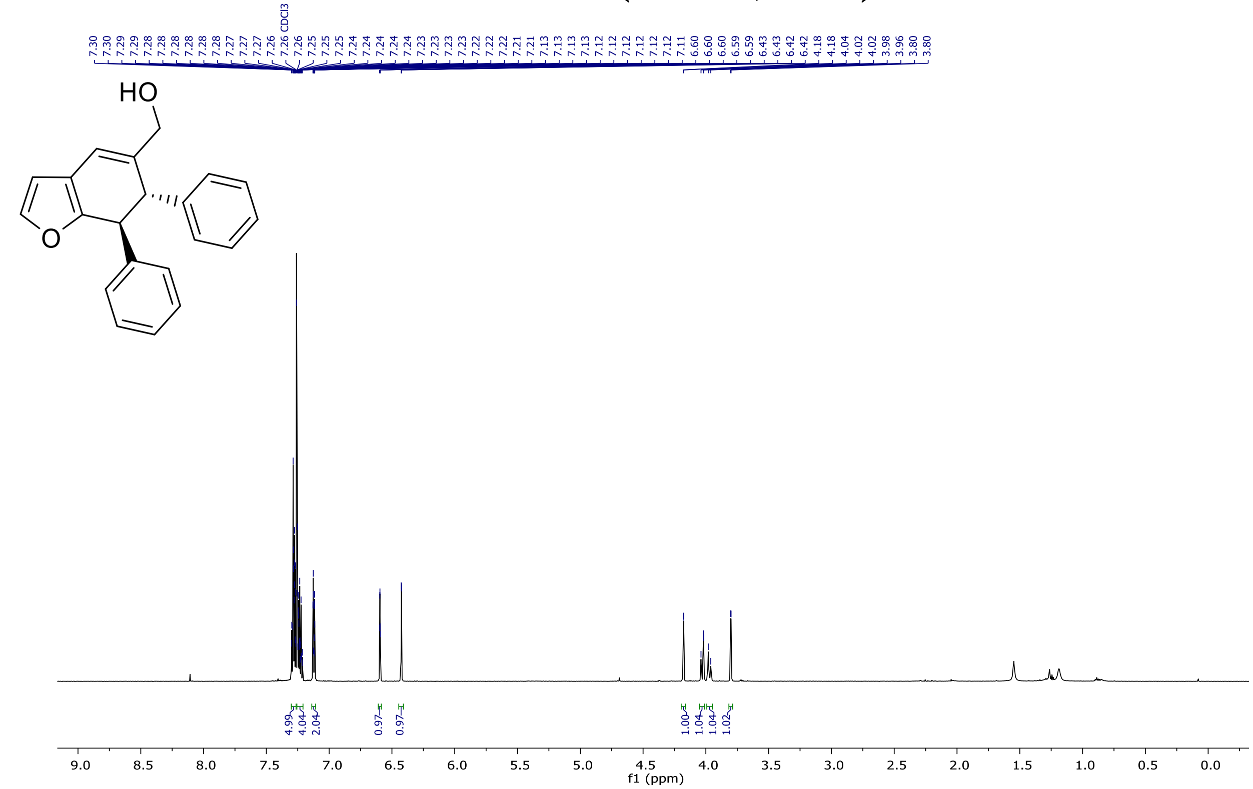

^{13}C NMR (176 MHz, $CDCl_3$)

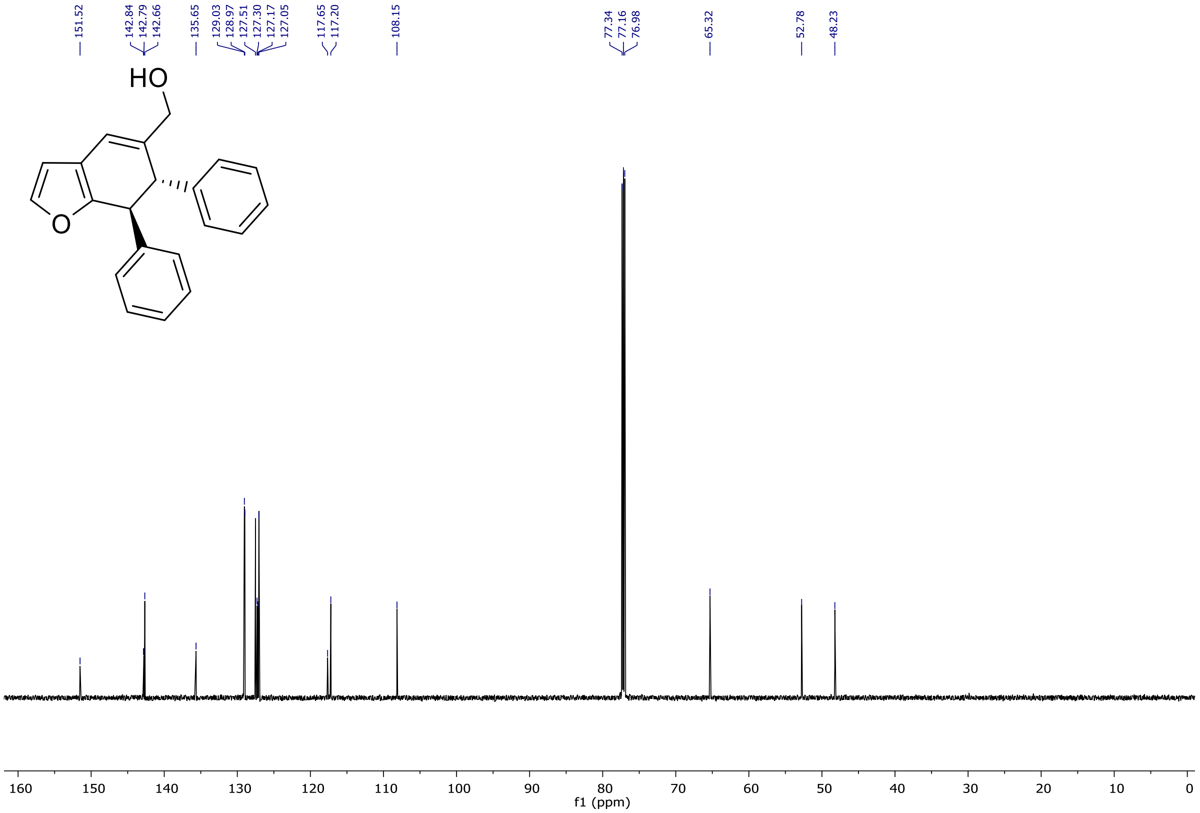

(4*S*,5*S*)-4,5-Diphenyl-5,12-dihydro-4*H*-benzo[*b*]benzofuro[4,5-*e*][1,4]diazepine 8.

^{1}H NMR (700 MHz, $CDCl_3$)

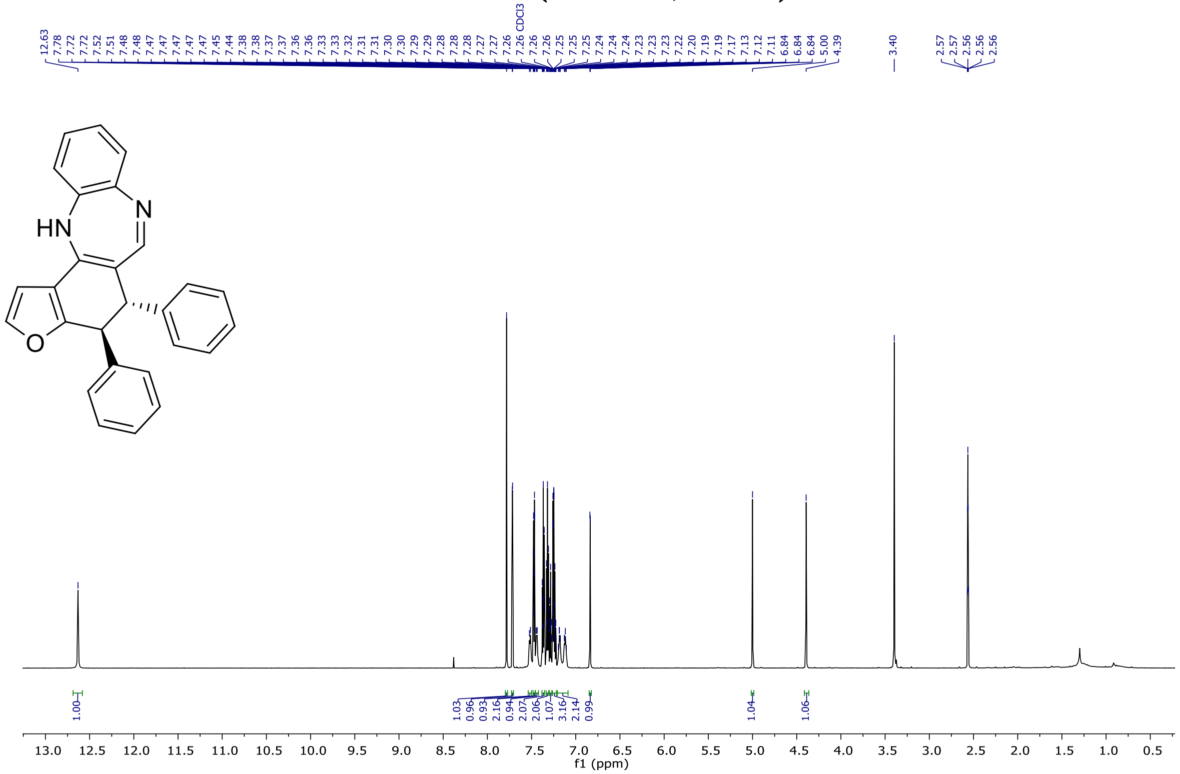

^{13}C NMR (176 MHz, $CDCl_3$)

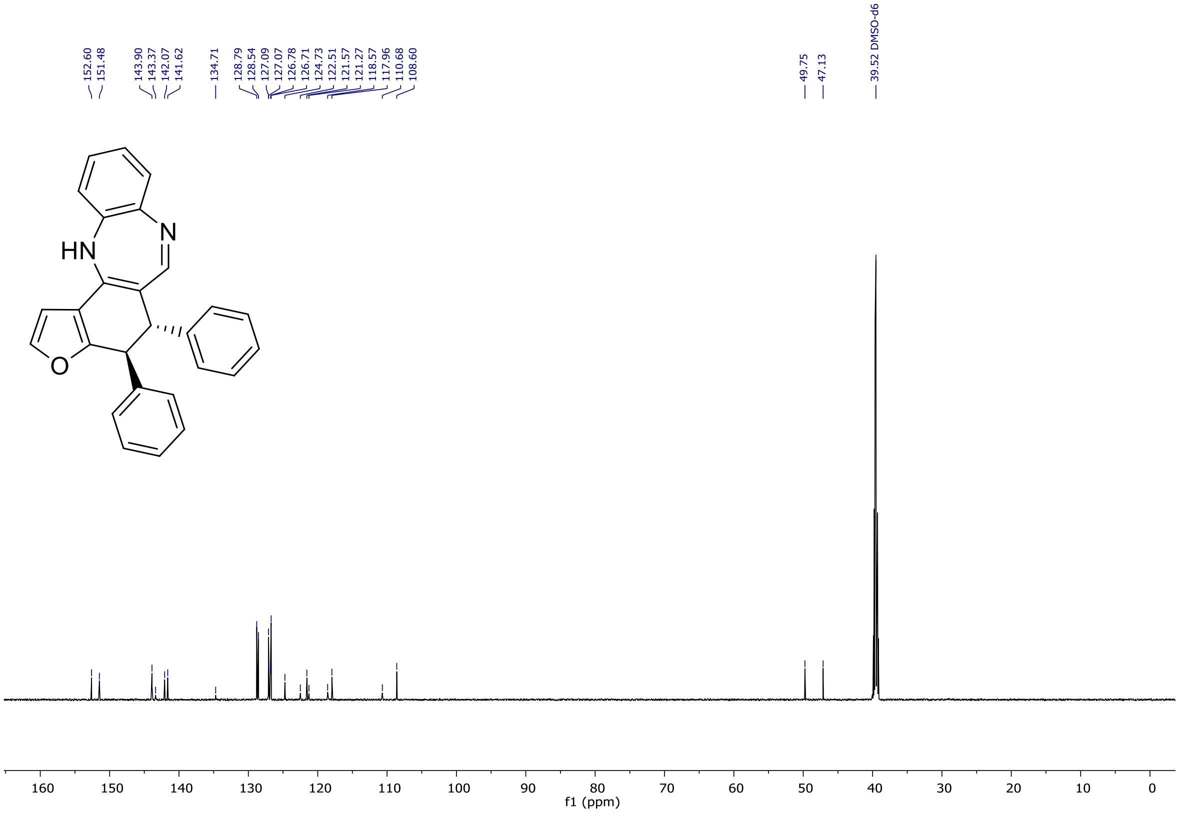

7. UPC^2 Data

(6*S*,7*S*)-6,7-Diphenyl-6,7-dihydrobenzofuran-5-carbaldehyde 3a

Racemic sample

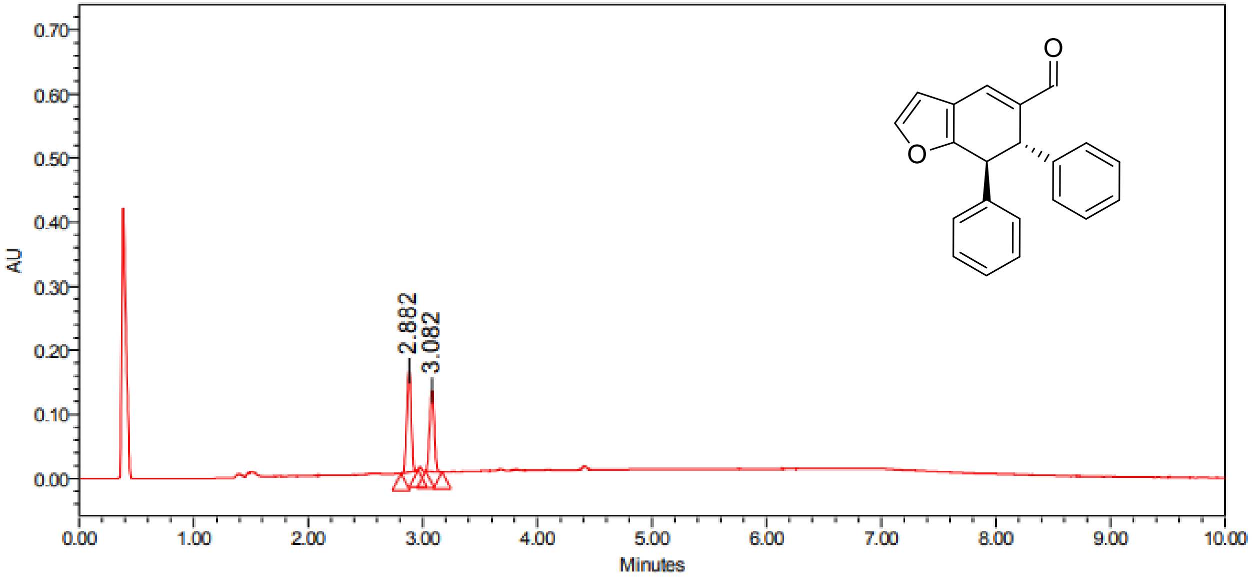

Peak Results

	RT	% Area
1	2.882	54.93
2	3.082	45.07

Enantiomerically enriched sample

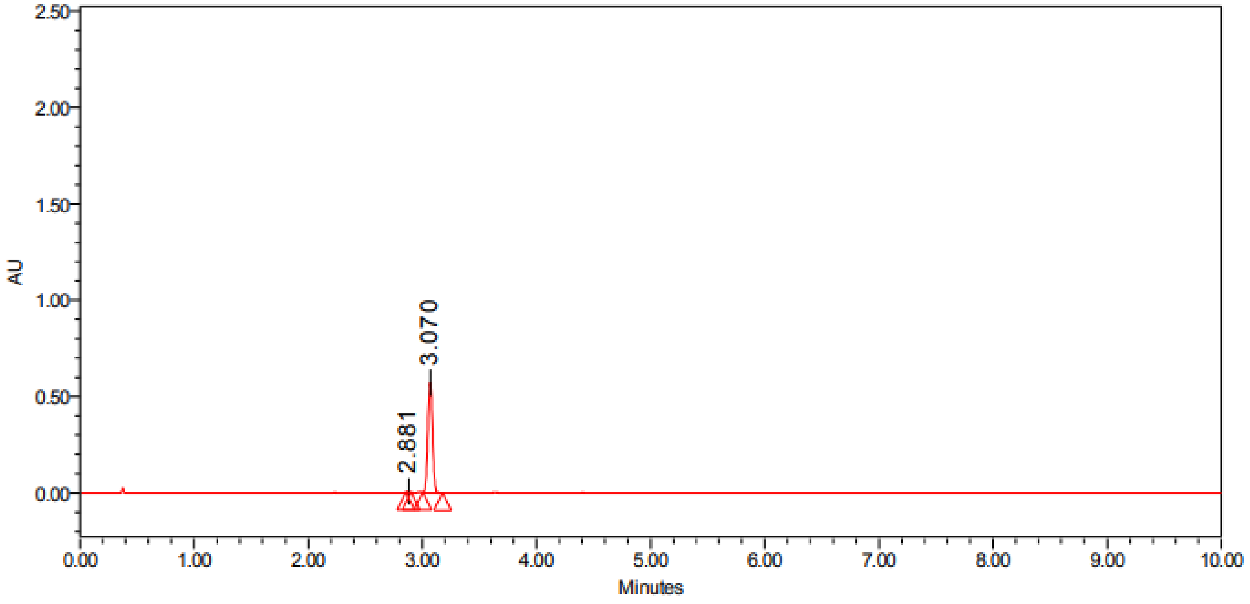

Peak Results

	RT	% Area
1	2.881	0.44
2	3.070	99.56

(6*S*,7*S*)-6-(4-Nitrophenyl)-7-phenyl-6,7-dihydrobenzofuran-5-carbaldehyde 3b

Racemic sample

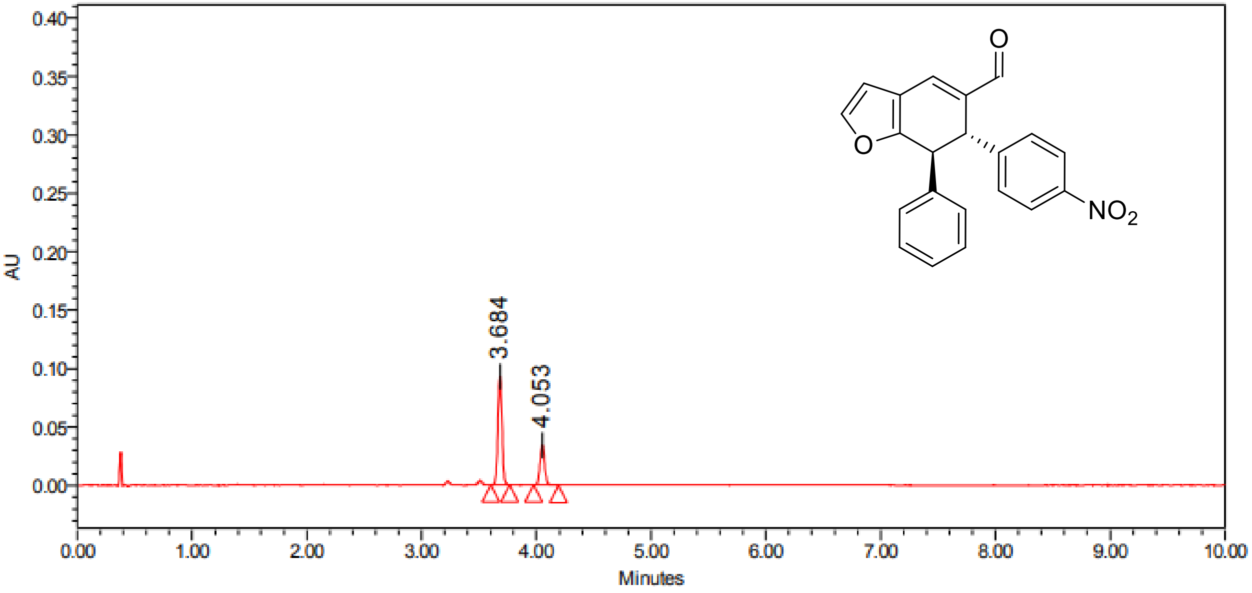

Peak Results

	RT	% Area
1	3.684	70.99
2	4.053	29.01

Enantiomerically enriched sample

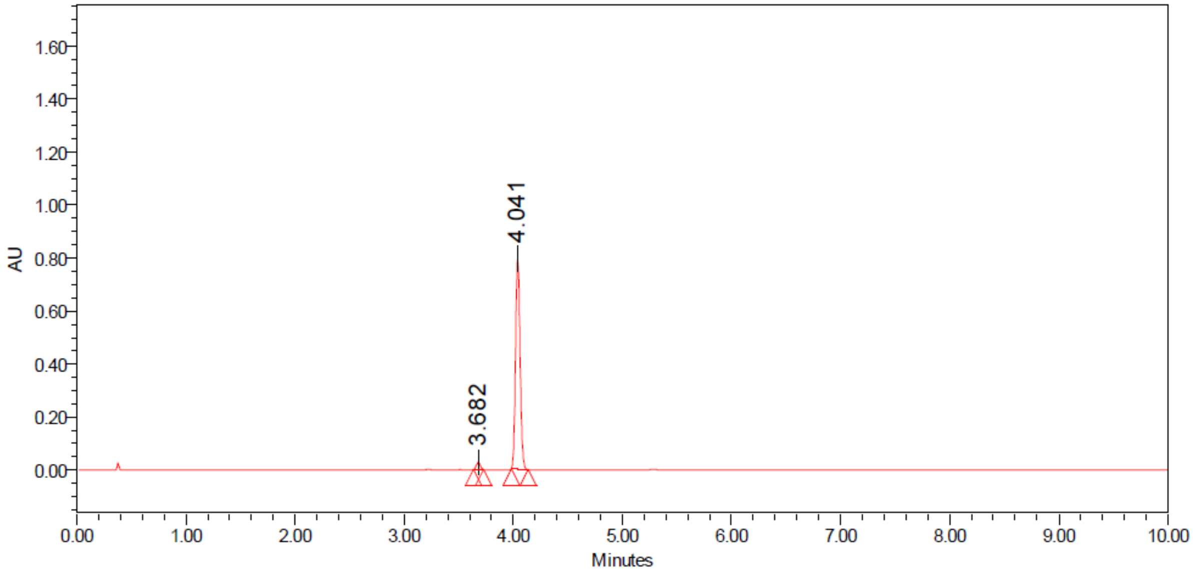

Peak Results

	RT	% Area
1	3.682	2.83
2	4.041	97.17

(6*S*,7*S*)-6-(4-Chlorophenyl)-7-phenyl-6,7-dihydrobenzofuran-5-carbaldehyde 3c

Racemic sample

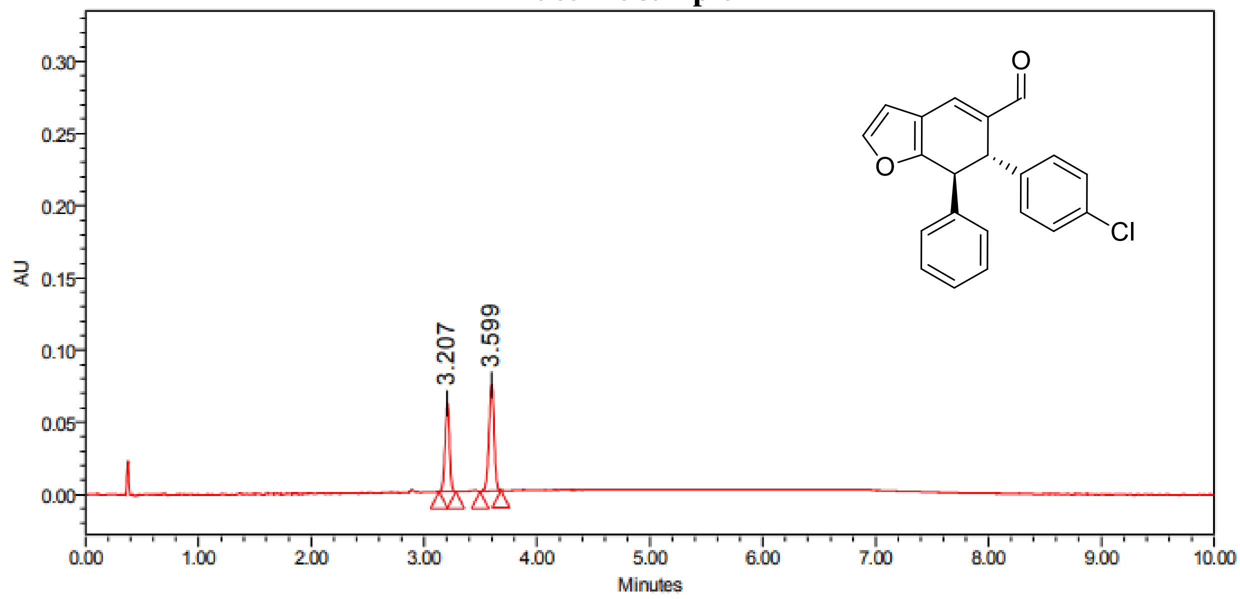

Peak Results

	RT	% Area
1	3.207	41.88
2	3.599	58.12

Enantiomerically enriched sample

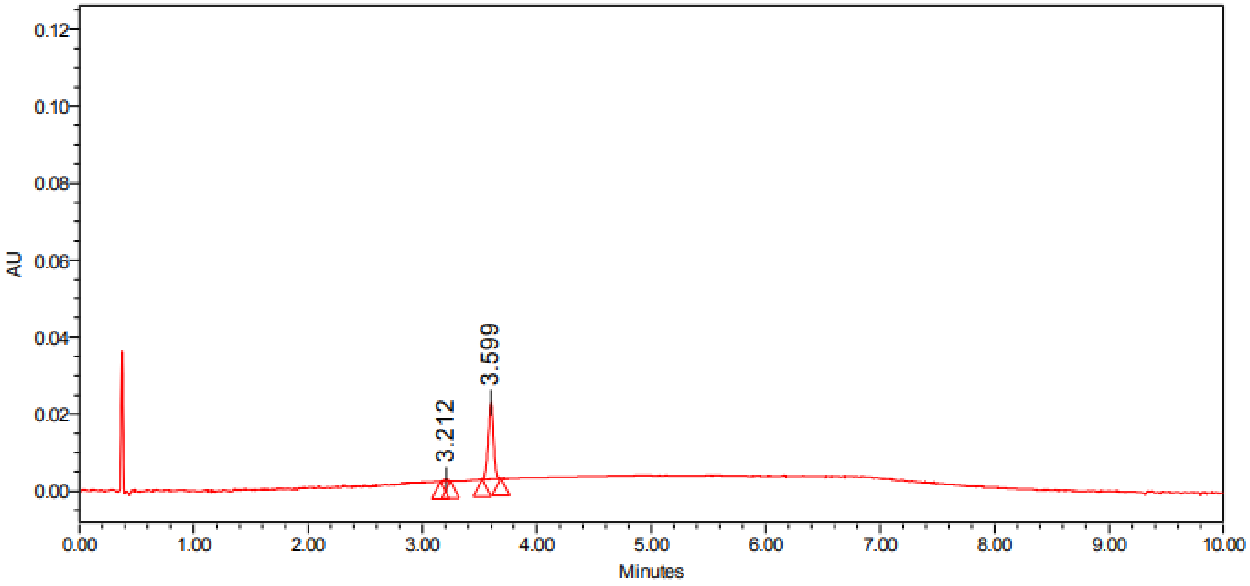

Peak Results

	RT	% Area
1	3.212	2.18
2	3.599	97.82

(6*S*,7*S*)-7-Phenyl-6-(p-tolyl)-6,7-dihydrobenzofuran-5-carbaldehyde 3d

Racemic sample

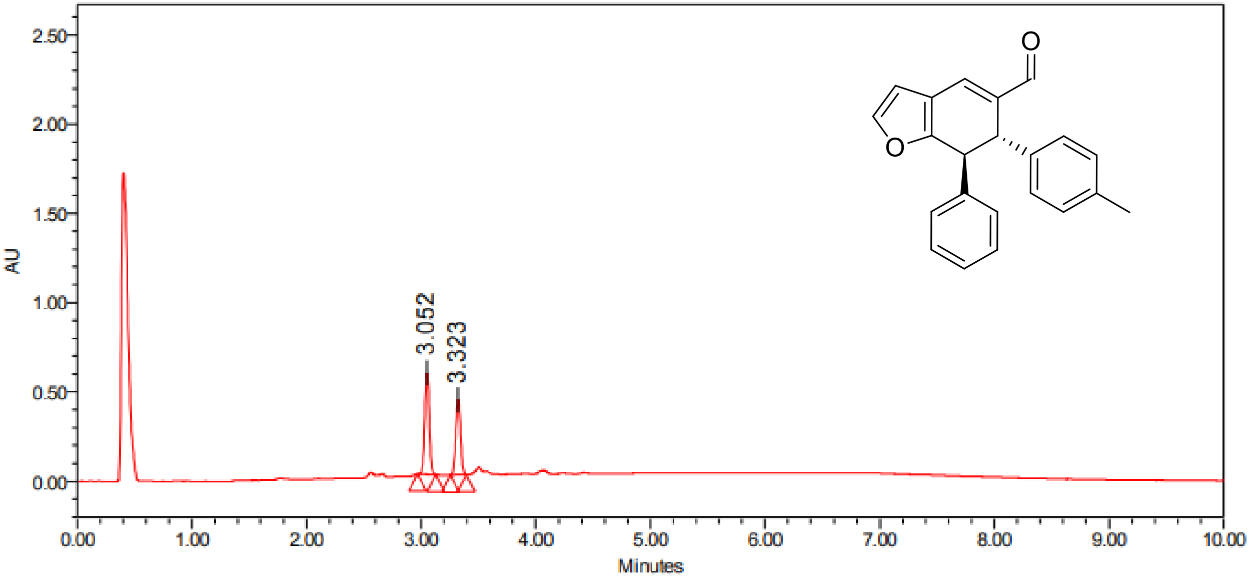

Peak Results

	RT	% Area
1	3.052	55.83
2	3.323	44.17

Enantiomerically enriched sample

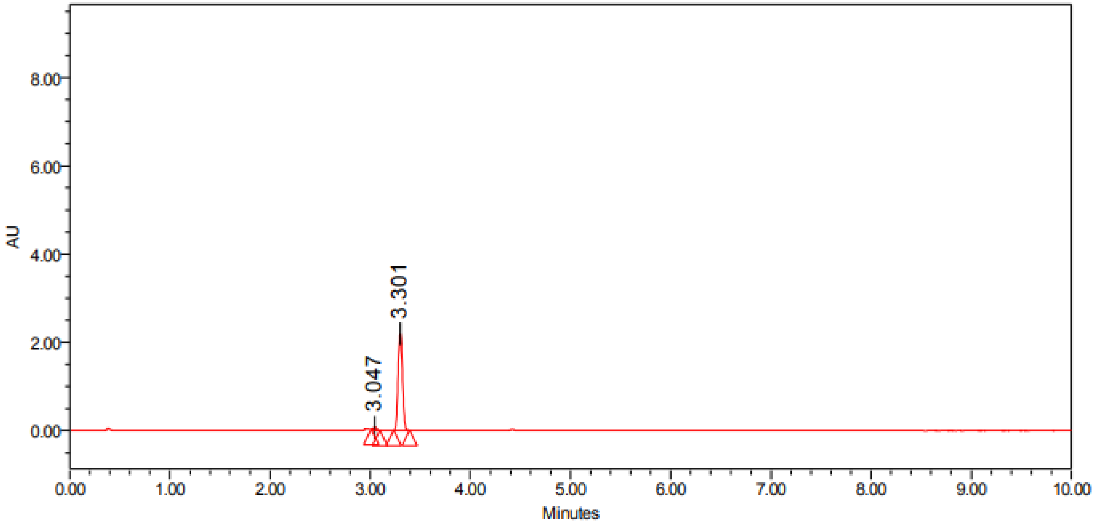

Peak Results

	RT	% Area
1	3.047	1.90
2	3.301	98.10

(6*S*,7*S*)-6-(4-Methoxyphenyl)-7-phenyl-6,7-dihydrobenzofuran-5-carbaldehyde 3e

Racemic sample

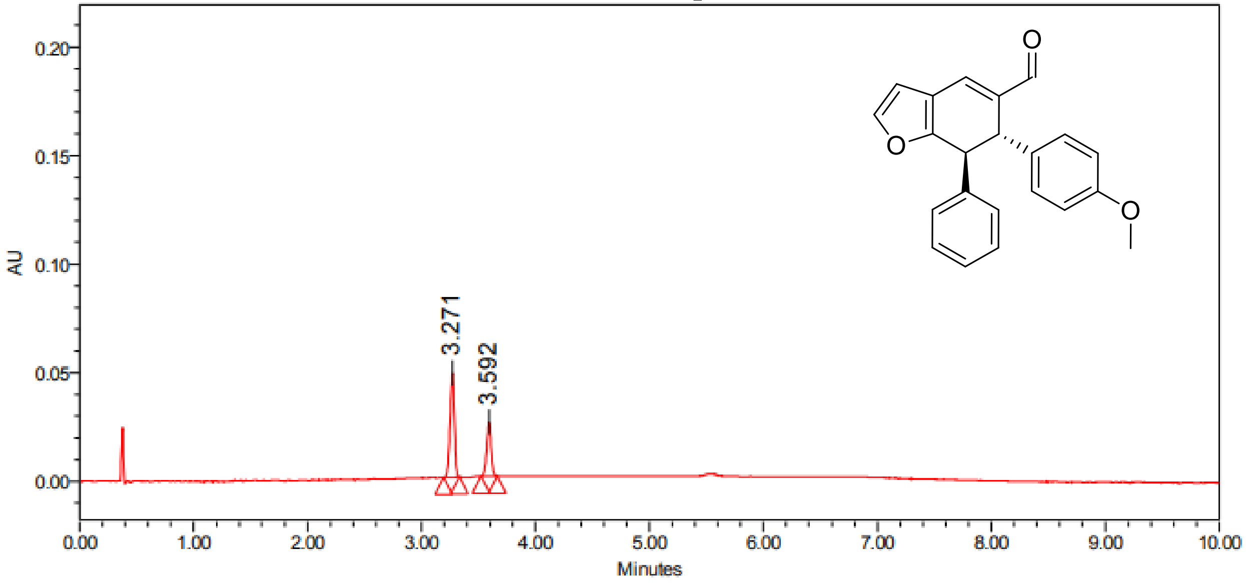

Peak Results

	RT	% Area
1	3.271	64.19
2	3.592	35.81

Enantiomerically enriched sample

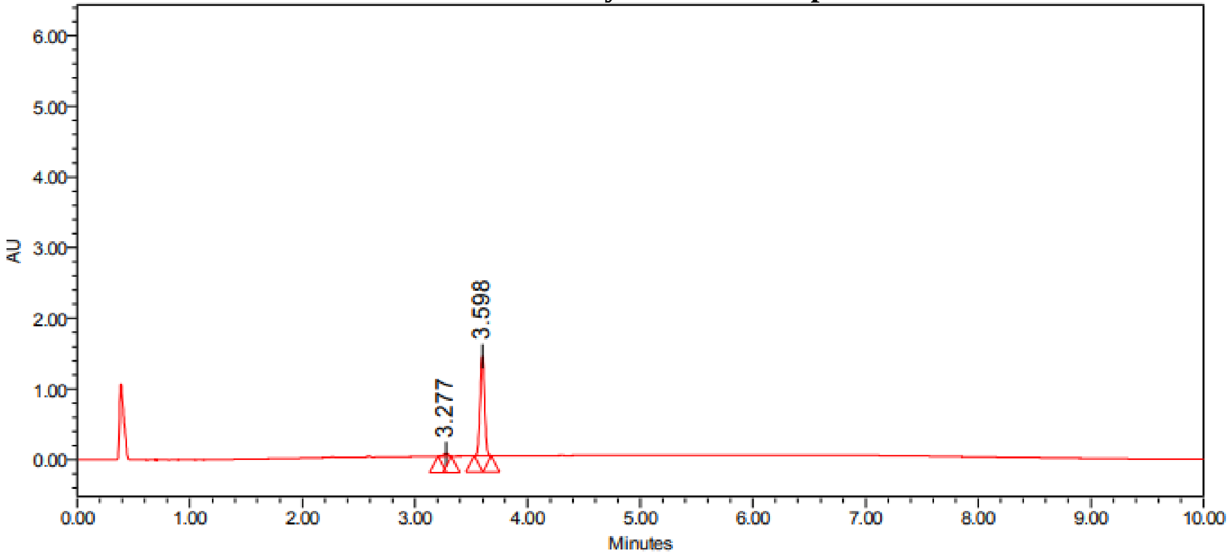

Peak Results

	RT	% Area
1	3.277	2.33
2	3.598	97.67

(6*S*,7*S*)-6-(3-Methoxyphenyl)-7-phenyl-6,7-dihydrobenzofuran-5-carbaldehyde 3f

Racemic sample

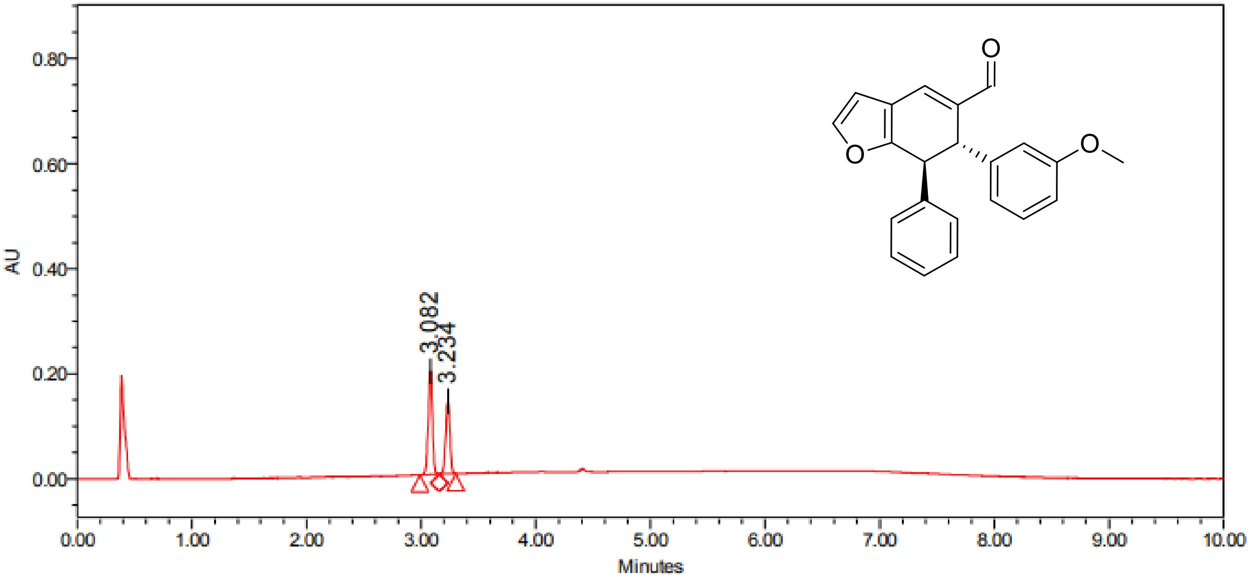

Peak Results

	RT	% Area
1	3.082	58.55
2	3.234	41.45

Enantiomerically enriched sample

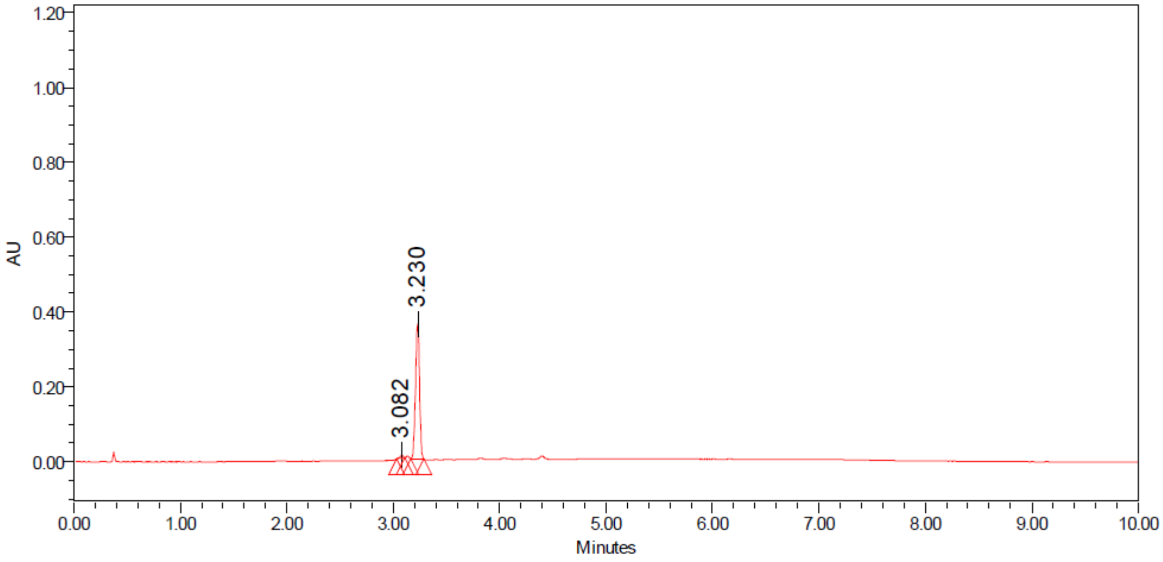

Peak Results

	RT	% Area
1	3.082	1.72
2	3.230	98.28

(6*S*,7*S*)-6-(2-Methoxyphenyl)-7-phenyl-6,7-dihydrobenzofuran-5-carbaldehyde 3g

Racemic sample

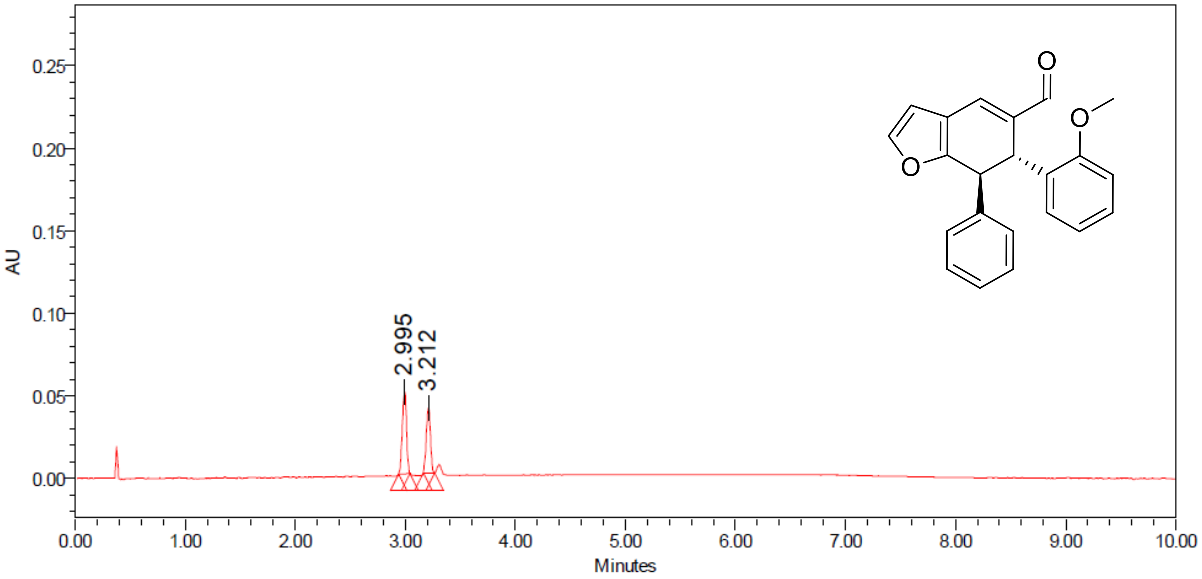

Peak Results

	RT	% Area
1	2.995	56.31
2	3.212	43.69

Enantiomerically enriched sample

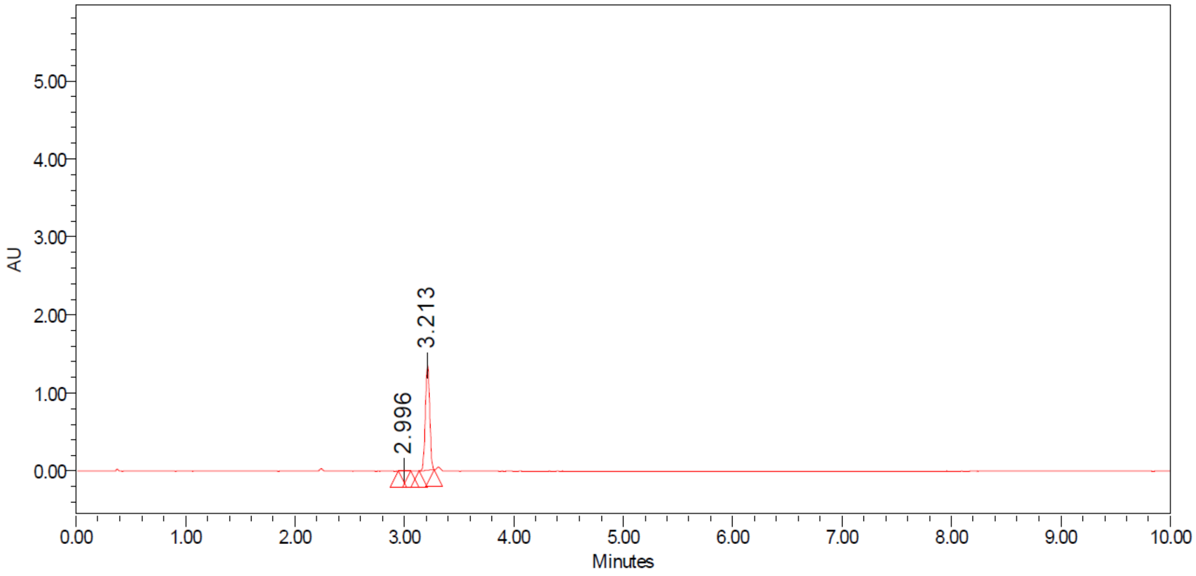

Peak Results

	RT	% Area
1	2.996	0.37
2	3.213	99.63

(6*R*,7*S*)-6-(2,4-Dichlorophenyl)-7-phenyl-6,7-dihydrobenzofuran-5-carbaldehyde 3h

Racemic sample

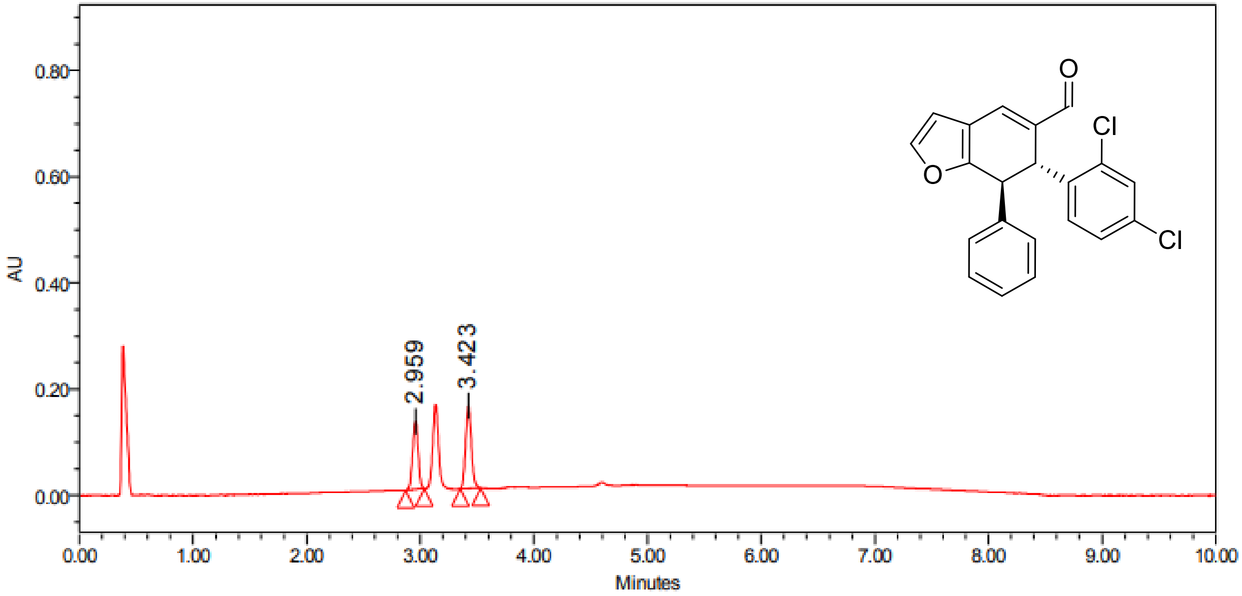

Peak Results

	RT	% Area
1	2.959	43.84
2	3.423	56.16

Enantiomerically enriched sample

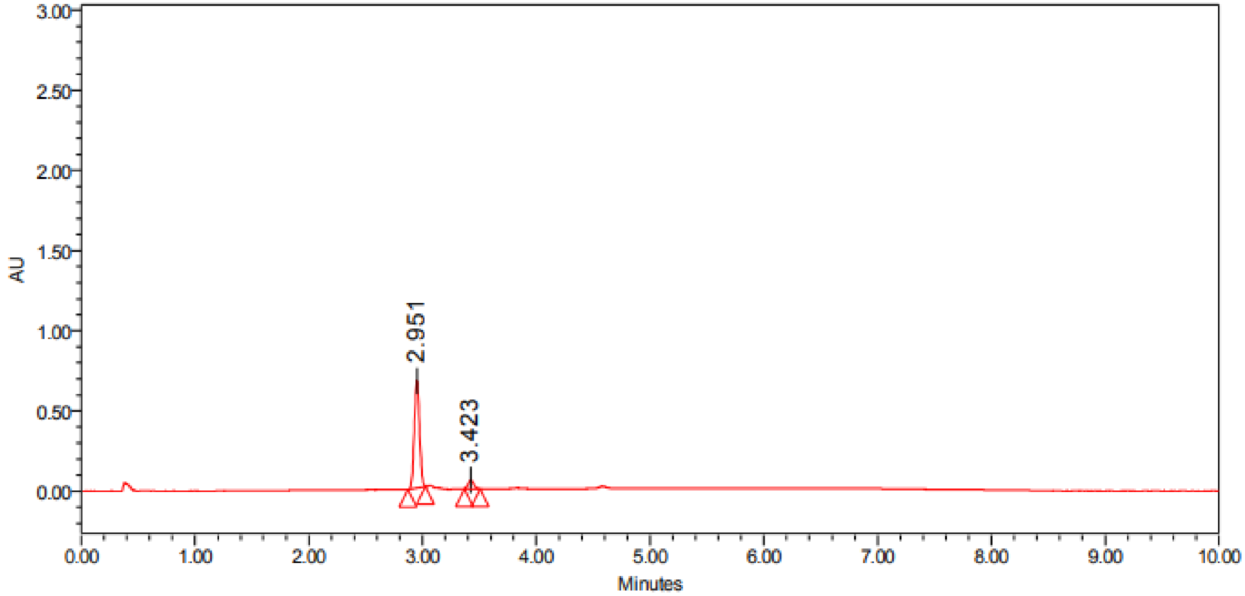

Peak Results

	RT	% Area
1	2.951	90.14
2	3.423	9.86

(6*R*,7*S*)-6-(Furan-2-yl)-7-phenyl-6,7-dihydrobenzofuran-5-carbaldehyde 3i

Racemic sample

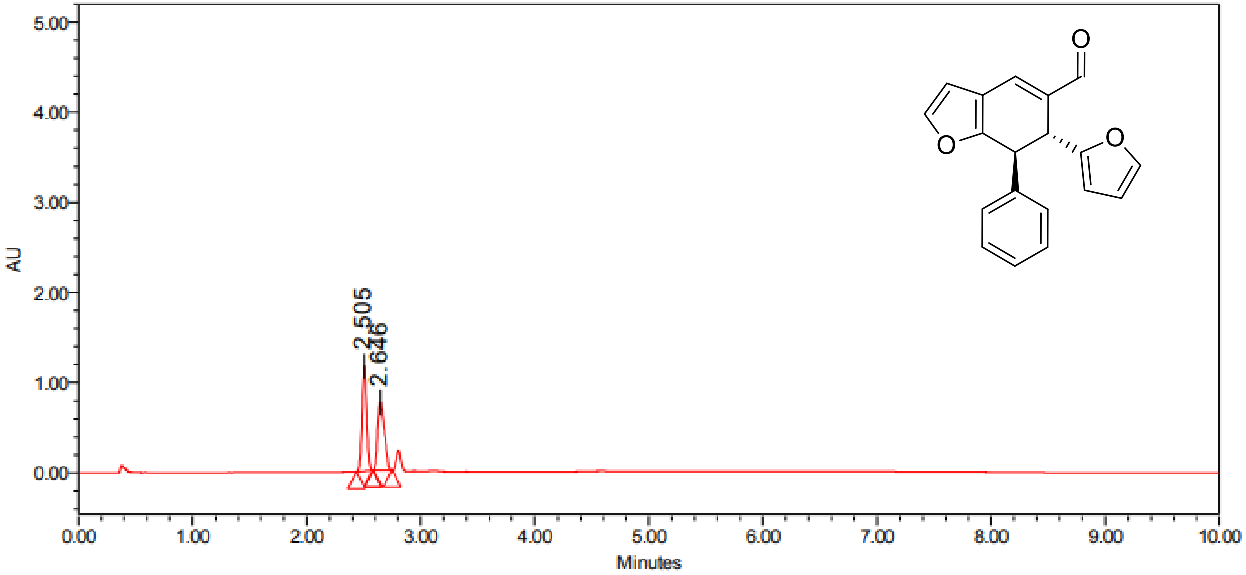

Peak Results

	RT	% Area
1	2.505	52.87
2	2.646	47.13

Enantiomerically enriched sample

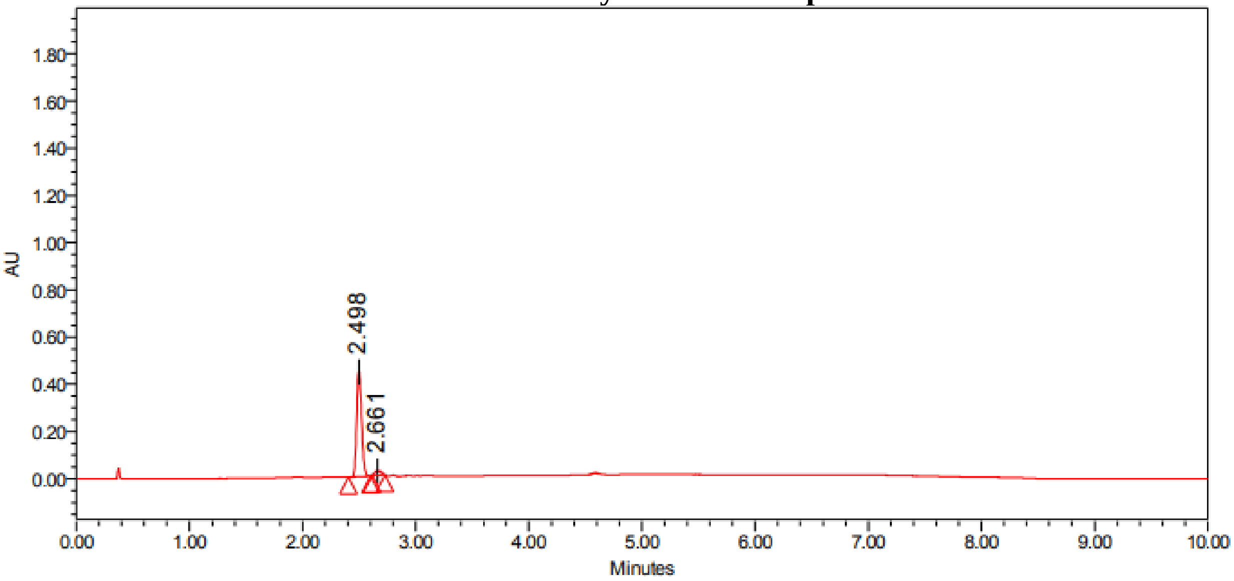

Peak Results

	RT	% Area
1	2.498	94.68
2	2.661	5.32

(6*S*,7*S*)-7-(4-Fluorophenyl)-6-phenyl-6,7-dihydrobenzofuran-5-carbaldehyde 3j

Racemic sample

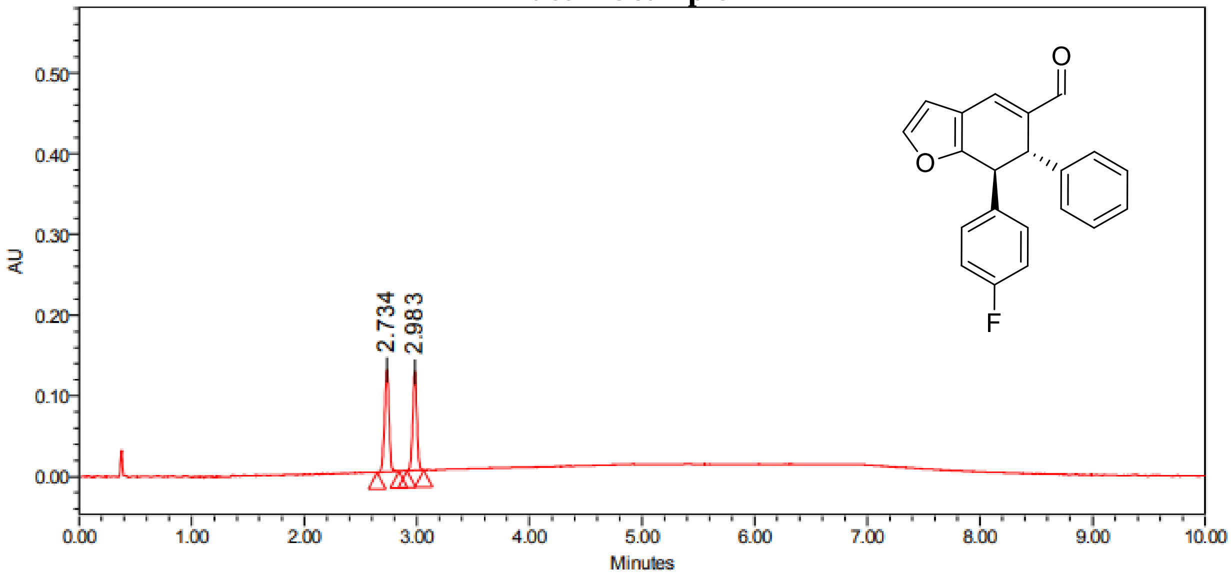

Peak Results

	RT	% Area
1	2.734	52.50
2	2.983	47.50

Enantiomerically enriched sample

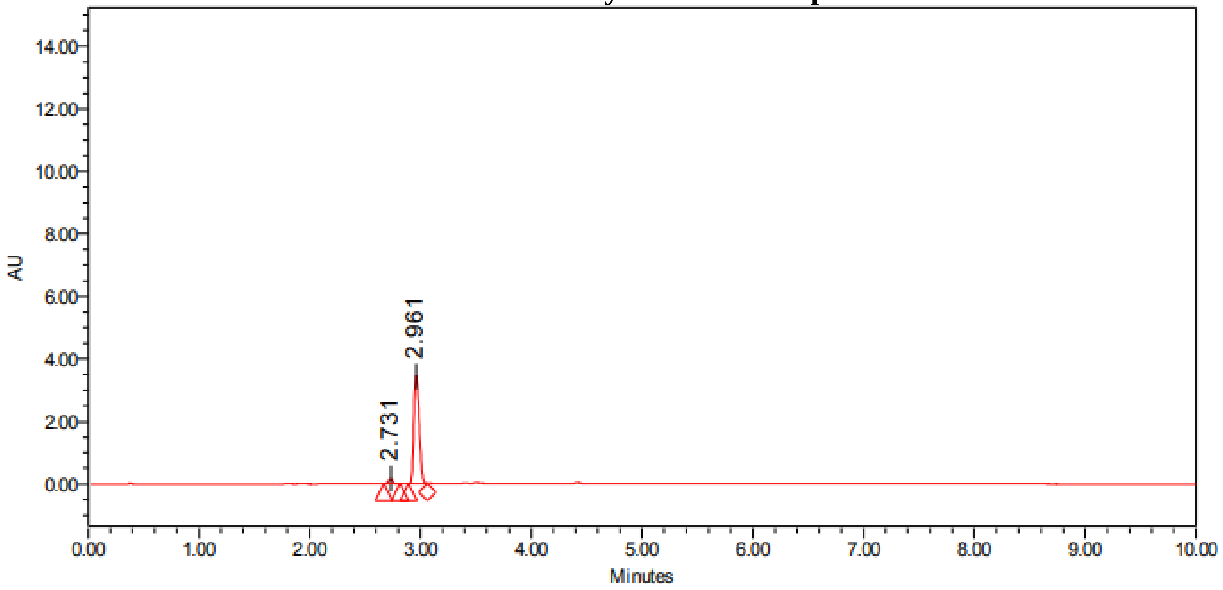

Peak Results

	RT	% Area
1	2.731	3.27
2	2.961	96.73

(6*S*,7*S*)-6-Phenyl-7-(p-tolyl)-6,7-dihydrobenzofuran-5-carbaldehyde 3k

Racemic sample

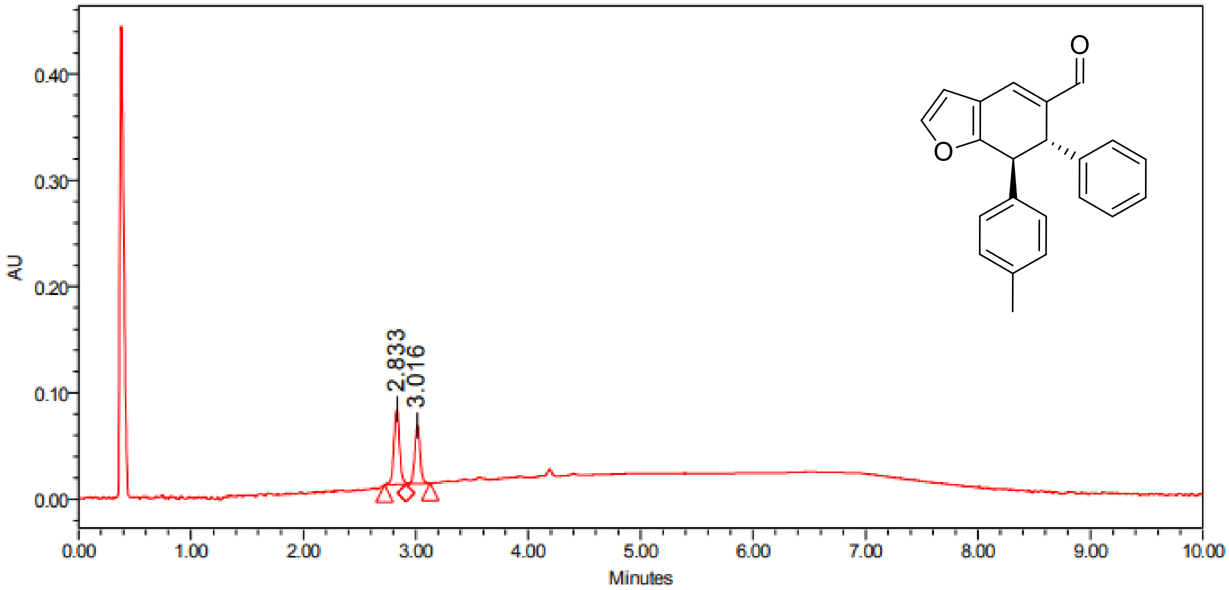

Peak Results

	RT	% Area
1	2.833	58.54
2	3.016	41.46

Enantiomerically enriched sample

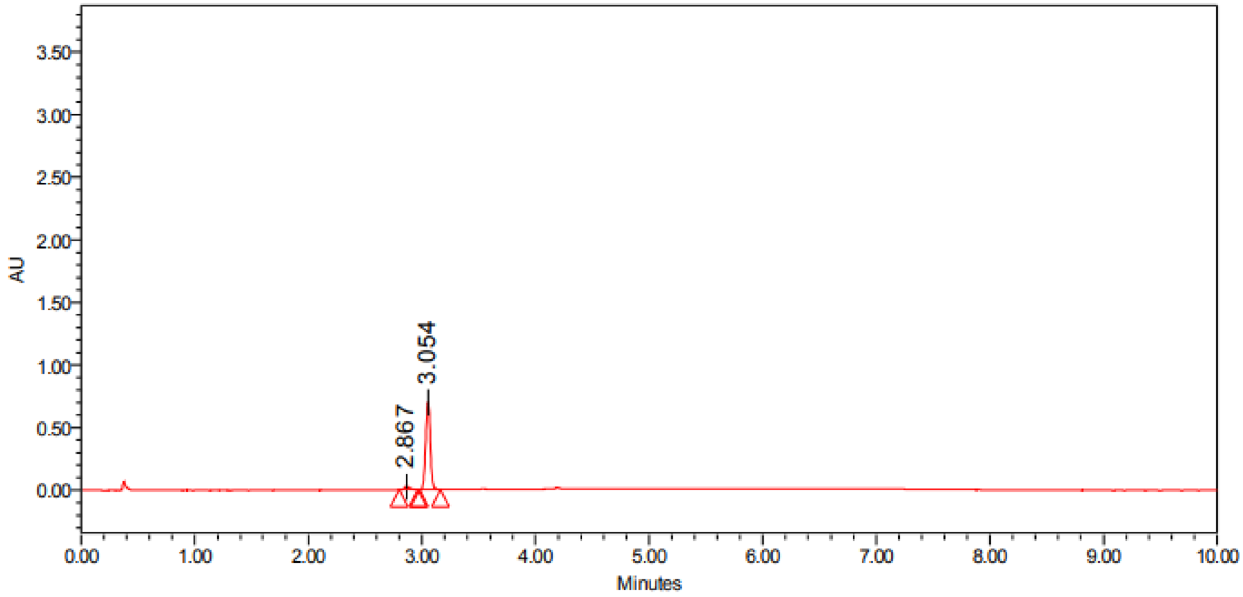

Peak Results

	RT	% Area
1	2.867	3.73
2	3.054	96.27

(6*S*,7*S*)-7-(3-Methoxyphenyl)-6-phenyl-6,7-dihydrobenzofuran-5-carbaldehyde 3l

Racemic sample

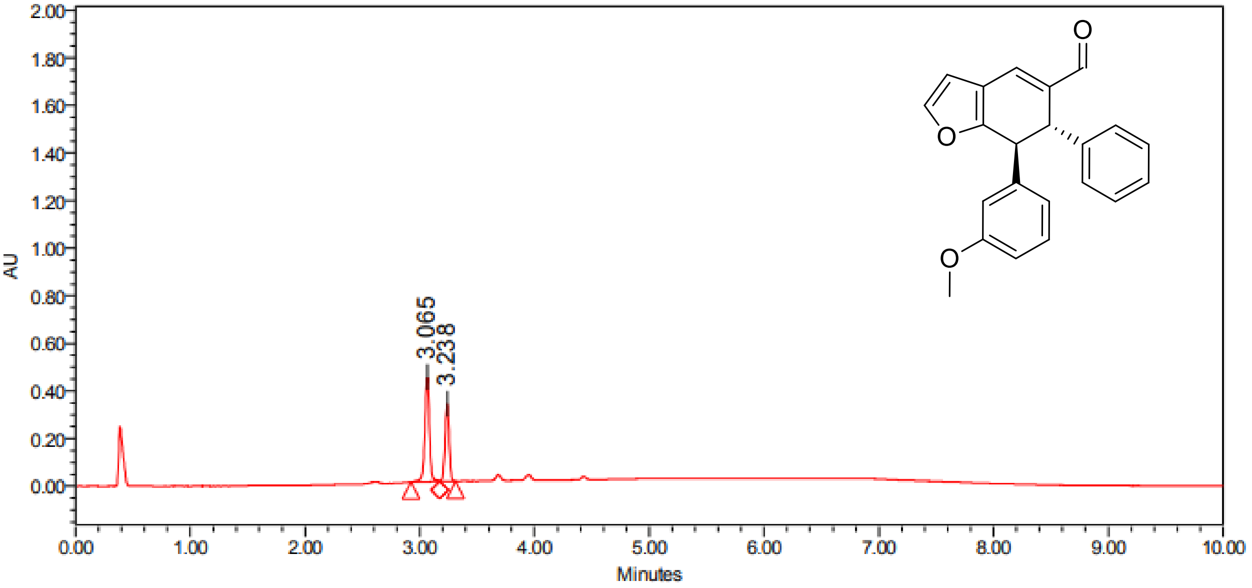

Peak Results

	RT	% Area
1	3.065	60.42
2	3.238	39.58

Enantiomerically enriched sample

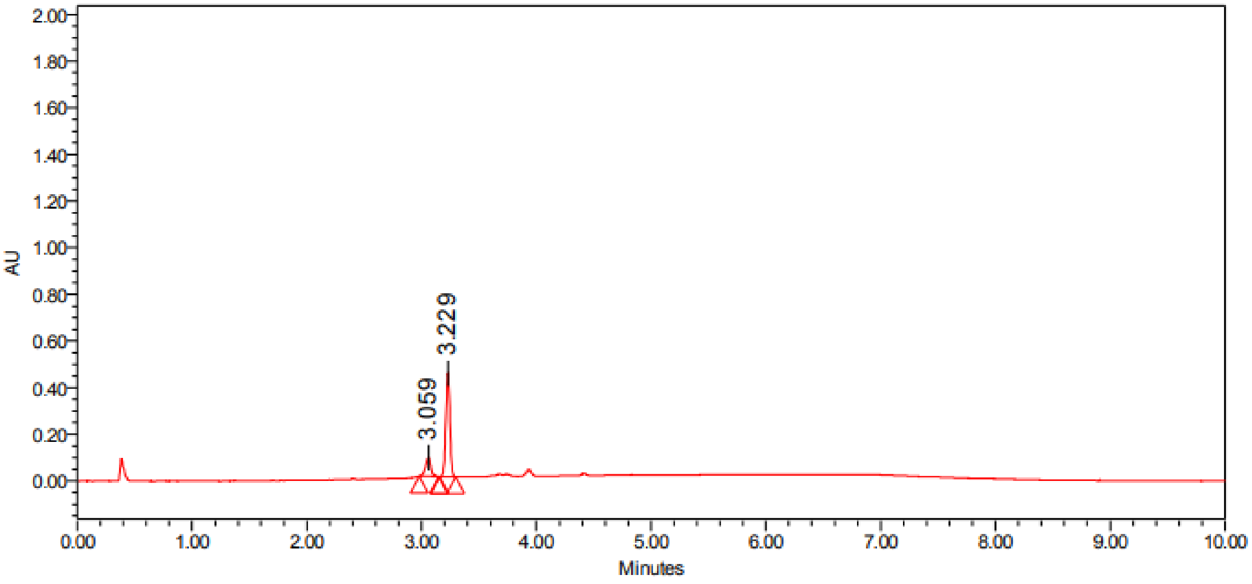

Peak Results

	RT	% Area
1	3.059	18.99
2	3.229	81.01

(6*S*,7*S*)-6-Phenyl-7-(o-tolyl)-6,7-dihydrobenzofuran-5-carbaldehyde 3m

Racemic sample

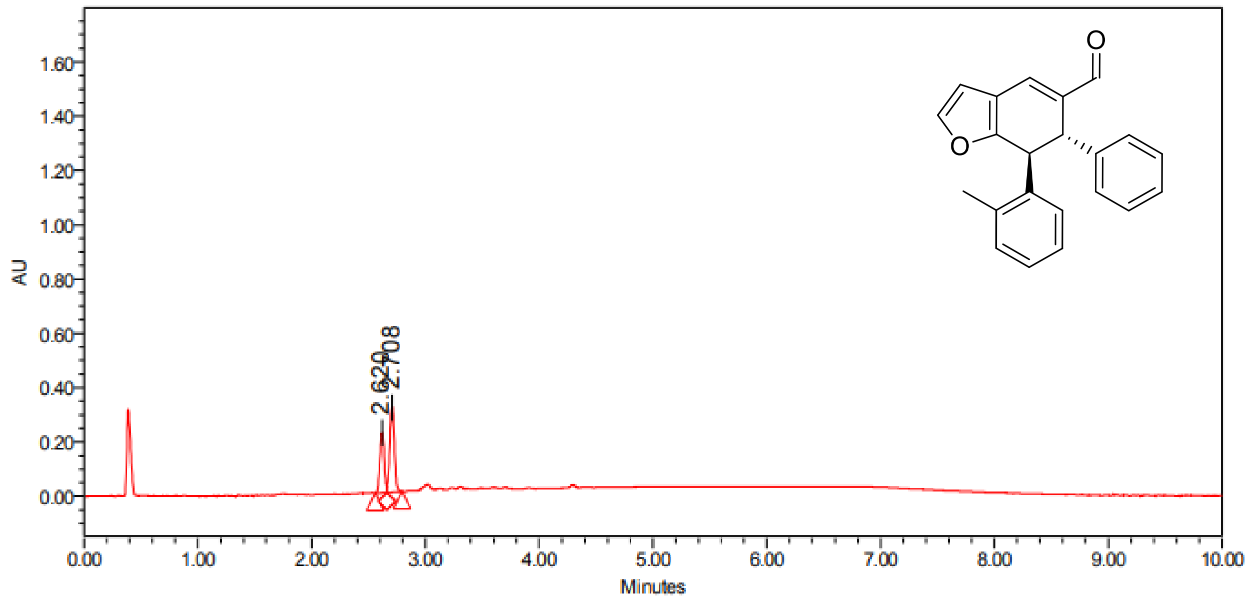

Peak Results

	RT	% Area
1	2.619	39.92
2	2.708	60.08

Enantiomerically enriched sample

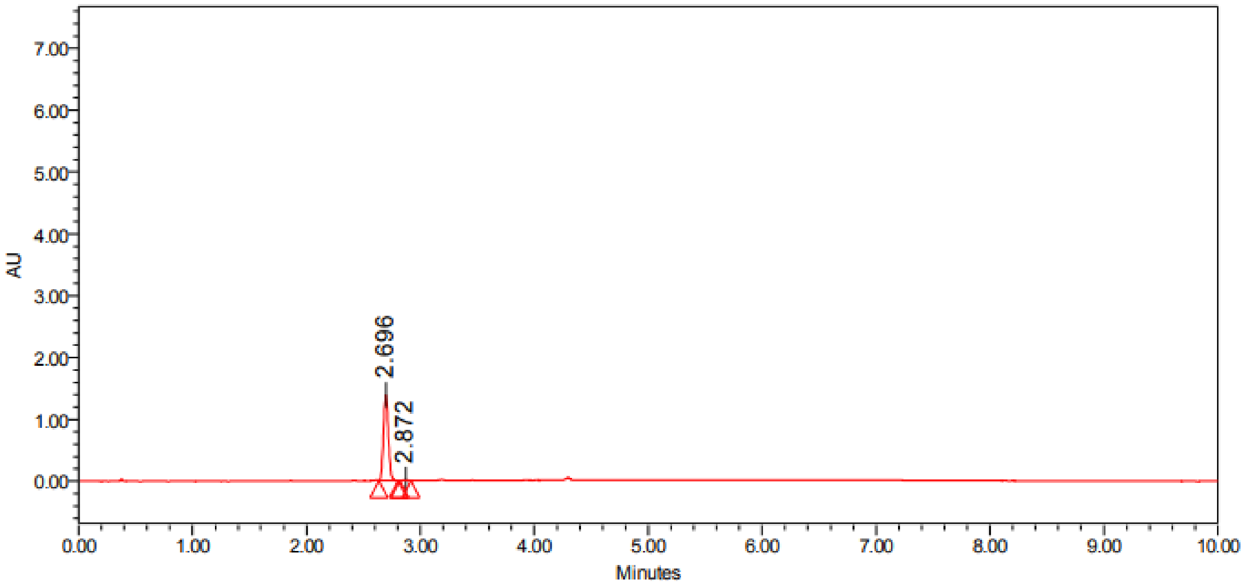

Peak Results

	RT	% Area
1	2.696	99.42
2	2.866	0.58

(6*S*,7*R*)-6-Phenyl-7-vinyl-6,7-dihydrobenzofuran-5-carbaldehyde 3n

Racemic sample

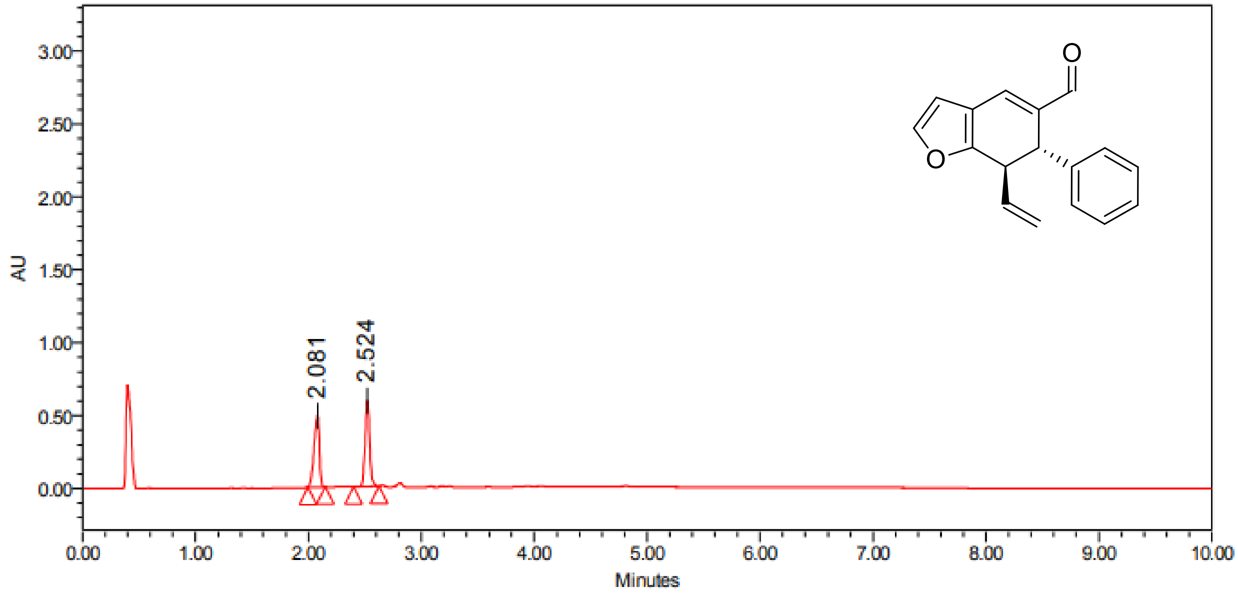

Peak Results

	RT	% Area
1	2.081	49.23
2	2.524	50.77

Enantiomerically enriched sample

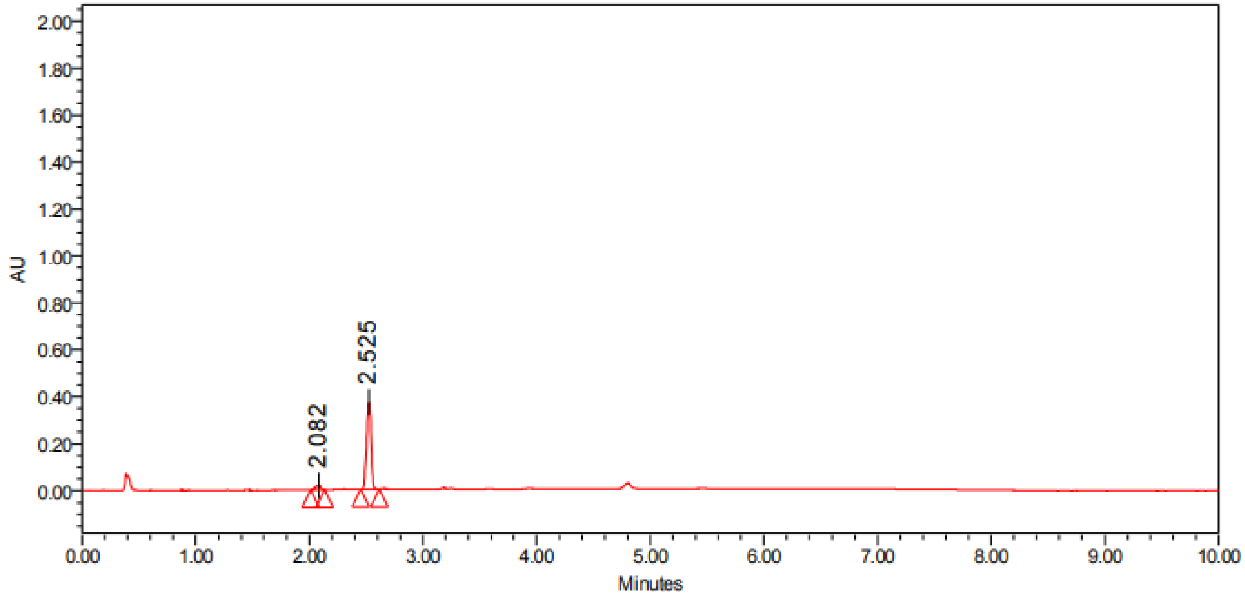

Peak Results

	RT	% Area
1	2.082	5.56
2	2.525	94.44

(3*S*,4*S*)-3,4-Diphenyl-3,4-dihydrodibenzo[b,d]furan-2-carbaldehyde 3o

Racemic sample

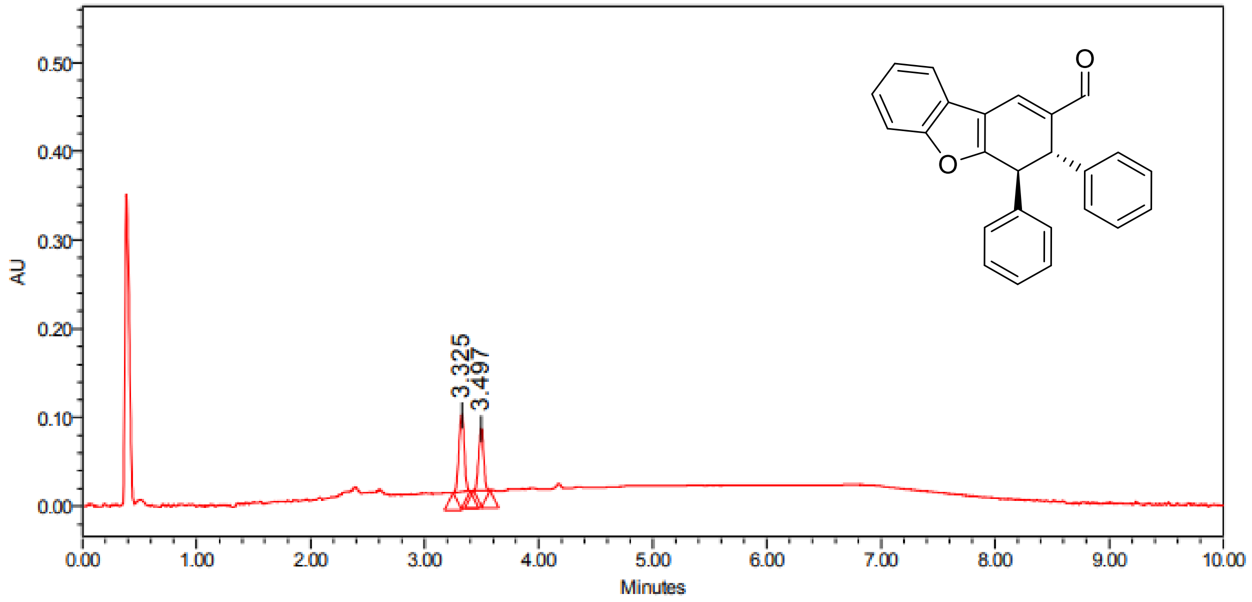

Peak Results

	RT	% Area
1	3.325	57.36
2	3.497	42.64

Enantiomerically enriched sample

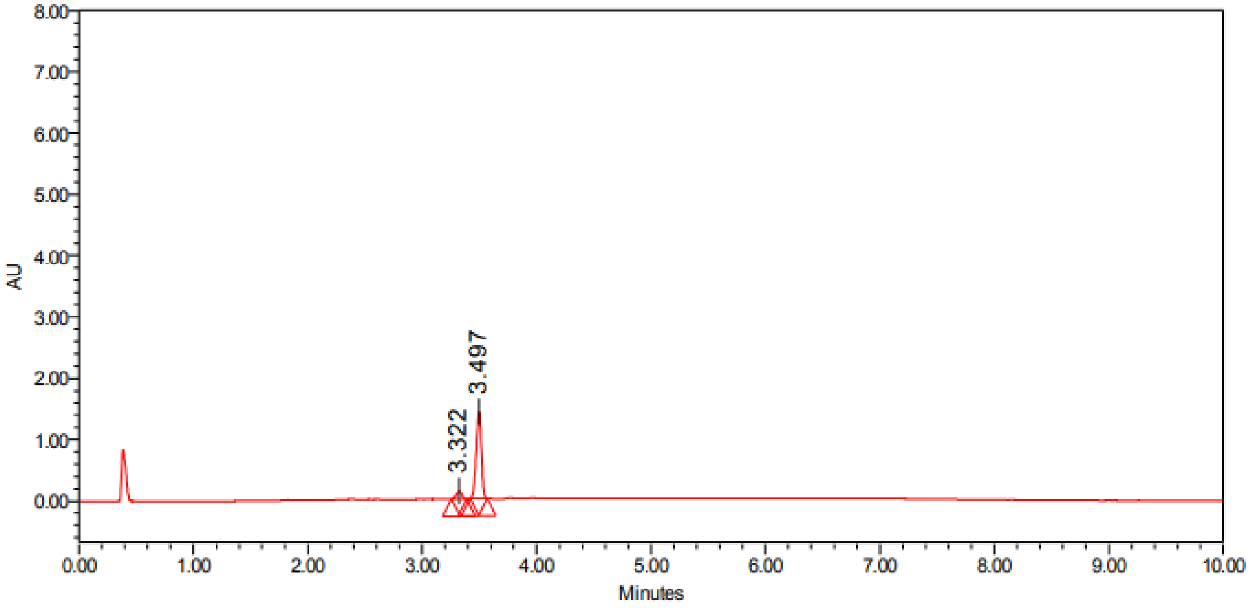

Peak Results

	RT	% Area
1	3.322	8.07
2	3.497	91.93
